# Bifunctional Metformin–Phenolic Hybrids with Improved Anticancer and Antioxidant Properties: Evaluation on Glioma Cells

**DOI:** 10.3390/ijms27031259

**Published:** 2026-01-27

**Authors:** Caroline Delehedde, Mathieu Chocry, Camille Nguyen, Alice Asteian, Maxime Robin, Ludovic Leloup, Mathieu Cassien, Anne Mercier, Marcel Culcasi, Hervé Kovacic, Sylvia Pietri

**Affiliations:** 1 Aix Marseille Université, CNRS, Institut de Chimie Radicalaire (ICR), UMR 7273, SMBSO, 13397 Marseille, Francecamille.nguyen@univ-amu.fr (C.N.); maxime.robin@univ-amu.fr (M.R.); mathieu.cassien@univ-amu.fr (M.C.); anne.mercier@univ-amu.fr (A.M.); marcel.culcasi@univ-amu.fr (M.C.); 2 Aix Marseille Université, CNRS, Institut de Neurophysiopathologie (INP), UMR 7051, 13005 Marseille, France; ludovic.leloup@univ-amu.fr (L.L.); herve.kovacic@univ-amu.fr (H.K.); 3 Yelen Analytics, 13820 Ensuès-la-Redonne, France

**Keywords:** glioblastoma, hybrid molecules, metformin, phenolics, cytotoxicity, oxidative stress, kinase activity

## Abstract

Glioblastoma is one of the most highly aggressive types of brain tumor in adults. With limited treatment options, current therapies remain insufficient due to its invasiveness and immune evasion, highlighting the urgent need for new treatments. Bifunctional molecules targeting multiple aspects of the disease could be promising to overcome drug resistance and tumor heterogeneity. Metformin has demonstrated protective effects against brain tumors but requires high doses for efficacy, making it of great interest for molecular optimization. In this context, we synthesized a series of nine metformin–phenolic molecules, combining the metformin guanidine framework with phenolic acids, which have well-established properties in inhibiting cancer cell migration and adhesion. Their impact on cytotoxicity, reactive oxygen species inhibition, and signaling pathways was investigated for glioma cell lines and stem cells. Two of these hybrids, **5a** and **5h,** particularly enhanced cytotoxicity in glioblastoma cells, selectively targeting cancer cells while sparing healthy ones. Their mechanism of action differed significantly from metformin. Unlike metformin, which mainly triggers metabolic stress, the hybrids broadly inhibit RTK–MAPK–PI3K signaling, leading to cell cycle arrest and apoptosis. The results suggest that these compounds could offer a more effective and synergistic approach for glioblastoma treatment.

## 1. Introduction

Glioblastomas (GBMs), classified as grade IV gliomas, are the most common and aggressive primary tumors of the central nervous system. They are highly vascular (angiogenic) and associated with a poor prognosis, with a median survival of only 15 months [1]. These tumors are characterized by multiple genetic alterations that promote aggressive behavior. This combination of genetic changes drives both the rapid growth and invasion in GBM [2], ultimately increasing metabolic demand, which contributes to elevated oxidative stress in tumor cells.

Despite advances in surgical techniques, radiotherapy, and chemotherapy, including anti-angiogenic therapies such as Bevacizumab, GBM remains incurable. Increasing evidence indicates that therapeutic resistance in GBM is associated with the metabolic reprogramming and mitochondrial-mediated redox adaptation of tumor cells [3]. GBM cells display profound alterations in mitochondrial function that sustain high energetic demands and support tumor growth. Mitochondrial respiration is essential for GBM progression [4] and represents a major source of reactive oxygen species (ROS), which act as signaling molecules regulating proliferation, survival, and invasion. Rather than being merely toxic by-products, ROS contribute to oncogenic signaling when maintained at controlled levels. In GBM cells, efficient antioxidant systems buffer excessive oxidative stress while preserving ROS-dependent signaling pathways [5]. The disruption of mitochondrial respiration or redox homeostasis can exceed cellular defenses, resulting in an excessive ROS accumulation and activation of cell death pathways. Accordingly, targeting mitochondrial metabolism and redox regulation has emerged as a promising therapeutic strategy in glioblastoma [6].

Metformin, a widely used antidiabetic drug, has shown potential in glioblastoma treatment, as meta-analyses report a reduced cancer incidence in diabetic patients treated with metformin [7,8]. Although its exact mechanisms are still under investigation; metformin is believed to modulate cellular energy production and growth signaling pathways. Studies suggest that metformin can inhibit glioblastoma cell proliferation and potentially influence the tumor environment [9,10]. Metformin represents a potential new approach for glioblastoma treatment, warranting further research to optimize its therapeutic potential. Moreover, studies in animal and cellular models have confirmed its strong anti-proliferative effect on various cancers [11,12]. Several clinical trials are ongoing to evaluate the potential of metformin as an antitumor drug [13,14]. Mechanistically, metformin mainly acts on mitochondria, inhibiting mitochondrial respiration and activating AMP-activated protein kinase (AMPK), which inhibits the mammalian target of rapamycin (mTOR), a key factor in the interaction between metabolism and cancer [15]. Although metformin inhibits cancer cell proliferation, high doses are required, which raises concerns about its therapeutic feasibility. Therefore, developing metformin-derived molecules to enhance its antitumor potency represents a promising strategy.

Our laboratory has been developing hybrid molecules combining known antioxidants with a framework that presents pharmacological properties. Nitrones bearing an aryl substituent inspired by those found in natural phenolic acids were designed and not only demonstrated a significant vasoprotection of rat aortic rings challenged by the superoxide anion, but also showed strong antioxidant properties in vitro, while maintaining a low toxicity in various cell lines [16]. Bifunctional molecules are attracting significant attention in glioblastoma treatment due to their potential to overcome the complexities of this aggressive cancer. By targeting multiple biological processes simultaneously, these molecules aim to address drug resistance, tumor heterogeneity, and the immunosuppressive tumor microenvironment. Additionally, they offer the possibility of improved therapeutic efficacy with reduced toxicity. Examples include combinations of chemotherapy and immunotherapy, the dual inhibition of oncogenic pathways, and targeting both cancer cells and tumor vasculature. Antioxidants exhibiting anticancer activity include hydroxycinnamic acids [17], and more recently we developed flavonoid-based hybrids [18]. In addition to their antioxidant activity, these compounds may act through other mechanisms such as modulating the activity of certain enzymes and inhibiting cell proliferation. A synergistic action has been shown when metformin is used in combination with hydroxycinnamic acid derivatives. Indeed, a combined treatment with metformin and caffeic acid increased damage (apoptosis, inhibition of cell proliferation) compared to a treatment with metformin alone or caffeic acid alone on cancer cell lines [19].

Here, eight new hybrid molecules combining a guanidine moiety, as in metformin, and an antioxidant framework (hydroxycinnamic acid) were designed and synthesized. An additional hybrid based on resveratrol (3,5,4′-trihydroxystilbene), a potent antioxidant also known for its therapeutic properties against heart diseases, ischemic injuries, and cancer [20], was added to the above series.

The biological activity of the series was studied on several glioblastoma cell lines. An investigation of signaling pathways and redox modulation was carried out for the two most potent compounds. Our results suggest the efficiency of metformin–phenolic hybrid molecules as part of a new therapeutic strategy for glioblastoma.

## 2. Results and Discussion

### 2.1. Chemistry

**Figure 1:**
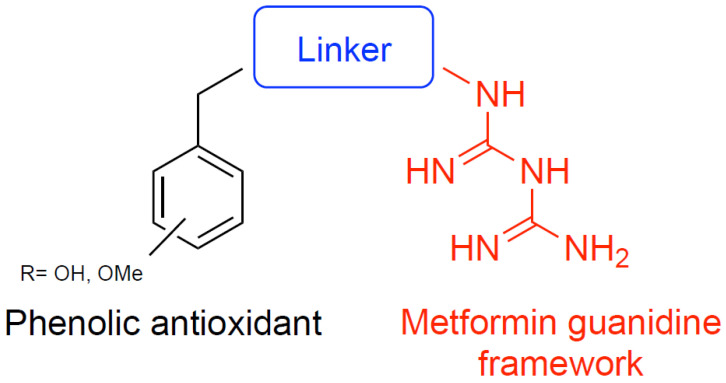
General structure of target metformin–phenolic hybrids.

In this study, eight of the novel conjugated hybrids, **5a**–**h**, were designed by connecting the metformin guanidine pharmacophore to a phenolic antioxidant moiety via a linear linker consisting of a *para*-phenylenediamine group attached to a short carbonyl-conjugated chain (Figure 1), both features known to provide an increased connectivity and stability in drug design.

**Scheme 1:**
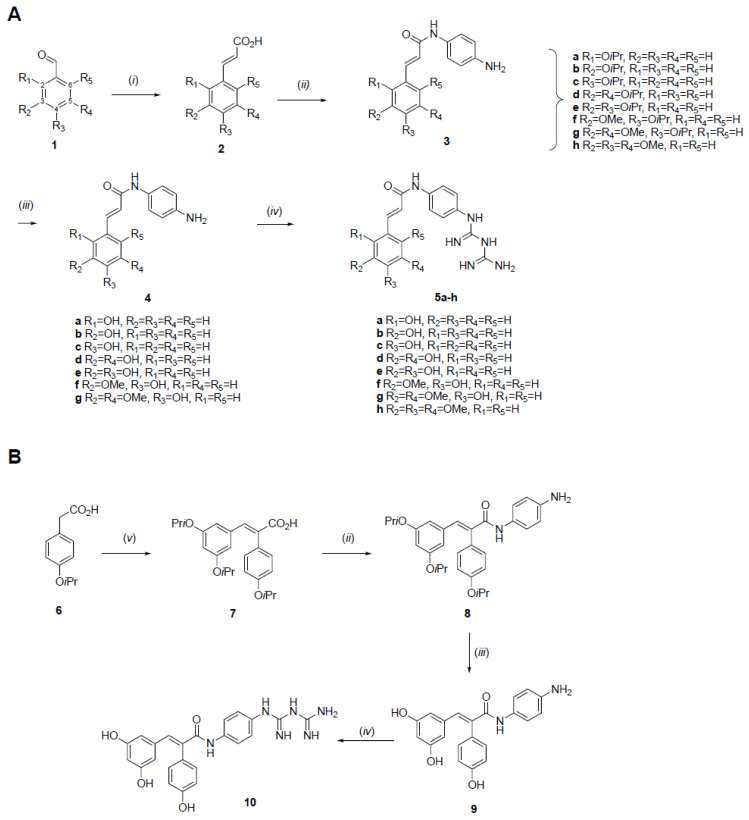
General synthesis of (**A**) metformin–phenolic acid hybrids **5a**–**h** and (**B**) metformin-resveratrol hybrid **10**. Reagents and conditions: (i) malonic acid, piperidine, acetic acid, 120 °C, 24 h, 59–100%; (ii) *para*-phenylenediamine, EDCI, HOBt, DIPEA, DMF, rt, 12 h, 86–100%; (iii) BCl_3_, DCM, –20 °C, 2 h, then 0 °C, 1 h, 47–100%; (iv) dicyandiamide, HCl cat., acetonitrile, microwave, 125 °C, 15 min, 34–95%; (v) **1d**, acetic anhydride, TEA, 100 °C, 1 d, 68%.

As shown in Scheme 1A, compound **5h** was synthesized in only three steps from 3,4,5-trimethoxybenzaldehyde, **3h**, while the other targets, **5a**–**g**, were obtained in a five-step sequence from the corresponding commercial hydroxybenzaldehydes, which first had their hydroxyl group(s) protected with 2-bromopropane to give the isopropoxy derivatives **1a**–**g** in good yields.

The following steps toward **5a**–**h** first included a Knoevenagel–Doebner condensation from piperidine, malonic acid, and the appropriate benzaldehyde, **1a**–**h**, to yield α,β-unsaturated carboxylic acids **2a**–**h**. The high ^1^H NMR ^3^J coupling values (15.6–16.1 Hz) observed in **2a**–**h** indicate that the reaction was stereoselective and only (*E*)-isomers were obtained. Second, a peptidic coupling between *para*-phenylenediamine and compounds **2a**–**h**, using EDCI and HOBt as coupling agents, and DIPEA directly led to compounds **3a**–**h** bearing the desired linker. Since attempts to introduce the guanidine moiety at this stage failed for compounds **3a**–**g**, they were first converted, in moderate to good yields, into their corresponding unprotected hydroxy analogs, **4a**–**g**, through a treatment with BCl_3_ in dichloromethane. The metformin moiety was finally introduced by condensing dicyandiamide directly on **3h** or on compounds **4a**–**g** under microwave irradiation to afford the desired hybrids **5a**–**h** in 31–66% overall yields.

Scheme 1B depicts the synthetic pathway towards metformin–resveratrol hybrid **10**, bearing a linker similar to compounds **5a**–**h**. The precursor **7** was prepared in two steps according to previously reported procedures [21,22] from commercial 2-(4-hydroxyphenyl)acetic acid, which was first treated with 2-bromopropane to give the hydroxy-protected compound **8** and then coupled with benzaldehyde **1d** to afford **7** at a 69% yield. From compound **7**, target hybrid **10** was conveniently prepared at a 41% overall yield in three steps via derivatives **8** and **9** using the same procedures described above for **5a**–**h**.

### 2.2. Cytotoxicity Studies

The cytotoxic properties of the hybrids and related compounds were first screened against glioblastoma cell lines U87 and U251 by measuring MTT cell viability. The innocuity of adding 0.1% DMSO to the corresponding media was checked prior to the experiments.

The hybrids exhibited a markedly enhanced cytotoxicity and could be classified into three main groups according to their IC_50_ values (Table 1, columns 2 and 3): group I: highly cytotoxic compounds (IC_50_ < 100 µM): **5a**, **5h**, and to a lesser extent **5b**; group II: intermediate cytotoxicity (100 < IC_50_ < 500 µM): **5c** and **5f**; group III: weakly cytotoxic or inactive compounds (IC_50_ > 500 µM): **5d**, **5e**, and **10**.

Notably, hydroxy-substituted compounds (**5a**–**c**, derived from coumaric acid) displayed a higher cytotoxicity than the corresponding methoxy derivatives (**5f**, derived from ferulic acid), in line with previous structure–activity trends observed in our group [23].

The reference temozolomide (TMZ), used in conventional therapy [24], exhibited an IC_50_ value of approximately 200 µM in U87 cells. In contrast, metformin displayed a much weaker cytotoxic activity, with IC_50_ values around 6 mM in U87 cells and 4 mM in U251 cells, values that are considerably higher than those achievable under conventional therapeutic conditions in vivo. These findings support the rational design of metformin–phenolic hybrids as a strategy to enhance the anticancer potential of metformin. The corresponding phenolic acids were also evaluated individually and exhibited no significant cytotoxic activity (IC_50_ > 500 µM) in U87 cells.

In contrast to our previous work on phenolic–nitrone hybrids [16], where the presence of nitrones reduced cytotoxicity compared to the free phenolic acids, hybridization with metformin showed the opposite trend. Here, metformin, although non-cytotoxic on its own, strongly enhances the cytotoxic activity of the hybrids. This highlights the significant contribution of metformin to the anticancer potential of these compounds.

To minimize potential biases associated with the cellular metabolic activity inherent to the MTT assay, cytotoxicity was further assessed through crystal violet staining, as described by Sliwka et al. [25]. The IC_50_ values obtained (**5a**: 29.4 µM, **5b**: 78.1 µM, and **5h**: 49.7 µM) were consistent with those determined through MTT, thereby confirming the reliability of our results.

Based on previously reported data [26], our results indicate that the introduction of the *para*-phenylenediamine linker did not impair the cytotoxic activity toward cancer cells, further supporting the relevance of our drug design.

The differences in cytotoxic potency among the hybrids may be partly explained by their calculated 1-octanol/water partition coefficients (AlogP). An intermediate lipophilicity appears to favor interactions with both the lipophilic (membrane) and aqueous (cytoplasmic) environments, leading to maximal cytotoxic effects as previously reported for antitumor phenolic derivatives [27,28]. Hybrids **5d** and **5e**, which bear two hydroxyl groups, displayed low AlogP values (1.32 and 1.34, respectively), consistent with their low cytotoxic activity. Conversely, compound **10**, characterized by a higher lipophilicity (AlogP = 3.12), also exhibited a weak activity, suggesting that excessive lipophilicity may impair intracellular distribution or molecular target interaction. Detailed AlogP values for all hybrids and their parent molecules are provided in the Supplementary Materials, Table S1.

Also, this outcome for compound **10** is consistent with the literature reporting that metformin in combination with resveratrol is less effective than resveratrol alone [29], supporting the weak activity observed.

The most cytotoxic hybrids identified in cell lines U87 and U251 were subsequently evaluated on glioblastoma stem-like cells (GBM6 and GBM9), and the corresponding IC_50_ values are summarized in Table 1 (columns 4 and 5). As cancer stem cells (CSCs) are known to drive tumor recurrence and therapy resistance, compounds capable of targeting them could provide significant therapeutic benefits. As expected, metformin displayed weak cytotoxicity in GBM6 and GBM9 cells, confirming the higher resistance of CSCs compared to U87 and U251 cells. In contrast, all tested hybrids (**5a**–**c** and **5h**) were 40–1000 times more cytotoxic than metformin in both CSC models, with **5a** and **5h** showing the strongest effects. Notably, their IC_50_ values were comparable to those in differentiated glioblastoma cells, suggesting a consistent cytotoxic mechanism.

To assess selectivity, metformin and its hybrids were also tested on human dermal fibroblasts (HDFs) (Table 1, column 6). Both metformin and most hybrids exhibited a lower cytotoxicity in HDFs than glioblastoma cells. Hybrids **5a** (IC_50_ > 500 µM) and **5h** (approximately 200 µM) displayed particularly favorable selectivity profiles, supporting their selection for further mechanistic investigations in glioblastoma models.

The differences in cytotoxicity observed among the hybrids prompted a deeper investigation into their mechanisms of action, particularly through the analysis of signaling pathways and redox status, to establish a link between the disruption of survival kinases and the induction of oxidative stress.

### 2.3. Signaling Pathway Study

Signaling pathways activated by metformin and the two most cytotoxic hybrids, **5a** and **5h**, were investigated using complementary approaches. First, a kinomic analysis was performed with PamGene technology to obtain a comprehensive overview of the mechanisms induced by the hybrid molecules. Then, Western blot analyses were carried out to compare the effects of metformin and its hybrids on the phosphorylation of selected kinases involved in these signaling pathways. In these further experiments, the concentrations used correspond to the IC_50_ values determined through MTT, ensuring direct biological relevance. Although higher than the plasma concentrations of metformin, they are comparable to those reported in preclinical development [15].

**Scheme 2:**
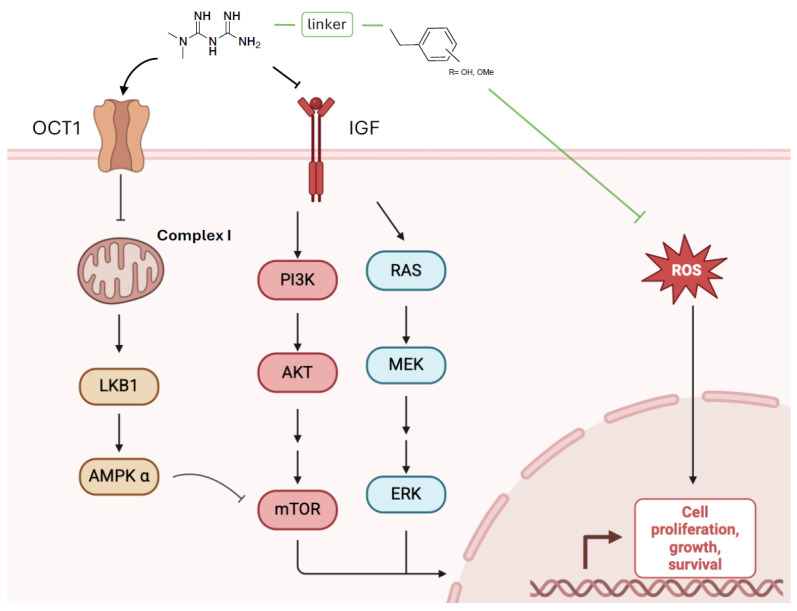
Principal signaling pathways of metformin on cancer cells and proposed multi-targeted pathways of hybrids.

The anticancer activity of metformin is associated with both direct and indirect effects of the drug (Scheme 2). Metformin accesses the intracellular space through organic cation transporter 1 (OCT1) and inhibits complex I of the mitochondrial electron transport chain, resulting in the activation of AMPK [15]. This activation mediates the direct insulin-independent effects of metformin, leading to reduced mTOR signaling and thus the inhibition of cell proliferation [30]. The indirect insulin-dependent effects of metformin are mediated by its ability to reduce insulin and insulin-like growth factor (IGF) levels, resulting in the inhibition of PI3K/AKT/mTOR and MEK1/2-ERK1/2 signaling pathways in cancer cells [31].

#### 2.3.1. Kinase Activity Assay

**Figure 2:**
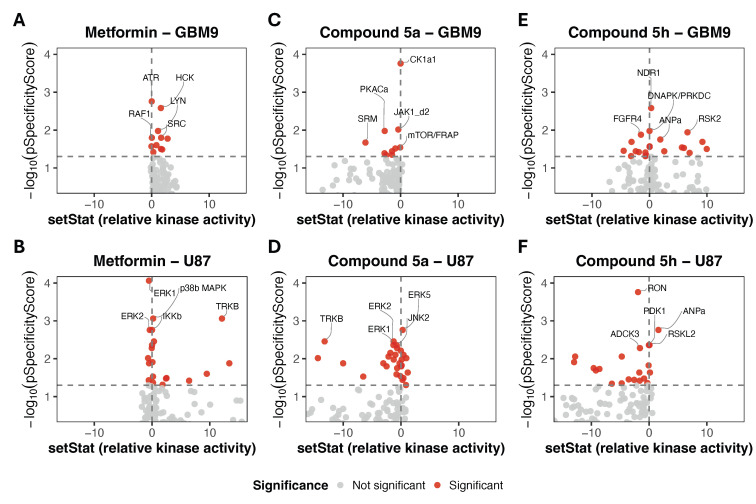
Effect of metformin (**A**,**B**), **5a** (**C**,**D**), and **5h** (**E**,**F**) on kinase signaling according to cell type. Volcano plots show relative changes in kinase activity in GBM9 stem-like cells (**A**,**C**,**E**) and U87 differentiated cells (**B**,**D**,**F**).

A kinomic analysis (PamGene technology) was performed to screen the kinase activities of metformin, **5a**, and **5h** (at IC_50_ for 4 h) in U87 and GBM9 cells, with results visualized as volcano plots (Figure 2A–F). These plots display relative changes in kinase activity (setStat) and statistical significance (pSpecificity), revealing a distinct reprogramming of kinase networks depending on the compound and cell context. The complete list of significantly activated and inhibited kinases for each treatment and cell type is provided in the Supplementary Materials, Tables S2–S4.

Metformin selectively activated SRC family kinases (HCK, LYN, YES1) and ATR in GBM9 cells (Figure 2A), consistent with the modulation of adhesion-related signaling and DNA damage response pathways. In U87 cells, it activated the TRK receptor family (NTRK1-3) and kinases associated with NF-κB signaling such as p38β (MAPK11), while inhibiting key components of MAPK signaling, including ERK1, ERK2, and IKKβ, as shown in Figure 2B. Other kinases modulated by metformin in U87 cells, including CDK2 and additional MAPK family members, are listed in Table S2. Overall, these results indicate that metformin primarily promotes metabolic stress adaptation rather than inducing apoptosis [32,33].

The treatment with hybrid **5a** in GBM9 cells (Figure 2C) displayed a broader impact, activating stress-related kinases CK1α1, CSNK2A1/2, IKKα, and CLK3 (Table S3) while inhibiting JAK1, JAK2, and RSK4 (Table S3), disrupting the MAPK–JAK proliferative cascade. In addition, **5a** inhibited several pro-survival and metabolic kinases, including SRM, PKACa, and mTOR/FRAP, which are associated with RTK-dependent signaling, cAMP/PKA activity, and mTOR-mediated metabolic control. A distinct kinase profile was observed in U87 cells (Figure 2D), where **5a** inhibited TRKB together with multiple members of the MAPK cascade (e.g., ERK1, ERK2, JNK2). Table S3 further highlighted the modulation of additional receptor tyrosine kinases (TRKA/C) and cell cycle-associated kinases (CDK2/7/L4/L5), indicating a widespread remodeling of signaling networks beyond the core MAPK components. These changes reflect a cell context-dependent rewiring of RTK–MAPK–NF-κB survival signaling in U87 cells, supporting the induction of apoptosis through NF-κB dysregulation and metabolic stress [34,35].

Concerning **5h** treatment in GBM9 cells (Figure 2E), the kinomic profile combined the inhibition of RTK, most notably FGFR4, along with FES, FRK, LTK, SRM, and TEC, as identified in Table S4, and the activation of kinases involved in checkpoint (DNA-PK), cell cycle regulation (RSK2, NDR1, CDK4), and signaling (ANPα, EPHA/B4), suggesting cytostatic resilience. Conversely, in U87 cells (Figure 2F), **5h** may contribute to a collapse of the RTK–PI3K–AKT–CDK axis, indicated by the inhibition of RON and MERTK, the suppression of metabolic and survival-associated kinases such as ADCK3 and PDK1, and the downregulation of TRK family kinases. Simultaneously, ANPα (NPR1) and RSKL2 were significantly activated, and an upregulation of the TAO family (TAO1–3) was observed (Table S4). This kinase signature is consistent with autophagic and apoptotic cell death responses [36,37].

In summary, metformin primarily affects the SRC family, RTK, and MAPK-related kinases, compatible with an adaptive cytostatic signaling rather than an extensive disruption of survival networks, whereas the hybrids **5a** and **5h** extend this modulation into multi-kinase inhibition. In GBM9 cells, their kinomic responses combine the inhibition of RTK-, MAPK-, JAK-, and metabolic kinases with the activation of stress and checkpoint regulators, resulting in signaling profiles compatible with enhanced growth control. In U87 cells, hybrids induce a more pronounced disruption of RTK–MAPK–PI3K signaling and an activation of stress-related kinases, supporting an engagement in cell death pathways. Overall, these findings suggest a shift from cytostatic adaptation under metformin treatment to cytotoxic reprogramming triggered by the hybrids, with a differential sensitivity between GBM9 and U87 cells. Although these changes in kinase activity correlate with cytotoxic phenotypes, genetic perturbation studies (siRNA or specific inhibitors) would be required to establish a direct causal relationship.

#### 2.3.2. Western Blot Analysis

To validate the kinomic hypotheses, time-resolved Western blots of AKT, AMPKα, and ERK1/2 were performed on U87 cells treated with metformin, **5a**, or **5h**. Protein quantification was carried out after 45 min, 4 h, and 24 h of treatment, and phosphorylated-to-total protein ratios were calculated to visualize kinase activation relative to the control. Cells maintained for 24 h under adhesion conditions in the presence of the vehicle [medium + 0.01% DMSO] were taken as a control. The innocuity of adding 0.01% DMSO to the medium was checked prior to the experiments and had no detectable effect on phosphorylation levels (see Supplementary Figure S1).

**Figure 3:**
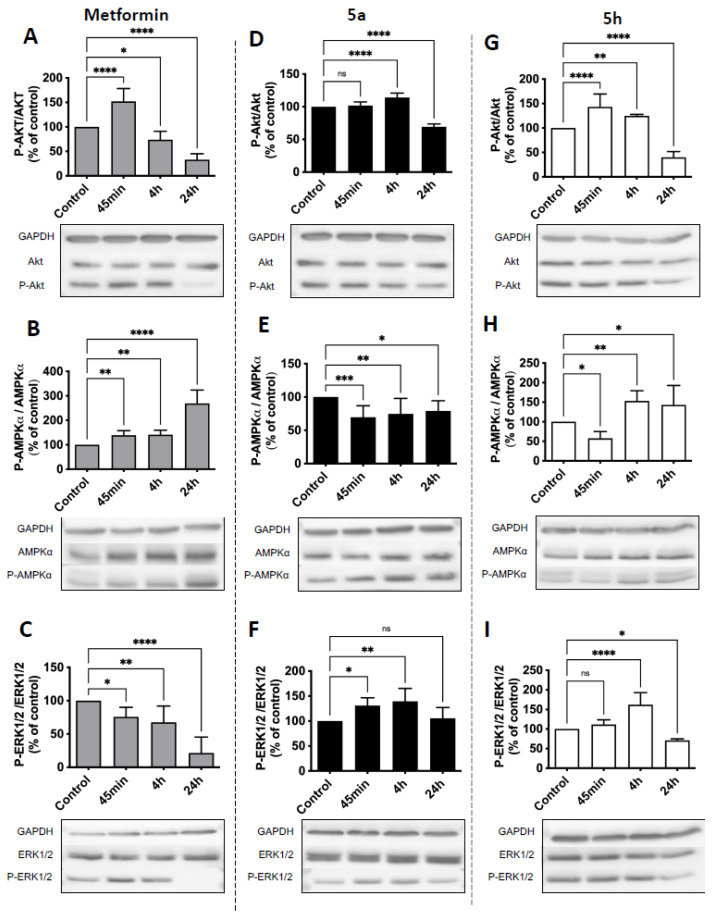
Effect of metformin (**A**–**C**), **5a** (**D**–**F**) and **5h** (**G**–**I**) on signaling pathways determined from P-AKT and AKT quantification (**upper** panel), P-AMPKα and AMPKα quantification (**middle** panel), and P-ERK1/2 and ERK1/2 quantification (**lower** panel). Human glioma U87 cells maintained in EMEM were exposed to metformin, **5a**, and **5h**, each incubated at their IC50 concentration (reported in Table 1) for 45 min, 4 h, or 24 h. Control: cells maintained 24 h under adherent conditions with vehicle [medium + 0.01% DMSO]. Data represent the mean ± SD of 3–8 independent experiments made in triplicate. Statistics: ns (not significant), * *p* < 0.05, ** *p* < 0.01, *** *p* < 0.001, and **** p < 0.00001 vs. control from one-way ANOVA followed by Dunnett’s multiple comparisons test.

Metformin’s effects were consistent with the signaling patterns identified by PamGene kinase profiling (e.g., metformin’s AMPK activation). As shown in Figure 3A, AKT phosphorylation progressively decreases, reaching about 30% of control levels after 24 h of treatment. Figure 3B highlights a strong activation of AMPKα, detectable as early as 45 min and reaching approximately 250% of the control at 24 h. In contrast, Figure 3C demonstrates a marked inhibition of ERK1/2 phosphorylation from 45 min onward, decreasing to 15% of the control after 24 h. The early AKT activation by metformin underscores dynamic signaling not captured in the 4 h PamGene snapshot.

Compound **5a**, tested at its IC_50_ concentration—approximately 200-fold lower than that of metformin—displayed a distinct signaling profile (Figure 3D–F). Figure 3D shows that AKT activity decreased after 4 h of treatment to around 70% of the control. Figure 3E indicates a slight reduction in the P-AMPKα/AMPKα ratio (approximately 90% of the control), suggesting a mild inhibition of AMPK activity. Meanwhile, Figure 3F reveals a transient increase in ERK1/2 phosphorylation at 45 min, peaking after 4 h, indicating a temporary activation of this pathway despite the inhibition detected through kinomic analysis, suggesting an adaptive stress response. The differences between **5a** containing the metformin moiety in the hybrid structure and metformin alone could explain the cytotoxicity of **5a,** likely associated with alternative signaling mechanisms beyond AKT, AMPK, and ERK1/2 modulation, as suggested by the kinomic analysis.

Compound **5h**, tested at a concentration 70-fold lower than that of metformin, exhibited a signaling profile more closely aligned with metformin (Figure 3G–I). As shown in Figure 3G, AKT phosphorylation decreased to roughly 40% of the control at 24 h. Figure 3H indicates AMPKα activation starting at 4 h and reaching about 150% of the control at 24 h. Finally, Figure 3I displays a decrease in ERK1/2 phosphorylation to approximately 70% of the control after 24 h. A set of original and uncropped pictures corresponding to Figure 3A–I is shown is Supplementary Materials, Figures S2–S10.

The integration of PamGene kinomic profiling and Western blot analyses provided complementary insights into the enhanced cytotoxicity of metformin–phenolic hybrids. While PamGene revealed broad kinase reprogramming (e.g., FGFR/MERTK inhibition by **5h**; Figure 2C,F), Western blots validated functional outcomes for AKT, AMPK, and ERK1/2, central to metformin’s mechanism [15,30]. Discrepancies (e.g., **5a**’s transient ERK1/2 activation; Figure 3F) reflect adaptive responses, emphasizing the need for time-resolved validation. Full kinomic datasets (Supplementary Tables S2–S4) offer a resource for future studies on less-characterized kinases (e.g., TAOK1-3).

### 2.4. Regulation of Redox State in U87 and GBM9 Cells

The impact of metformin and hybrids **5a** and **5h** on the regulation of ROS production in U87 and GBM9 cells was assessed at their IC_50_ concentration using three complementary standard probes: dichlorodihydrofluorescein diacetate (DCFDA) to monitor intracellular redox states, lucigenin to detect extracellular superoxide, and mitoSOX to measure mitochondria superoxide. Employing multiple probes allows a cellular compartment-specific assessment of ROS quantification, which is critical given the complex redox dynamics observed in cancer cells [38]. The control condition corresponds to the initial time point (t0) or to cells maintained for 4 h (MitoSOX) or 24 h (Lucigenin) under adhesion conditions in the presence of the solvent (vehicle).

**Figure 4:**
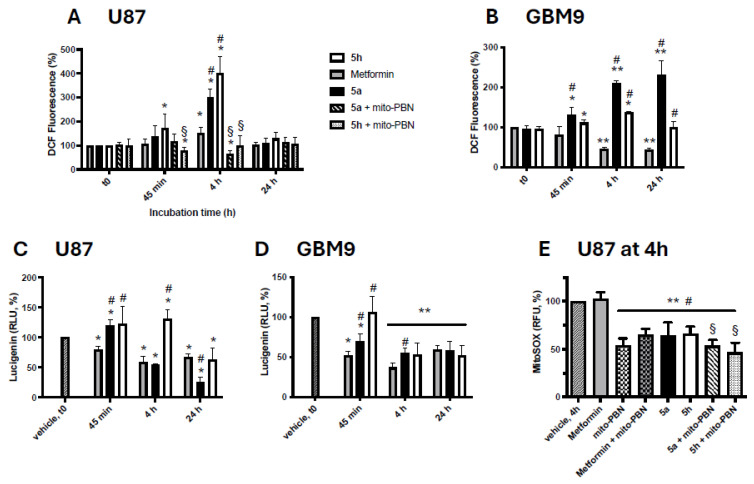
Effects of metformin, **5a**, **5h**, and the mitochondrial-targeted nitrone mito-PBN, alone or in combination, on intracellular redox state variations assayed using DCFDA in human U87 (**A**) and GBM9 cells (**B**); extracellular redox state variations in U87 (**C**) and GBM9 cells (**D**) assessed through lucigenin luminescence and mitochondrial ROS levels after 4 h of treatment in U87 cells, assayed using MitoSOX reagent (**E**). Metformin, **5a**, and **5h** were given at their IC_50_ concentrations (see Table 1) for 45 min, 4 h, or 24 h. Nitrone mito-PBN (10 μM) pretreatment was performed for 1 h, followed by medium removal and a 4 h incubation with test compounds. The control condition corresponds to initial time point (t0) or cells maintained for 4 h (MitoSOX) or 24 h (Lucigenin) under adhesion conditions in the presence of the solvent (vehicle). Values represent the mean ± SD of 3–6 independent experiments made in triplicate. Statistical analysis was conducted using one-way ANOVA followed by a posteriori Dunnett’s multiple comparisons test or two-tailed unpaired *t* test: * *p* < 0.05 and ** *p* < 0.01 vs. corresponding control; # *p* < 0.01 compared to metformin at the same time of treatment; § *p* < 0.05 compared to the same treatment without mito-PBN.

In U87 cells, metformin appeared to induce only a modest increase in intracellular oxidative status after 4 h (Figure 4A), consistent with previous reports [39] in which redox elevations were observed at substantially higher concentrations. This study also emphasizes that oxidative responses to metformin are cell line dependent. The reinforced antioxidant systems characteristic of glioblastoma stem-like cells [40] may explain the measured decrease toward a more reduced intracellular environment in GBM9 (Figure 4B). In contrast, hybrids **5a** and **5h** triggered a substantial intracellular oxidative stress surge in both cell lines (Figure 4A,B). In U87 cells, the redox signal peaked at 4 h, reaching approximately 300% of the control for hybrid **5a** and up to 400% for hybrid **5h** (Figure 4A), indicating a strong cytosolic oxidative burden. GBM9 cells also marked a clear intracellular oxidative status at the same time, with levels reaching approximately 210% of the control with hybrid **5a** and 138% with hybrid **5h** (Figure 4B). Such a rapid intracellular ROS accumulation suggests that the hybrids exceed the antioxidant buffering capacity of cells and impose a redox imbalance potentially incompatible with long-term survival. Indeed, the accumulation of intracellular oxidative stress is well established as a trigger for apoptotic pathways when cellular redox buffering capacity is exceeded. This mechanism has been demonstrated in several glioblastoma studies. For example, β-Elemene induces a dose-dependent ROS accumulation, leading to mitochondrial dysfunction and apoptotic death in U87 cells [41]. Auranofin triggers ROS-dependent apoptosis in GBM and GBM stem-like cells through the inhibition of thioredoxin reductase [40]. Taken together, these studies highlight that a sufficiently strong oxidative shift could help overcome the robust antioxidant defenses of glioblastoma cells. In this context, the rapid and intense change in intracellular redox state induced by hybrids **5a** and **5h** at relatively low concentrations may indicate a potent pro-oxidant cytotoxic mechanism compared with classical redox-modulating agents.

The values obtained with lucigenin revealed that metformin significantly decreased extracellular oxidative activity in both U87 and GBM9 cells, reaching approximately 50–60% of control levels after 24 h (Figure 4C,D). Extracellular ROS release may originate from NADPH oxidase (NOX) activity, which is known to contribute significantly to ROS production and redox imbalance in glioblastoma [42], supporting the concept that the modulation of NOX activity could influence extracellular oxidative signals and cell survival. Hybrids **5a** and **5h** also appeared to exert extracellular antioxidant effects, although with different magnitudes. Typically, in U87 cells (Figure 4C), hybrid **5a** reduced extracellular oxidative activity to nearly 25% of the control after 24 h, suggesting a strong antioxidant action, whereas hybrid **5h** showed a more moderate reduction, possibly related to its stronger intracellular pro-oxidant activity. In GBM9 cells (Figure 4D), both hybrids decreased the extracellular redox signal to around 50% of the control, indicating that their ability to modulate the extracellular oxidative environment could be preserved even in a stem-like, redox-resistant cellular context. The decrease in extracellular redox activity induced by the hybrids may reflect the downregulation of NOX activity and enhanced extracellular antioxidant defenses, suggesting a cell-protective redox modulation. The transient increase in the extracellular oxidative environment observed at 45 min and 4 h of treatment in both cell lines may be attributed to the partial release of intracellular ROS. Since extracellular ROS contribute to tumor–microenvironment interactions and the maintenance of stemness [42], their inhibition may weaken pro-tumorigenic signaling even in redox-resistant GBM9 cells.

The reagent MitoSOX is used to specifically monitor mitochondrial superoxide levels [43]. Figure 4E shows that metformin (6 mM) did not significantly alter mitochondrial superoxide production in U87 cells, while hybrids **5a** and **5h** reduced mitochondrial ROS to approximately 65% of the control after 4 h when used at their IC_50_ concentration. Although mitochondrial ROS were not evaluated in GBM9 cells with MitoSOX, the parallel intracellular and extracellular ROS patterns observed across both cell lines may suggest that hybrids potentially exert a similar mitochondrial antioxidant effect in GBM9. Hybrids **5a** and **5h** appeared to shift the oxidative imbalance predominantly toward the cytosolic compartment, conceivably creating an intracellular environment incompatible with survival while simultaneously reducing mitochondrial ROS. A similar mitochondrial antioxidant effect of flavonoid–phenolic acid hybrids has been reported in HT22 cells, where ROS reduction was associated with the preservation of mitochondrial function [44].

To investigate the origin of intracellular ROS, MitoSOX staining was assayed in the presence of the mitochondria-targeted nitrone Mito-PBN, given as a pretreatment before test compound incubation [45]. It was observed that Mito-PBN substantially reduced the MitoSOX signal in cells treated with metformin, hybrid **5a**, or **5h** (Figure 4E), suggesting that oxidant species may originate primarily from mitochondria. This aligns with previous findings using Mito-TEMPO [46], supporting the idea that the present hybrids could modulate mitochondrial redox homeostasis. Consistently, DCFDA staining in the presence of Mito-PBN evidenced a marked reduction in intracellular ROS (Figure 4A), supporting the possibility that the hybrid compounds induce predominantly oxidant species of mitochondrial origin which can diffuse and largely contribute to cytosolic oxidative stress.

Altogether, the analysis of extracellular, intracellular, and mitochondrial oxidative stress parameters indicated that hybrids **5a** and **5h** could induce a compartment-dependent redox remodeling. While metformin primarily appeared to act as an extracellular antioxidant with a limited intracellular impact, the hybrids seem to trigger a strong intracellular pro-oxidant activity and may modulate mitochondrial ROS production and exhibit extracellular antioxidant effects. This redox profile is consistent with the observed kinase reprogramming, including the inhibition of key survival pathways such as RTK–MAPK–PI3K–AKT and the activation of stress-response kinases, which contribute to cellular antioxidant defenses and sensitize cells to ROS accumulation. In contrast, metformin is observed to activate AMPK and to downregulate ERK and AKT, promoting cytostatic metabolic adaptation. In both U87 and GBM9 cells, the hybrids’ rapid intracellular oxidative stress accumulation, together with the suppression of key pro-survival signaling nodes, likely supports a mechanistic basis for their enhanced cytotoxicity. These findings are consistent with the concept that surpassing the intracellular redox state tolerance threshold can initiate apoptosis in glioblastoma cells and particularly in stem-like populations [47]. Overall, our data support a model in which metformin induces cytostatic metabolic adaptation, whereas the metformin–phenolic hybrids may exert their antitumor activity through a synergistic mechanism involving intracellular oxidative stress, the perturbation of redox buffering, and the inhibition of key signaling pathways, thereby increasing susceptibility to cell death pathways in a cell context-dependent manner.

### 2.5. Antioxidant Capacities

**Figure 5:**
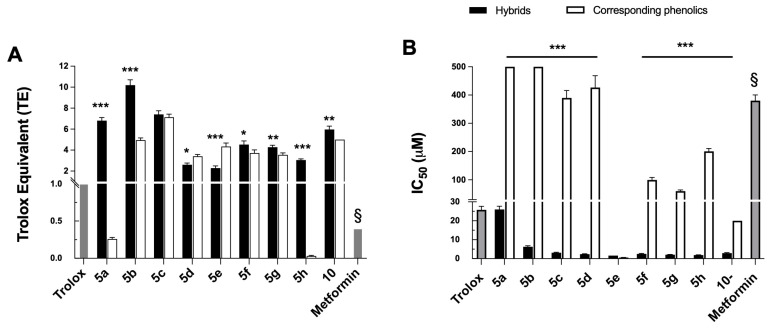
In vitro antioxidant properties of hybrids and corresponding phenolics through (**A**) ORAC ^a^ and (**B**) superoxide quenching assay ^b^. ^a^ Results are expressed as Trolox Equivalent (TE; ORAC = 1 for Trolox). ^b^ Superoxide generator was allopurinol–xanthine oxidase system, and scavenging activity was determined by assaying lucigenin chemiluminescence. Data are means ± SD of 3–6 independent experiments made in triplicate. Statistics: one-way ANOVA followed by Dunnett’s multiple comparisons test. * *p* < 0.05, ** *p* < 0.01, and *** *p* < 0.001 for hybrids vs. corresponding phenolic; § *p* <0.001 for each hybrid vs. metformin.

The antioxidant properties of the metformin and hybrids were evaluated using three standard assays, namely DPPH radical scavenging, the ORAC assay, and the quenching of O_2_^•−^ [48,49]. The results are shown in Table 2 and Figure 5 and are compared with the corresponding phenolic acids and Trolox as standard antioxidants.

#### 2.5.1. DPPH Reducing Capacity of Hybrids

The antioxidant capacity of hybrid molecules and their corresponding phenolic acids was evaluated at 3 min and 30 min using the DPPH assay (Table 2). All compounds showed higher EC_50_ values at 3 min than at 30 min, indicating slower reaction kinetics with the DPPH radical in contrast to Trolox (35.0 ± 2.0 μM at 3 min). As some studies have demonstrated a correlation between phenolics’ reactivity towards free radicals and cytotoxicity [50], the slower kinetics of metformin hybrids could be advantageous in the prevention of oxidative stress.

The hybridization of hydroxycinnamic acids with a guanidine moiety markedly enhanced their antioxidant properties, with hybrids being 1.3 to >4 times more potent than the parent phenolics, except for caffeic acid (**5e** vs. caffeic acid). Caffeic acid has a particular and specific structure with a catechol group responsible for its strong antioxidant properties [16,51]. Unfortunately, its hybridization with metformin reduced its reactivity against DPPH by 3 times. Surprisingly, hybrid **5d** did not exert an antioxidant effect at 200 µM, highlighting that the antioxidant capacity depends not only on the number of hydroxyl groups (**5a**, **5b**, **5c** vs. **5d**) but also on the substitution pattern (**5d** vs. **5e**). The introduction of one or two methoxy substituents in addition to the *para*-hydroxy (**5f**, **5g** vs. **5e**) slightly improved activity, with **5g** being the most active at 30 min across the whole series. However, adding a third methoxy group resulted in a slight decrease in the DPPH scavenging efficiency (**5h** vs. **5e**). Finally, the resveratrol-based hybrid **10** displayed a low activity, consistent with the moderate DPPH reduction capacity of the resveratrol itself as reported by Lee et al. [52]. Although they demonstrated that the derivatization of resveratrol can improve radical scavenging activity, hybridization with metformin showed no benefit. Overall, hybrid molecules **5a**, **5b**, **5c**, and **5g** exhibited the most potent DPPH radical scavenging properties, with EC_50_ values after 30 min of reaction below 50 µM.

#### 2.5.2. ORAC Assay

The ORAC test determines the antioxidant activity of molecules against peroxyl radicals ROO^•^ by monitoring the fluorescence decay of fluorescein, which is quenched in the presence of ROS. Overall, the hybrids exhibited stronger antioxidant properties compared to Trolox (Figure 5A). Hybridization with the guanidine moiety markedly enhanced the peroxyl radical scavenging capacity, particularly for compounds **5a** and **5h**, whereas hybrids **5c**, **5d**, **5f**, and **5g** displayed activities comparable to their parent molecules. Although metformin itself has no antioxidant properties, its incorporation into hydroxycinnamic acid structures significantly improved their ability to trap peroxyl radicals. These findings are consistent with the lucigenin measurements, both assays indicating that hybrids **5a** and **5h** possess strong a antioxidant activity.

#### 2.5.3. Superoxide Quenching Activity

The superoxide O_2_^•−^ inhibition properties of hybrid molecules and their corresponding phenolic acids were evaluated over the 0.1–500 µM range using the allopurinol–xanthine oxidase system as a free radical generator and lucigenin chemiluminescence as the detector. Hybrids exhibited IC_50_ values between 1.6 and 26 µM, all lower than that of Trolox (Figure 5B), indicating a better superoxide quenching capacity. The number and nature of electron-donating substituents appeared to influence the activity [16,53], as compounds **5d**–**h**, having two or three electron-donating groups (hydroxy and methoxy), were more effective, whereas hybrid **5a**, bearing only one hydroxyl substituent, showed the weakest activity in the series. Taken together, these results are consistent with the MitoSOX experiment, pointing out that hybrids **5a** and **5h** may effectively modulate mitochondrial superoxide production.

In conclusion, metformin showed no significant antioxidant activity in vitro, which is noteworthy given its antioxidant properties observed in vivo. However, the hybridization of the guanidine scaffold with phenolic compounds markedly enhanced their intrinsic antioxidant capacity compared to the parent phenolic molecules. As hybrids also induced a pronounced intracellular oxidative stress, this apparent dual pro- and antioxidant behavior suggests a compartment-dependent redox modulation, providing a therapeutic advantage by selectively affecting tumor cells while limiting pro-tumorigenic oxidative signaling. Taken together, these findings reinforce the hypothesis that hybrids **5a** and **5h** act as redox-modulating agents rather than simple antioxidants and may represent promising candidates for cancer prevention by limiting oxidative stress-induced cellular damage and protecting cellular redox homeostasis.

## 3. Materials and Methods

### 3.1. Chemistry

Reactions were routinely monitored through TLC on Merck Kieselgel 60 F254 precoated silica gel plates (Sigma-Aldrich, Saint Quentin Fallavier, France), and the spots were visualized under UV light. Microwave-assisted reactions were carried out using a Monowave 400 reactor (Anton Paar, Les Ulis, France), equipped with a ruby-thermometer and set at a microwave power of 400 W. NMR spectra were recorded on Bruker (Bruker France SAS, Wissembourg, France) AVL300 or Bruker AVL400 spectrometers at 300 or 400 M for ^1^H and 75.5 MHz or 100.6 MHz for ^13^C, respectively. Deuterated solvents were from Eurisotop (Saint-Aubin, France). Chemical shifts were expressed as *δ* (ppm) relative to TMS. Melting points were determined on a M-560 melting point apparatus (Büchi SARL, Villebon-sur-Yvette, France). High-resolution mass spectrometry (HRMS) in electron spray ionization (ESI) was performed at the Spectropole (Analytical Laboratory) at Campus St. Jérôme (Marseille, France) on a Waters SYNAPT G2 HDMS instrument (Waters Corporation, Milford, MA, USA). Purity of final compounds was determined using High-Performance Liquid Chromatography (HPLC) with an Agilent System (Agilent Technologies France, Les Ulis, France) consisting of a 1260 Infinity II interface module combined with a High Sensibility Max-Light photodiode array detector controlled by the Agilent OpenLab Chromatography manager software. Reverse phase HPLC was carried out at 35 °C on a Nucleodur C18 HTech column (4.6 mm × 250 mm, 5 μm; Macherey Nagel SAS, Gutenberg, France). The mobile phase for HPLC was freshly degassed H_2_O:acetonitrile (6:4 *v*/*v*), using 9 min of isocratic mode at a flow rate of 0.8 mL·min^−1^. Samples were diluted in H_2_O (1:1 *v*/*v*) before vortexing, and 10 μL of the mixture was then injected into the HPLC system. Absorbance was measured at 320 nm, and quantification was performed by comparing the total peak area with the peak area of each compound using Agilent OpenLab Analysis (V2.4) software. Starting materials, reagents, and solvents were analytical grade from commercial sources (Sigma-Aldrich, Saint Quentin Fallavier, France; TCI, Zwijndrecht, Belgium; Acros Organics, Fisher Scientific SAS, Illkirch, France) and were used without further purification. Compounds **1a**–**g**, **6**, and **7** are already described, and the analytical and/or NMR data obtained here are in agreement with the literature. Their detailed synthesis procedures and characterization are given in the Supplementary Materials.

#### 3.1.1. General Procedure for the Synthesis of **3a**–**h**

To the corresponding carboxylic acid derivative **2a**–**h**, dissolved in DMF (0.5 M, final concentration), was successively added phenylenediamine (1 eq), EDCI (1.2 eq), HOBt (1.2 eq), and DIPEA (1.5 eq), and the mixture was stirred overnight at room temperature. Dichloromethane (same DMF volume) was added, and the organic phase was washed with 5% aqueous NaOH (three times) and with water (three times). The organic layer was dried over MgSO_4_, filtered off, and concentrated to afford the desired compound, used without further purification.

(*E*)-*N*-(4-Aminophenyl)-3-(2-isoproproxyphenyl)acrylamide (**3a**): Title compound was obtained from **2a** (600 mg, 2.91 mmol), 4-phenylenediamine (315 mg, 2.91 mmol), EDCI (670 mg, 3.50 mmol), HOBt (472 mg, 3.50 mmol), and DIPEA (760 µL, 4.37 mmol). Brown pale powder (748 mg, quant. yield), mp 224 °C. ^1^H NMR (300 MHz, DMSO-*d*_6_) *δ* 9.77 (s, 1H, NH), 7.76 (d, 1H, *J* = 15.9 Hz, Ar-C*H*=CH), 7.55 (dd, 1H, *J* = 7.8 and 1.5 Hz, H-6), 7.36 (d, 2H, *J* = 8.3 Hz, H-2a), 7.33 (m, 1H, H-4), 7.10 (d, 1H, *J* = 8.1 Hz, H-3), 6.98 (t, 1H, *J* = 7.7 Hz, H-5), 6.76 (d, 1H, *J* = 15.9 Hz, Ar-CH=C*H*), 6.53 (d, 2H, *J* = 8.3 Hz, H-3a), 4.91 (bs, 2H, NH_2_), 4.70 (sept, 1H, *J* = 6.2 Hz, C*H*(CH_3_)_2_), 1.33 (d, 6H, *J* = 6.2 Hz, CH(C*H*_3_)_2_); ^13^C NMR (75 MHz, DMSO-*d*_6_) *δ* 162.8 (C=O), 155.7 (C-2), 144.7 (C-4a), 133.9 (Ar-*C*H=CH), 130.7 (C-4), 128.3 (C-1a), 127.5 (C-6), 124.2 (C-1), 122.4 (Ar-CH=*C*H), 120.7 (C-2a), 120.4 (C-5), 114.1 (C-3), 113.7 (C-3a), 70.0 (*C*H(CH_3_)_2_), 21.7 (CH(*C*H_3_)_2_). HRMS calc. for C_18_H_21_N_2_O_2_^+^ [M + H]^+^ 297.1598, found 297.1597.

(*E*)-*N*-(4-Aminophenyl)-3-(3-isoproproxyphenyl)acrylamide (**3b**): Title compound was obtained from **2b** (800 mg, 3.88 mmol), 4-phenylenediamine (419 mg, 3.88 mmol), EDCI (894 mg, 4.66 mmol), HOBt (629 mg, 4.66 mmol), and DIPEA (1010 µL, 5.82 mmol). Brown pale powder (1.14 g, quant. yield), mp 129–129.4 °C. ^1^H NMR (300 MHz, DMSO-*d*_6_) *δ* 9.75 (s, 1H, NH), 7.47 (d, 1H, *J* = 15.9 Hz, Ar-C*H*=CH), 7.36 (d, 2H, *J* = 8.7 Hz, H-2a), 7.31 (d, 1H, *J* = 8.1 Hz, H-5), 7.14 (d, 1H, *J* = 8.4 Hz, H-6), 7.12 (d, 1H, *J* = 2.4 Hz, H-2), 6.93 (dd, 1H, *J* = 8.1 and 2.4 Hz, H-4), 6.78 (d, 1H, *J* = 15.9 Hz, Ar-CH=C*H*), 6.55 (d, 2H, *J* = 8.7 Hz, H-3a), 4.88 (s, 2H, NH_2_), 4.65 (sept, 1H, *J* = 6.0 Hz, C*H*(CH_3_)_2_), 1.29 (d, 6H, *J* = 6.0 Hz, CH(C*H*_3_)_2_); ^13^C NMR (75 MHz, DMSO-*d*_6_) *δ* 163.1 (C=O), 158.3 (C-3), 145.5 (C-4a), 139.3 (Ar-*C*H=CH), 137.0(C-1), 130.5 (C-5), 129.0 (C-1a), 123.6 (Ar-CH=*C*H), 121.3 (C-2a), 120.1 (C-6), 117.3 (C-4), 115.0 (C-2), 114.4 (C-3a), 69.8 (*C*H(CH_3_)_2_), 22.4 (CH(*C*H_3_)_2_). HRMS calc. for C_18_H_21_N_2_O_2_^+^ [M + H]^+^ 297.1598, found 297.1597.

(*E*)-*N*-(4-Aminophenyl)-3-(4-isoproproxyphenyl)acrylamide (**3c**): Title compound was obtained from **2c** (800 mg, 3.88 mmol), 4-phenylenediamine (419 mg, 3.88 mmol), EDCI (894 mg, 4.66 mmol), HOBt (629 mg, 4.66 mmol), and DIPEA (1010 µL, 5.82 mmol). Brown pale powder (1.14 g, quant.), mp 160.3–161.2 °C. ^1^H NMR (300 MHz, DMSO-*d*_6_) *δ* 9.66 (s, 1H, NH), 7.49 (d, 2H, *J* = 8.7 Hz, H-2), 7.42 (d, 1H, *J* = 15.5 Hz, Ar-C*H*=CH), 7.32 (d, 1H, *J* = 8.7 Hz, H-2a), 6.93 (d, 2H, *J* = 8.7 Hz, H-3), 6.60 (d, 1H, *J* = 15.5 Hz, Ar-CH=C*H*), 6.52 (d, 2H, *J* = 8.7 Hz, H-3a), 4.84 (s, 2H, NH_2_), 4.64 (sept, 1H, *J* = 6.1 Hz, C*H*(CH_3_)_2_), 1.26 (d, 6H, *J* = 6.1 Hz, CH(C*H*_3_)_2_); ^13^C NMR (75 MHz, DMSO-*d*_6_) *δ* 163.4 (C=O), 159.2 (C-4), 145.3 (C-4a), 139.1 (Ar-*C*H=CH), 129.6 (C-2), 129.2 (C-1a), 127.7 (C-1), 121.2 (C-2a), 120.6 (Ar-CH=*C*H), 116.4 (C-3), 114.4 (C-3a), 69.8 (*C*H(CH_3_)_2_), 22.3 (CH(*C*H_3_)_2_). HRMS calc. for C_18_H_21_N_2_O_2_^+^ [M + H]^+^ 297.1598, found 297.1599.

(*E*)-*N*-(4-Aminophenyl)-3-(3,5diisoproproxyphenyl)acrylamide (**3d**): Title compound was obtained from **2d** (500 mg, 1.89 mmol), 4-phenylenediamine (205 mg, 1.89 mmol), EDCI (436 mg, 2.23 mmol), HOBt (307 mg, 2.23 mmol), and DIPEA (495 µL, 2.84 mmol). Brown gum (669 mg, quant. yield). ^1^H NMR (400 MHz, DMSO-*d*_6_) *δ* 9.75 (s, 1H, NH), 7.39 (d, 1H, *J* = 15.8 Hz, Ar-C*H*=CH), 7.34 (d, 2H, *J* = 8.8 Hz, H-2a), 6.73 (d, 1H, *J* = 15.8 Hz, Ar-CH=C*H*), 6.69 (d, 2H, *J* = 2.2 Hz, H-2), 6.52 (d, 2H, *J* = 8.8 Hz, H-3a), 6.45 (t, 1H, *J* = 2.2 Hz, H-4), 4.92 (s, 2H, NH_2_), 4.63 (sept, 2H, *J* = 6.2 Hz, 2 × C*H*(CH_3_)_2_), 1.27 (d, 12H, *J* = 6.2 Hz, 2 × CH(C*H*_3_)_2_); ^13^C NMR (100.6 MHz, DMSO-*d*_6_) *δ* 162.5 (C=O), 159.0 (C-3), 144.9 (Ar-*C*H=CH), 139.0 (C-4a), 137.0 (C-1), 128.4 (C-1a), 123.1 (Ar-CH=*C*H), 120.8 (C-2a), 113.8 (C-3a), 106.9 (C-2), 104.6 (C-4), 69.4 (2 × *C*H(CH_3_)_2_), 21.9 (2 × CH(*C*H_3_)_2_). HRMS calc. for C_21_H_27_N_2_O_3_^+^ [M + H]^+^ 355.2016, found 355.2018.

(*E*)-*N*-(4-Aminophenyl)-3-(3,4-diisopropoxyphenyl)acrylamide (**3e**): Title compound was obtained from **2e** (6.9 g, 26.1 mmol), 4-phenylenediamine (2.8 g, 26.1 mmol), EDCI (6.0 g, 31.3 mmol), HOBt (4.2 g, 31.3 mmol), and DIPEA (6.8 mL, 39.2 mmol). Orange powder (9.7 g, quant.), mp 111 °C. ^1^H NMR (300 MHz, DMSO-*d*_6_) *δ* 9.73 (bs, 1H, NH), 7.42 (d, 1H, *J* = 15.6 Hz, Ar-C*H*=CH), 7.36 (d, 2H, *J* = 8.6 Hz, H-2a), 7.18 (d, 1H, *J* = 1.7 Hz, H-2), 7.14 (dd, 1H, *J* = 8.3 Hz and *J* = 1.5 Hz, H-6), 6.98 (d, 1H, *J* = 8.2 Hz, H-5), 6.64 (d, 1H, *J* = 15.6 Hz, Ar-CH=C*H*), 6.54 (d, 2H, *J* = 8.6 Hz, H-3a), 4.89 (bs, 2H, NH_2_), 4.59–4.47 (m, 2H, *J* = 6.1 Hz, 2 × C*H*(CH_3_)_2_), 1.28 (d, 6H, *J* = 6.1 Hz, CH(C*H*_3_)_2_), 1.27 (d, 6H, *J* = 6.1 Hz, CH(C*H*_3_)_2_); ^13^C NMR (75 MHz, DMSO-*d*_6_) *δ* 163.3 (C=O), 150.3 (C-4), 148.6 (C-3), 145.3 (C-4a), 139.3 (Ar-*C*H=CH), 129.2 (C-1a), 128.7 (C-1), 122.3 (C-6), 121.1 (C-2a), 121.1 (Ar-CH=*C*H), 116.9 (C-5), 116.6 (C-2), 114.3 (C-3a), 71.8 (*C*H(CH_3_)_2_), 71.2 (*C*H(CH_3_)_2_), 22.5 (CH(*C*H_3_)_2_), 22.4 (CH(*C*H_3_)_2_). HRMS calc. for C_21_H_27_N_2_O_3_^+^ [M + H]^+^ 355.2016, found 355.2016.

(*E*)-*N*-(4-Aminophenyl)-3-(4-isopropoxy-3-methoxyphenyl)acrylamide (**3f**): Title compound was obtained from **2f** (2.7 g, 11.5 mmol), 4-phenylenediamine (1.3 g, 11.5 mmol), EDCI (2.6 g, 14.0 mmol), HOBt (1.8 g, 14.0 mmol), and DIPEA (3.0 mL, 17.0 mmol). Orange powder (3.7 g, 99%), mp 102 °C. ^1^H NMR (300 MHz, DMSO-*d*_6_) *δ* 9.72 (bs, 1H, NH), 7.43 (d, 1H, *J* = 15.6 Hz, Ar-C*H*=CH), 7.35 (d, 2H, *J* = 8.8 Hz, H-2a), 7.18 (d, 1H, *J* = 1.7 Hz, H-2), 7.11 (dd, 1H, *J* = 8.4 Hz and *J* = 1.7 Hz, H-6), 6.99 (d, 1H, *J* = 8.4 Hz, H-5), 6.64 (d, 1H, *J* = 15.6 Hz, Ar-CH=C*H*), 6.53 (d, 2H, *J* = 8.8 Hz, H-3a), 4.89 (bs, 2H, NH_2_), 4.60 (sept, 1H, *J* = 6.1 Hz, C*H*(CH_3_)_2_), 3.80 (s, 3H, OCH_3_), 1.26 (d, 6H, *J* = 6.1 Hz, CH(C*H*_3_)_2_); ^13^C NMR (75 MHz, DMSO-*d*_6_) *δ* 163.4 (C=O), 150.3 (C-4), 148.6 (C-3), 145.3 (C-4a), 139.4 (C-1), 129.2 (C-1a), 128.3 (Ar-*C*H=CH), 121.8 (C-6), 121.1 (C-2a), 120.9 (Ar-CH=*C*H), 115.2 (C-5), 114.3 (C-3a), 111.0 (C-2), 70.7 (*C*H(CH_3_)_2_), 55.9 (OCH_3_), 22.3f (CH(*C*H_3_)_2_). HRMS calc. for C_19_H_23_N_2_O_3_^+^ [M + H]^+^ 327.1703, found 327.1703.

(*E*)-*N*-(4-Aminophenyl)-3-(4-isopropoxy-3,5-dimethoxyphenyl)acrylamide (**3g**): Title compound was obtained from **2g** (1.6 g, 6.01 mmol), 4-phenylenediamine (0.65 g, 6.01 mmol), EDCI (1.4 g, 7.21 mmol), HOBt (0.98 g, 7.21 mmol), and DIPEA (1.6 mL, 9.02 mmol). Orange powder (1.9 g, 86%), mp 172 °C. ^1^H NMR (300 MHz, DMSO-*d*_6_) *δ* 9.78 (bs, 1H, NH), 7.46 (d, 1H, *J* = 15.6 Hz, Ar-C*H*=CH), 7.37 (d, 2H, *J* = 8.6 Hz, H-2a), 6.92 (s, 2H, H-2), 6.72 (d, 1H, *J* = 15.6 Hz, Ar-CH=C*H*), 6.54 (d, 2H, *J* = 8.6 Hz, H-3a), 4.90 (bs, 2H, NH_2_), 4.31 (sept, 1H, *J* = 6.2 Hz, C*H*(CH_3_)_2_), 3.81 (s, 6H, 2 × OCH_3_), 1.18 (d, 6H, *J* = 6.1 Hz, CH(C*H*_3_)_2_); ^13^C NMR (75 MHz, DMSO-*d*_6_) *δ* 163.1 (C=O), 154.1 (C-3), 145.3 (C-4), 139.6 (Ar-*C*H=CH), 137.1 (C-4a), 130.7 (C-1), 129.1 (C-1a), 122.3 (C-2a), 121.1 (Ar-CH=*C*H), 114.3 (C-3a), 105.4 (C-2), 74.8 (*C*H(CH_3_)_2_), 56.3 (OCH_3_), 22.9 (CH(*C*H_3_)_2_). HRMS calc. for C_20_H_25_N_2_O_4_^+^ [M + H]^+^ 357.1809, found 357.1807.

(*E*)-*N*-(4-Aminophenyl)-3-(3,4,5-trimethoxyphenyl)acrylamide (**3h**): Title compound was obtained from **2h** (500 mg, 2.10 mmol), 4-phenylenediamine (227 mg, 2.10 mmol), EDCI (483 mg, 2.52 mmol), HOBt (340 mg, 2.52 mmol), and DIPEA (547 µL, 3.15 mmol). Orange gum (605 mg, 88%). ^1^H NMR (400 MHz, DMSO-*d*_6_) *δ* 9.79 (s, 1H, NH), 7.44 (d, 1H, *J* = 15.4 Hz, Ar-C*H*=CH), 7.35 (d, 2H, *J* = 8.6 Hz, H-2a), 6.92 (s, 2H, H-2), 6.70 (d, 1H, *J* = 15.4 Hz, Ar-CH=C*H*), 6.52 (d, 2H, *J* = 8.6 Hz, H-3a), 4.91 (s, 2H, NH_2_), 3.83 (s, 6H, 2 × 3C-OCH_3_), 3.70 (s, 3H, 4C-OCH_3_); ^13^C NMR (100.6 MHz, DMSO-*d*_6_) *δ* 162.6 (C=O), 153.0 (C-3), 144.9 (Ar-*C*H=CH), 139.1 (C-4a), 138.7 (C-4), 130.7 (C-1), 128.7 (C-1a), 122.2 (Ar-CH=*C*H), 120.6 (C-2a), 113.9 (C-3a), 105.0 (C-2), 60.2 (4C-OCH_3_), 55.9 (2 × 3C-OCH_3_). HRMS calc. for C_18_H_21_N_2_O_4_^+^ [M + H]^+^ 329.1496, found 329.1494. Data in accordance with the literature [54].

#### 3.1.2. General Procedure for the Synthesis of **4a**–**g**

To the corresponding isopropoxy derivative **3a**–**g**, dissolved in DCM (0.1 M), was added dropwise BCl_3_ (1 M in dichloromethane) (20 eq/isopropoxy group to remove) at −20 °C, which was stirred at this temperature for 2 h, then at 0 °C for 1 additional hour. The mixture was then hydrolyzed at 0 °C with water (2 × DCM volume), and the pH was adjusted to 7.0 with a 10% aqueous K_2_CO_3_. The resulting precipitate was filtered off, washed with water to afford the desired compound, and used without further purification.

(*E*)-*N*-(4-Aminophenyl)-3-(2-hydroxyphenyl)acrylamide (**4a**): Title compound was obtained from **3a** (770 mg, 2.55 mmol) and BCl_3_ in DCM (1 M, 51 mL, 51 mmol). Green pale powder (341 mg, 53%), mp 199.5–200.4 °C. ^1^H NMR (400 MHz, DMSO-*d*_6_) *δ* 10.11 (bs, 1H, OH), 9.80 (s, 1H, NH), 7.70 (d, 1H, *J* = 15.4 Hz, Ar-C*H*=CH), 7.45 (d, 1H, *J* = 8.8 Hz, H-6), 7.37 (d, 2H, *J* = 8.6 Hz, H-2a), 7.19 (t, 1H, *J* = 7.5 Hz, H-4), 6.93 (d, 1H, *J* = 7.5 Hz, H-5), 6.85 (d, 1H, *J* = 15.4 Hz, Ar-CH=C*H*), 6.83 (d, 1H, *J* = 7.5 Hz, H-3), 6.53 (d, 2H, *J* = 8.6 Hz, H-3a), 4.91 (bs, 2H, NH_2_); ^13^C NMR (100.6 MHz, DMSO-*d*_6_) *δ* 163.8 (C=O), 156.9 (C-2), 145.2 (C-4a), 135.3 (Ar-*C*H=CH), 131.0 (C-4), 129.3 (C-1a), 128.8 (C-6), 122.7 (C-1), 122.3 (C-5), 121.2 (C-2a), 119.9 (Ar-CH=*C*H), 116.6 (C-3), 114.4 (C-3a). HRMS calc. for C_15_H_15_N_2_O_2_^+^ [M + H]^+^ 255.1128, found 255.1124.

(*E*)-*N*-(4-Aminophenyl)-3-(3-hydroxyphenyl)acrylamide (**4b**): Title compound was obtained from **3b** (1140 mg, 3.88 mmol) and BCl_3_ in DCM (1 M, 77 mL, 77 mmol). Brown pale powder (616 mg, 63%), mp > 280 °C. ^1^H NMR (300 MHz, DMSO-*d*_6_) *δ* 9.81 (s, 1H, NH), 9.59 (s, 1H, OH), 7.39 (d, 1H, *J* = 15.6 Hz, Ar-C*H*=CH), 7.35 (d, 2H, *J* = 8.7 Hz, H-2a), 7.22 (t, 1H, *J* = 7.9 Hz, H-5), 6.99 (d, 1H, *J* = 8.1 Hz, H-4), 6.97 (bs, 1H, H-2), 6.80 (dd, 1H, *J* = 8.1 and 2.1 Hz, H-6), 6.73 (d, 1H, *J* = 15.6 Hz, Ar-CH=C*H*), 6.53 (d, 2H, *J* = 8.7 Hz, H-3a), 4.87 (bs, 2H, NH_2_); ^13^C NMR (75 MHz, DMSO-*d*_6_) *δ* 163.0 (C=O), 158.1 (C-3), 145.3 (C-4a), 139.4 (C-1), 136.7 (Ar-*C*H=CH), 130.3 (C-5), 129.0 (C-1a), 123.0 (Ar-CH=*C*H), 121.2 (C-2a), 119.2 (C-4), 117.1 (C-6), 114.3 (C-3a), 114.1 (C-2). HRMS calc. for C_15_H_15_N_2_O_2_^+^ [M + H]^+^ 255.1128, found 255.1128.

(*E*)-*N*-(4-Aminophenyl)-3-(4-hydroxyphenyl)acrylamide (**4c**): Title compound was obtained from **3c** (1140 mg, 3.88 mmol) and BCl_3_ in DCM (1 M, 77 mL, 77 mmol). Brown pale powder (789 mg, 80%), mp > 280 °C. ^1^H NMR (300 MHz, DMSO-*d*_6_) *δ* 9.86 (s, 1H, OH), 9.71 (s, 1H, NH), 7.41 (d, 2H, *J* = 7.0 Hz, H-2), 7.40 (d, 1H, *J* = 17.2 Hz, Ar-C*H*=CH), 7.34 (d, 2H, *J* = 7.5 Hz, H-2a), 6.81 (d, 2H, *J* = 7.0 Hz, H-3), 6.59 (d, 1H, *J* = 17.2 Hz, Ar-CH=C*H*), 6.52 (d, 2H, *J* = 7.5 Hz, H-3a), 4.85 (bs, 2H, NH_2_); ^13^C NMR (75 MHz, DMSO-*d*_6_) *δ* 163.1 (C=O), 158.9 (C-4), 144.7 (C-4a), 138.9 (Ar-*C*H=CH), 129.2 (C-2), 128.8 (C-1a), 125.9 (C-1), 120.7 (C-2a), 119.2 (Ar-CH=*C*H), 115.8 (C-3), 113.9 (C-3a). HRMS calc. for C_15_H_15_N_2_O_2_^+^ [M + H]^+^ 255.1128, found 255.1127.

(*E*)-*N*-(4-Aminophenyl)-3-(3,5-dihydroxyphenyl)acrylamide (**4d**): Title compound was obtained from **3d** (670 mg, 1.89 mmol) and BCl_3_ in DCM (1 M, 75 mL, 75 mmol). Brown powder (510 mg, Quant.), mp 255.2–255.7 °C. ^1^H NMR (400 MHz, DMSO-*d*_6_) *δ* 9.77 (s, 1H, NH), 9.42 (s, 2H, OH), 7.32 (d, 2H, *J* = 8.5 Hz, H-2a), 7.25 (d, 1H, *J* = 15.4 Hz, Ar-C*H*=CH), 6.60 (d, 1H, *J* = 15.4 Hz, Ar-CH=C*H*), 6.51 (d, 2H, *J* = 8.5 Hz, H-3a), 6.41 (d, 2H, *J* = 1.9 Hz, H-2), 6.24 (t, 1H, *J* = 1.9 Hz, H-4), 4.91 (bs, 2H, NH_2_); ^13^C NMR (100.6 MHz, DMSO-*d*_6_) *δ* 162.7 (C=O), 158.7 (C-3), 144.9 (Ar-*C*H=CH), 139.4 (C-4a), 136.6 (C-1), 128.5 (C-1a), 122.3 (Ar-CH=*C*H), 120.8 (C-2a), 113.9 (C-3a), 105.6 (C-2), 103.9 (C-4). HRMS calc. for C_15_H_15_N_2_O_3_^+^ [M + H]^+^ 271.1077, found 271.1078.

(*E*)-*N*-(4-Aminophenyl)-3-(3,4-dihydroxyphenyl)acrylamide (**4e**): Title compound was obtained from **3e** (2.0 g, 5.65 mmol) and BCl_3_ in DCM (112 mL, 112 mmol, 20 eq). Brown powder (1.35 g, 88%), mp 164 °C. ^1^H NMR (300 MHz, DMSO-*d*_6_) *δ* 9.56 (bs, 1H, NHCO), 7.33 (d, 2H, *J* = 8.4 Hz, H-2a), 7.29 (d, 1H, *J* = 15.2 Hz, Ar-C*H*=CH), 6.82 (bs, 2H, H-2 and OH), 6.61 (bd, 1H, *J* = 8.1 Hz, H-6), 6.51 (d, 2H, *J* = 8.4 Hz, H-3a), 6.38 (d, 1H, *J* = 15.2 Hz, Ar-CH=C*H*), 6.33 (d, 1H, *J* = 8.1 Hz, H-5), 4.86 (bs, 2H, NH_2_); ^13^C NMR (75 MHz, DMSO-*d*_6_) *δ* 164.2 (C=O), 156.0 (C-4), 153.8 (C-3), 144.9 (C-4a), 141.9 (Ar-*C*H=CH), 129.6 (C-1), 124.4 (C-1a), 121.3 (C-6), 121.1 (C-2a), 116.6 (Ar-CH=*C*H), 114.5 (C-3a), 107.3 (C-5), 104.2 (C-2). HRMS calc. for C_15_H_15_N_2_O_3_^+^ [M + H]^+^ 271.1077, found 271.1076.

(*E*)-*N*-(4-Aminophenyl)-3-(4-hydroxy-3-methoxyphenyl)acrylamide (**4f**): Title compound was obtained from **3f** (1.0 g, 3.30 mmol) and BCl_3_ in DCM (61.3 mL, 61.3 mmol, 20 eq). Brown powder (446 mg, 48%), mp 254 °C. ^1^H NMR (300 MHz, DMSO-*d*_6_) *δ* 9.74 (bs, 1H, NHCO), 7.38 (d, 1H, *J* = 16.3 Hz, Ar-C*H*=CH), 7.36 (d, 2H, *J* = 8.4 Hz, H-2a), 7.10 (bs, 1H, H-2), 6.98 (d, 1H, *J* = 7.9 Hz, H-6), 6.78 (d, 1H, *J* = 7.9 Hz, H-5), 6.57 (d, 1H, *J* = 16.3 Hz, Ar-CH=C*H*), 6.53 (d, 2H, *J* = 8.4 Hz, H-3a), 4.86 (bs, 2H, NH_2_), 3.79 (s, 3H, OCH_3_); ^13^C NMR (75 MHz, DMSO-*d*_6_) *δ* 163.8 (C=O), 148.9 (C-4), 145.1 (C-3), 140.1 (C-1), 129.5 (C-1a), 125.2 (Ar-*C*H=CH), 122.6 (C-6), 121.1 (C-2a), 118.7 (Ar-CH=*C*H), 116.5 (C-5), 114.4 (C-3a), 111.1 (C-2), 55.z9 (OCH_3_). HRMS calc. for C_16_H_17_N_2_O_3_^+^ [M + H]^+^ 285.1234, found 285.1233.

(*E*)-*N*-(4-Aminophenyl)-3-(4-hydroxy-3,5-dimethoxyphenyl)acrylamide (**4g**): Title compound was obtained from **3g** (1.0 g, 2.81 mmol) and BCl_3_ in DCM (56 mL, 56 mmol, 20 eq). Brown powder (417 mg, 47%), mp 264 °C. ^1^H NMR (300 MHz, DMSO-*d*_6_) *δ* 9.70 (bs, 1H, NHCO), 8.83 (bs, 1H, OH), 7.40 (d, 1H, *J* = 15.6 Hz, Ar-C*H*=CH), 7.32 (d, 2H, *J* = 8.4 Hz, H-2a), 6.89 (bs, 2H, H-2), 6.62 (d, 1H, *J* = 15.6 Hz, Ar-CH=C*H*), 6.52 (d, 2H, *J* = 8.4 Hz, H-3a), 4.88 (bs, 2H, NH_2_), 3.81 (s, 6H, 2 × OCH_3_); ^13^C NMR (75 MHz, DMSO-*d*_6_) *δ* 163.5 (C=O), 148.6 (C-3), 145.3 (C-4a), 140.1 (Ar-*C*H=CH), 137.9 (C-4), 129.3 (C-1), 125.8 (C-1a), 121.1 (C-2a), 120.3 (Ar-CH=*C*H), 114.4 (C-3a), 105.7 (C-2), 56.5 (OCH_3_). HRMS calc. for C_17_H_19_N_2_O_3_^+^ [M + H]^+^ 315.1339, found 315.1342.

#### 3.1.3. General Procedure for the Synthesis of **5a**–**h**

To a mixture of the corresponding compound **4a**–**h** and cyanoguanidine (1.2 eq), dissolved in acetonitrile (to obtain 0.1 M of free amine), was added a catalytic amount of concentrated hydrochloric acid (two drops), and the mixture was heated at 125 °C for 10 to 15 min in a microwave reactor. The mixture was cooled at room temperature and filtered to afford the desired compound, used without further purification.

(*E*)-*N*-(4-(3-Carbamimidoylguanidino)phenyl)-3-(2-hydroxyphenyl)acrylamide (**5a**): Title compound was obtained from **4a** (341 mg, 1.34 mmol) and cyanoguanidine (135 mg, 1.61 mmol). Orange powder (362 mg, 80%), mp > 280 °C. ^1^H NMR (400 MHz, DMSO-*d*_6_) *δ* 10.22 (bs, 1H, CONH), 7.76 (d, 1H, *J* = 15.7 Hz, Ar-C*H*=CH), 7.59 (d, 2H, *J* = 8.1 Hz, H-2a), 7.47 (dd, 1H, *J* = 7.7 and 1.7 Hz, H-6), 7.20 (td, 1H, *J* = 7.7 and 2.1 Hz, H-5), 6.96 (bd, 1H, *J* = 8.1 Hz, H-3), 6.94 (d, 1H, *J* = 15.7 Hz, Ar-CH=C*H*), 6.84 (t, 1H, *J* = 7.5 Hz, H-4), 6.54 (d, 2H, *J* = 8.1 Hz, H-3a), 6.51 (s, 1H, NH), 4.86 (s, 2H, NH_2_); ^13^C NMR (100.6 MHz, DMSO-*d*_6_) *δ* 164.4 (C=O), 159.2 (C=NH), 157.0 (C-2), 136.3 (Ar-*C*H=CH), 135.4 (C-4a), 131.7 (C-1a), 131.1 (C-1 and C-4), 129.0 (C-6), 122.3 (C-2a), 122.1 (Ar-CH=*C*H), 120.0 (C-5), 119.8 (C-3), 116.6 (C-3a). HRMS calc. for C_17_H_19_N_6_O_2_^+^ [M + H]^+^ 339.1564 found 339.1569. Purity (HPLC): 99%, t_r_ = 5.2 min.

(*E*)-*N*-(4-(3-Carbamimidoylguanidino)phenyl)-3-(3-hydroxyphenyl)acrylamide (**5b**): Title compound was obtained from **4b** (341 mg, 1.34 mmol) and cyanoguanidine (135 mg, 1.61 mmol). Pink powder (320 mg, 71%), mp > 280 °C. ^1^H NMR (400 MHz, DMSO-*d*_6_) *δ* 11.13 (bs, 1H, CONH), 8.67 (s, 1H, NH), 8.27 (d, 2H, *J* = 8.2 Hz, H-2a), 7.93 (d, 1H, *J* = 15.6 Hz, Ar-C*H*=CH), 7.81 (d, 2H, *J* = 8.2 Hz, H-3a), 7.66 (t, 1H, *J* = 7.5 Hz, H-5), 7.47–7.45 (m, 2H, H-2 and H-4), 7.32 (d, 1H, *J* = 15.6 Hz, Ar-CH=C*H*), 7.27 (dd, 1H, *J* = 7.8 and 1.8 Hz, H-6), 7.14 (s, 1H, NH); ^13^C NMR (100.6 MHz, DMSO-*d*_6_) *δ* 164.0 (C=O), 157.8 (C-3), 155.8 (C=NH), 140.6 (C-4a), 139.2 (C-1), 135.9 (C-1a), 129.9 (C-5), 126.5 (Ar-*C*H=CH), 123.7 (C-3a), 121.9 (Ar-CH=*C*H), 120.1 (C-2a), 118.9 (C-2), 118.0 (C-6), 113.9 (C-4). HRMS calc. for C_17_H_19_N_6_O_2_^+^ [M + H]^+^ 339.1564, found 339.1566. Purity (HPLC): 97%, t_r_ = 5.3 min.

(*E*)-*N*-(4-(3-Carbamimidoylguanidino)phenyl)-3-(4-hydroxyphenyl)acrylamide (**5c**): Title compound was obtained from **4c** (341 mg, 1.34 mmol) and cyanoguanidine (135 mg, 1.61 mmol). Yellow powder (320 mg, 71%), mp > 280 °C. ^1^H NMR (400 MHz, DMSO-*d*_6_) *δ* 10.98 (bs, 1H, CONH), 10.85 (s, 1H, OH or NH), 8.66 (s, 1H, NH), 8.25 (d, 2H, *J* = 8.7 Hz, H-2a), 7.92 (d, 1H, *J* = 15.4 Hz, Ar-C*H*=CH), 7.88 (d, 2H, *J* = 8.6 Hz, H-2), 7.78 (d, 2H, *J* = 8.8 Hz, H-3a), 7.64 (s, 1H, NH), 7.58 (s, 1H, NH), 7.27 (d, 2H, *J* = 8.6 Hz, H-3), 7.15 (d, 1H, *J* = 15.6 Hz, Ar-CH=C*H*), 4.08 (bs, 3H, NH and NH_2_); ^13^C NMR (100.6 MHz, DMSO-*d*_6_) *δ* 164.4 (C=O), 159.3 (C-4), 155.6 (C=NH), 154.5 (C=NH), 140.6 (Ar-*C*H=CH), 139.3 (C-4a), 129.5 (C-3a), 126.2 (C-1), 125.6 (C-1a), 123.7 (C-2), 119.9 (C-2a), 118.4 (Ar-CH=*C*H), 115.9 (C-3). HRMS calc. for C_17_H_19_N_6_O_2_^+^ [M + H]^+^ 339.1564, found 339.1565. Purity (HPLC): 96%, t_r_ = 4.8 min.

(*E*)-*N*-(4-(3-Carbamimidoylguanidino)phenyl)-3-(3,5-dihydroxyphenyl)acrylamide (**5d**): Title compound was obtained from **4d** (510 mg, 1.89 mmol) and cyanoguanidine (191 mg, 2.27 mmol). Brown powder (227 mg, 34%), mp > 180 °C. ^1^H NMR (400 MHz, DMSO-*d*_6_) *δ* 10.32 (bs, 1H, CONH), 10.27 (s, 1H, NH), 9.63 (s, 1H, NH), 8.18 (s, 2H, OH), 7.65 (d, 2H, *J* = 8.2 Hz, H-2a), 7.37 (d, 1H, *J* = 15.3 Hz, Ar-C*H*=CH), 7.28 (d, 2H, *J* = 8.2 Hz, H-3a), 7.10 (bs, 2H, NH_2_), 6.70 (d, 1H, *J* = 15.3 Hz, Ar-CH=C*H*), 6.45 (m, 2H, H-2), 6.28 (m, 1H, H-4); ^13^C NMR (100.6 MHz, DMSO-*d*_6_) *δ* 163.7 (C=O), 158.7 (C-3), 155.5 (C=NH), 154.6 (C=NH), 140.6 (Ar-*C*H=CH), 136.5 (C-1), 135.6 (C-4a), 122.3 (C-2a), 121.9 (C-1a), 119.7 (C-3a), 118.2 (Ar-CH=*C*H), 106.1 (C-2), 104.4 (C-4). HRMS calc. for C_17_H_19_N_6_O_3_^+^ [M + H]^+^ 354.1440, found 354.1438. Purity (HPLC): 98%, t_r_ = 3.8 min.

(*E*)-*N*-(4-(3-Carbamimidoylguanidino)phenyl)-3-(3,4-dihydroxyphenyl)acrylamide (**5e**): Title compound was obtained from **4e** (250 mg, 0.95 mmol) and cyanoguanidine (95 mg, 1.11 mmol). Orange powder (319 mg, 95%), mp 207 °C. ^1^H NMR (300 MHz, DMSO-*d*_6_) *δ* 10.16 (bs, 1H, NHCO), 8.21 (bs, 1H, OH), 7.70 (d, 2H, *J* = 7.7 Hz, H-2a), 7.43 (d, 1H, *J* = 15.2 Hz, Ar-C*H*=CH), 7.15 (d, 2H, *J* = 7.6 Hz, H-3a), 7.02 (bs, 1H, H exchangeable), 6.83 (bs, 1H, H-2), 6.69 (d, 1H, *J* = 15.2 Hz, Ar-CH=C*H*), 6.56 (bd, 1H, *J* = 7.8 Hz, H-6), 6.53 (d, 1H, *J* = 7.8 Hz, H-5); ^13^C NMR (75 MHz, DMSO-*d*_6_) *δ* 164.8 (C=O), 164.5 (C=NH), 154.6 (C=NH), 152.4 (C-4), 148.2 (C-3), 146.1 (C-4a), 142.5 (Ar-*C*H=CH), 141.0 (C-1), 126.7 (C-1a), 125.5 (C-2a), 121.5 (C-6), 121.3 (C-3a), 120.5 (Ar-CH=*C*H), 116.3 (C-5), 114.5 (C-2). HRMS calc. for C_17_H_19_N_6_O_3_^+^ [M + H]^+^ 355.1513, found 355.1510. Purity (HPLC): 97%, t_r_ = 4.0 min.

(*E*)-*N*-(4-(3-Carbamimidoylguanidino)phenyl)-3-(4-hydroxy-3-methoxyphenyl) acrylamide (**5f**): Title compound was obtained from **4f** (200 mg, 0.70 mmol) and cyanoguanidine (71 mg, 0.85 mmol). Orange powder (240 mg, 93%), mp 230 °C. ^1^H NMR (300 MHz, DMSO-*d*_6_) *δ* 10.54 (bs, 1 H, NHCO), 8.24 (bs, 1H, OH), 7.83 (d, 2H, *J* = 8.6 Hz, H-2a), 7.51 (d, 1H, *J* = 15.6 Hz, Ar-C*H*=CH), 7.36 (d, 2H, *J* = 8.6 Hz, H-3a), 7.19 (bs, 1H, H-2), 7.08 (bd, 1H, *J* = 7.9 Hz, H-6), 6.77 (d, 1 H, *J* = 15.6 Hz, Ar-CH=C*H*), 6.73 (d, 1H, *J* = 7.9 Hz, H-5), 6.71 (bs, 1H, NH), 3.82 (s, 3H, OCH_3_); ^13^C NMR (75 MHz, DMSO-*d*_6_) *δ* 164.8 (C=O), 164.4 (C=NH), 149.3 (C-4), 148.4 (C-3), 141.4 (C-4a), 140.8 (C=NH), 139.8 (C-1), 126.6 (C-1a), 124.2 (C-2a), 122.5 (Ar-*C*H=CH), 120.4 (C-3a), 119.9 (C-6), 119.2 (Ar-CH=*C*H), 116.2 (C-5), 111.5 (C-2), 56.0 (OCH_3_). HRMS calc. for C_18_H_21_N_6_O_3_^+^ [M + H]^+^ 369.1670, found 369.1671. Purity (HPLC): 99%, t_r_ = 4.9 min.

(*E*)-*N*-(4-(3-Carbamimidoylguanidino)phenyl)-3-(4-hydroxy-3,5-dimethoxyphenyl)acrylamide (**5g**): Title compound was obtained from **4g** (100 mg, 0.32 mmol) and cyanoguanidine (32 mg, 0.38 mmol). Pink pale powder (115 mg, 90%), mp 215 °C. ^1^H NMR (300 MHz, DMSO-*d*_6_) *δ* 10.46 (bs, 1H, NHCO), 7.82 (d, 2H, *J* = 8.6 Hz, H-2a), 7.51 (d, 1H, *J* = 15.6 Hz, Ar-C*H*=CH), 7.36 (d, 2H, *J* = 8.6 Hz, H-3a), 6.93 (bs, 2H, H-2), 6.75 (d, 1H, *J* = 15.6 Hz, Ar-CH=C*H*), 6.65 (bs, 3H, NH, NH_2_), 3.81 (s, 6H, 2 × OCH_3_); ^13^C NMR (75 MHz, DMSO-*d*_6_) *δ* 164.7 (C=O), 164.4 (C=NH), 148.6 (C-3), 141.7 (C=NH), 139.9 (Ar-*C*H=CH), 138.3 (C-4, C-4a), 126.7 (C-1), 125.5 (C-1a), 124.2 (C-2a), 120.4 (C-3a), 119.6 (Ar-CH=*C*H), 106.0 (C-2), 56.5 (OCH_3_). HRMS calc. for C_19_H_23_N_6_O_4_^+^ [M + H]^+^ 399.1775, found 399.1772. Purity (HPLC): 97%, t_r_ = 4.8 min.

(*E*)-*N*-(4-(3-Carbamimidoylguanidino)phenyl)-3-(3,4,5-trimethoxyphenyl) acrylamide (**5h**): Title compound was obtained from **3h** (600 mg, 1.83 mmol) and cyanoguanidine (184 mg, 2.20 mmol). Yellow-brown powder (392 mg, 76%), mp > 180 °C. ^1^H NMR (400 MHz, DMSO-*d*_6_) *δ* 10.68 (bs, 1H, CONH), 10.59 (s, 1H, NH), 10.37 (s, 1H, NH), 8.26 (s, 1H, NH), 7.76 (d, 2H, *J* = 8.7 Hz, H-2a), 7.55 (d, 1H, *J* = 15.7 Hz, Ar-C*H*=CH), 7.25 (d, 2H, *J* = 8.7 Hz, H-3a), 7.27 (s, 1H, NH), 7.20 (s, 2H, NH_2_), 6.97 (s, 2H, H-2), 6.77 (d, 1H, *J* = 15.7 Hz, Ar-CH=C*H*), 3.85 (s, 6H, 3C-O*C*H_3_), 3.71 (s, 3H, 4C-O*C*H_3_); ^13^C NMR (100.6 MHz, DMSO-*d*_6_) *δ* 164.0 (C=O), 163.8 (C=NH), 162.4 (C=NH), 158.5 (C-3), 154.6 (C-4a), 140.5 (Ar-*C*H=CH), 140.1 (C-4), 130.5 (C-1), 126.4 (C-1a), 124.0 (C-2a), 121.7 (Ar-CH=*C*H), 120.0 (C-3a), 105.3 (C-2), 60.2 (4C-OCH_3_), 56.0 (3C-OCH_3_). HRMS calc. for C_20_H_25_N_6_O_4_^+^ [M + H]^+^ 412.1859, found 412.1857. Purity (HPLC): >99%, t_r_ = 7.8 min.

#### 3.1.4. (*E*)-*N*-(4-Aminophenyl)-3-(3,5-diisopropoxyphenyl)-2-(4-isoproproxyphenyl) acrylamide (**8**)

Title compound was obtained from 7 (347 mg, 711 µmol), 4-phenylenediamine (78 mg, 711 µmol), EDCI (164 mg, 853 µmol), HOBt (115 mg, 853 µmol), and DIPEA (185 µL, 1.07 mmol) according to the procedure described in 3.1.1. Brown powder (346 mg, quant. yield), mp 264 °C. ^1^H NMR (400 MHz, DMSO-*d*_6_) δ 9.13 (s, 1H, NH), 7.26 (d, 2H, *J* = 8.8 Hz, H_ortho monosubstituted_), 7.21 (s, 1H, Ar-CH=C), 7.14 (d, 2H, *J* = 8.6 Hz, H-2a), 6.96 (d, 2H, *J* = 8.6 Hz, H-3a), 6.49 (d, 2H, *J* = 8.8 Hz, H_meta monosubstituted_), 6.22 (t, 1H, *J* = 1.9 Hz, H_para disubstituted_), 6.20 (d, 2H, *J* = 1.9 Hz, H_ortho disubstituted_), 4.90 (s, 2H, NH_2_), 4.62 (sept, 1H, *J* = 5.8 Hz, CH(CH_3_)_2_), 4.24 (sept, 2H, *J* = 5.8 Hz, 2 × CH(CH_3_)_2_), 1.28 (d, 6H, *J* = 5.82 Hz, CH(CH_3_)_2_), 1.10 (d, 12H, *J* = 5.8 Hz, 2 × CH(CH_3_)_2_); ^13^C NMR (100.6 MHz, DMSO-*d*_6_) δ 166.2 (C=O), 158.0 (C_meta disubstituted_), 157.1 (C_para monosubstituted_), 145.0 (C-4a), 137.4 (Ar-CH=C), 137.0 (C_ipso disubstituted_), 132.9 (Ar-CH=C and C_ipso monosubstituted_), 130.6 (C_ortho monosubstituted_), 127.9 (C-1a), 121.8 (C-2a), 115.7 (C-3a), 113.6 (C_meta monosubstituted_), 108.8 (C_ortho disubstituted_), 104.1 (_Cpara disubstituted_), 69.0 (3 × CH(CH_3_)_2_), 21.8 (3 × CH(CH_3_)_2_). HRMS calc. for C_30_H_37_N_2_O_4_^+^ [M + H]^+^ 489.2748, found 489.2747.

#### 3.1.5. (*E*)-*N*-(4-Aminophenyl)-3-(3,5-dihydroxyphenyl)-2-(4-hydroxyphenyl)acrylamide (**9**)

Title compound was obtained from **8** (347 mg, 711 µmol) and BCl_3_ in DCM (1 M, 43 mL, 43 mmol) according to the procedure described in 3.1.2. Brown powder (257 mg, quant. yield), mp > 280 °C. ^1^H NMR (400 MHz, DMSO-*d*_6_) *δ* 8.97 (s, 1H, NH), 7.23 (d, 2H, *J* = 8.6 Hz, H*_ortho_*_monosubstituted_), 7.01 (s, 1H, Ar-CH=C), 6.99 (d, 2H, *J* = 8.5 Hz, H-2a), 6.74 (d, 2H, *J* = 8.5 Hz, H-3a), 6.49 (d, 2H, *J* = 8.6 Hz, H*_meta_*_monosubstituted_), 6.09 (t, 1H, *J* = 1.9 Hz, H*_para_*_disubstituted_), 5.97 (d, 2H, *J* = 1.9 Hz, H*_ortho_*_disubstituted_), 4.87 (s, 2H, NH_2_); ^13^C NMR (100.6 MHz, DMSO-*d*_6_) *δ* 166.8 (C=O), 158.0 (C*_meta_*_disubstituted_), 157.5 (C*_para_*_monosubstituted_), 145.0 (C-4a), 137.1 (Ar-*C*H=C), 137.0 (C*_ipso_*_disubstituted_), 132.7 (Ar-CH=*C*), 130.6 (C*_ortho_*_monosubstituted_), 128.2 (C-1a), 126.1 (C*_ipso_*_monosubstituted_), 121.7 (C-2a), 115.7 (C-3a), 113.6 (C*_meta_*_monosubstituted_), 108.0 (C*_ortho_*_disubstituted_), 102.7 (C*_para_*_disubstituted_). HRMS calc. for C_21_H_19_N_2_O_4_^+^ [M + H]^+^ 363.1339, found 363.1342.

#### 3.1.6. (*E*)-*N*-(4-(3-Carbamimidoylguanidino)phenyl)-3-(3,5-dihydroxyphenyl)-2-(4-hydrophenyl)acrylamide (**10**)

Title compound was obtained from 9 (257 mg, 711 µmol) and cyanoguanidine (72 mg, 853 µmmol) according to the general procedure. Brown powder (193 mg, 61%), mp > 280 °C. ^1^H NMR (400 MHz, DMSO-*d*_6_) δ 10.30 (s, 1H, CONH), 9.88 (s, 1H, OH), 9.63 (s, 1H, OH), 7.77 (d, 2H, *J* = 8.9 Hz, H-2a), 7.33 (d, 2H, *J* = 8.9 Hz, H-3a), 7.10 (s, 1H, NH), 7.06 (s, 1H, Ar-CH=C), 7.02 (d, 2H, *J* = 8.5 Hz, H_ortho monosubstituted_), 6.77 (d, 2H, *J* = 8.5 Hz, H_meta monosubstituted_), 6.13 (m, 1H, H_para disubstituted_), 6.02 (m, 2H, H_ortho disubstituted_), 3.84 (s, 2H, NH_2_), 3.71 (s, 1H, NH); ^13^C NMR (100.6 MHz, DMSO-*d*_6_) δ 168.2 (C=O), 163.6 (C=NH), 162.8 (C=NH), 157.8 (C-4a), 155.4 (C_para monosubstituted_), 154.4 (C_meta disubstituted_), 140.1 (Ar-CH=C), 131.3 (C_ipso disubstituted_), 131.0 (Ar-CH=C), 130.6 (C_ortho monosubstituted_), 127.0 (C-1a), 123.3 (C-2a), 120.8 (C-3a), 117.9 (C_meta monosubstituted_), 115.3 (C_ipso monosubstituted_), 107.8 (C_ortho disubstituted_), 100.6 (C_para disubstituted_). HRMS calc. for C_23_H_23_N_6_O_4_^+^ [M + H]^+^ 446.1703, found 446.1706. Purity (HPLC): 96%, t_r_ = 5.9 min.

### 3.2. Biology

#### 3.2.1. Chemicals and Antibodies

The following chemicals were used: Metformin (Sigma-Aldrich) stored at 500 mM in media and used at different concentrations; apo-Transferrin (Sigma-Aldrich) stored at 100 mg·mL^−1^ in PBS and used at 100 µg·mL^−1^; insulin (Sigma-Aldrich) stored at 0.5 mg·mL^−1^ in hydrochloric acid (0.01 M) and used at 5 µg·mL^−1^; progesterone (Sigma-Aldrich) stored at 2.0 × 10^−5^ M in sterile water and used at 2 × 10^−8^ M; sodium selenite (Sigma-Aldrich) stored at 1.5 × 10^−5^ M in sterile water and used at 30 nM; poly-DL-ornithine (Sigma-Aldrich) stored at 10 mg·mL^−1^ in PBS and used at 10 µg·mL^−1^; (3-(4,5-dimethylthiazol-2-yl)-2,5-diphenyl tetrazolium bromide) (Sigma-Aldrich) used at 0.5 mg·mL^−1^ in media; putrescine dihydrochloride (Sigma-Aldrich) stored at 20 mM in PBS and used at 0.1 mM; basic Fibroblast Growth factor (bFGF, Thermo Fisher Scientific, Illkirch, France) stored at 10 µg·mL^−1^ in Tris (10 mM, pH 7.6, 0.1% BSA) and used at 10 ng·mL^−1^; B27 (Thermo Fisher Scientific) used at 1X; L-glutamine (Thermo Fisher Scientific) used at 1X; plasmocin (InvivoGen SAS, Toulouse, France) used at 5 µg·mL^−1^; Epidermal Growth Factor (EGF, R&D System, Bio-Techne SAS, Seiche, France) stored at 100 µg·mL^−1^ in acetic acid (10 mM, 0.1% BSA) and used at 20 ng·mL^−1^. Dulbecco’s modified Eagle’s medium (DMEM), F12 media, Trypsin-EDTA (1X), Fetal Bovine serum (FBS), Mammalian Protein Extraction Reagent (M-PER), phosphatases and proteases inhibitors, 2′,7′-dichlorodihydrofluorescein diacetate (DCFDA), and mitoSOX were obtained from Thermo Fisher Scientific. Eagle’s minimal essential medium (EMEM) was purchased from Lonza (Basel, Switzerland). Dulbecco’s Phosphate-Buffered Saline (PBS), Accumax solution, HDFs (ref 106-05A), Fibroblast Growth Media (FGM; ref 116-500), Bovine Serum albumin (BSA), DL-dithiothreitol (DTT), acrylamide mix solution (30%), Tris solution (1.5 M, pH 8.8), sodium dodecyl sulfate (SDS), ammonium persulfate (APS), tetramethylethylenediamine (TEMED), Tris-buffered saline (TBS), Tween 20, NADPH cofactor, lucigenin (*N,N*-dimethyl-9,9-biacridinium dinitrate), and temozolomide (TMZ) were obtained from Sigma-Aldrich. Bradford reagent and Protein Assay Dye Reagent Concentrate were purchased from Bio-Rad (Marnes-la-Coquette, France), and bromophenol blue from Thistle Scientific (Glasgow, UK). The following primary antibodies were used: anti-GAPDH (1/10 000, ref. G8795); anti-AKT and anti-Phospho AKT Ser473 (1/1 000, ref 9272 and 9271, respectively; Cell Signaling Technology (Leiden, The Netherlands); anti-AMPKα and anti-Phospho AMPKα Thr172 (1/2 000, ref 2532 and 4188, respectively; Cell Signaling Technology); anti-p-44-42 and anti-Phospho-p-44-42 (1/1 000, ref 9102 and 9101 respectively, Cell Signaling Technology). The HRP-coupled secondary antibodies were purchased also from Cell Signaling Technology. The mitochondria-targeted nitrone [4-[4-[[(1,1-Dimethylethyl)-oxidoimino]methyl]phenoxy] butyl]triphenylphosphonium bromide (mito-PBN) was synthesized as previously described [45] and used for investigating oxidative stress U87-MG cells. The U251 and U87 MG cell lines were obtained from European Collection of authenticated cell lines (ECACC). The GBM6 and GBM9 cancerous stem cell were obtained from Dr Aurélie Tchoghandjian, Glioma team 7, Institute of NeuroPhysiopathology (INP, UMR7051 CNRS-Aix Marseille University).

#### 3.2.2. Culture Conditions

Two human glioma cell lines, U87-MG and U251, were used and routinely maintained in EMEM and DMEM, respectively, supplemented with 10% FBS and 1% L-glutamine (100X) at 37 °C in a humidified atmosphere with 5% CO_2_. U87-MG and U251 cell lines are widely used to model glioblastoma due to their rapid proliferation and treatment resistance. Two human primary glioblastoma stem cell lines from a glioblastoma tumor sample close to the subventricular zone and a cortical glioblastoma, GBM6 and GBM9, respectively, were used. GBM6 and GBM9 stem-like cells, derived from primary tumors, represent a more relevant model for studying cancer stem cells, which are responsible for relapse. The cells were grown in DMEM/F12 medium supplemented with hormones (insulin, putrescine, progesterone, apo-transferrin, sodium selenite) and growth factors (bFGF, EGF, and B27) as a stem cell-permissive medium which allowed the formation of spheres. They were maintained at 37 °C in a humidified atmosphere with 5% CO_2_. The cells were dissociated using Accumax solution (Sigma-Aldrich) and expanded every week. Primary human dermal fibroblasts (HDFs) were used as a non-cancer cell line for control and maintained in FGM at 37 °C in a humidified atmosphere with 5% CO_2_.

#### 3.2.3. Cytotoxicity Assays

Cell viability was determined using the MTT assay, based on the ability of the mitochondrial dehydrogenase enzyme to convert the yellow water soluble 3-(4,5-dimethylthiazol-2-yl)-2,5-diphenyl tetrazolium bromide (MTT) into a violet formazan compound. After counting and plating HDF, U87-MG, or U251 cells (50,000 cells/mL for U87-MG and U251, 30,000 cells/mL for HDFs, 100 µL per well) in appropriate media, the cells were exposed to increasing concentrations of the drugs (from 0 to 500 µM for hybrids and 0 to 20 mM for metformin) for 72 h. Cells were treated with 0.5 mg/mL of MTT in corresponding media (FGM for HDFs, EMEM for U87-MG, and DMEM for U251) for 2 h at 37 °C in a 5% CO_2_-humidified incubator. After incubation, the cells were lysed, and the formazan was solubilized using pure DMSO. The MTT-formazan conversion was analyzed through spectrophotometry at 600 nm in a plate reader (Multiskan RC, Labsystems, Vantaa, Finland). The data were expressed as percentage of survival (using untreated cells as 100%), and IC_50_ values (in µM) were determined. For GBM6 and 9 cells, the plates were coated with poly-DL-ornithine 24 h before, and the cells were seeded at a density of 100,000 cells/mL, 100 µL per well. The data obtained with MTT were confirmed using crystal violet (Sigma-Aldrich) staining in the same conditions [25]. The IC_50_ values were determined from dose–response curves according to the median effect principle described by Chou and Talalay [55].

#### 3.2.4. Preparation of Cell Extracts

The cells were washed in ice-cold PBS and lysed in M-PER lysis buffer containing protease and phosphatase inhibitors, according to a reported protocol [56]. Lysates were centrifuged at 12,000 rpm for 10 min at 4 °C. A protein quantification assay was then performed using the Protein Assay Dye Reagent Concentrate. Loading buffer was prepared (Laemmli sample buffer, 62.5 mM, Tris-HCl pH 6.8, 25% glycerol, 2% SDS, bromophenol blue, 350 mM dithiothreitol (DTT)) and added to the proteins. Samples were denatured at 95 °C for 5 min.

#### 3.2.5. Western Blot Analysis

According to a reported protocol [56], equal amounts of proteins (30 µg) were separated using SDS-PAGE gels. The proteins were transferred onto Nitrocellulose Blotting Membrane (Amersham Protan, GE Healthcare, GE Healthcare Partners, Vélizy, France). The membranes were blocked for 30 min in 5% nonfat milk in PBST (PBS plus 0.05% Tween 20) and then incubated overnight with the appropriate primary antibodies. After PBST washes, the membranes were incubated with horseradish–peroxidase-conjugated secondary antibodies for 1 h at room temperature. They were again washed with PBST and revealed using chemiluminescence HRP substrate (Merck Millipore, Lyon, France). Specific signals were detected by the G-Box (Syngene, Illkirch, France). The band intensities were quantified using the NIH ImageJ software version 2.16.0/1.54p. 

#### 3.2.6. Kinase Activity Assay

PamGene’s functional kinase assay detects protein kinase activity directly in cellular and tissue lysates, through measuring peptide phosphorylation by protein kinases (https://pamgene.com/, accessed on 25 March 2025). A PamChip incorporates 144 (serine–threonine kinase substrates) peptide sequences thirteen amino acids in length, immobilized on a porous ceramic membrane. A combination of phosphorylated peptides was compared to a comprehensive, integrated database (DB) of potential kinases that are linked to the peptides on the PamChips. This corresponds to around 350 unique kinases in the literature, covering the majority of the kinome. Following 4 h of exposure to metformin or its hybrid derivatives (**5a** and **5h**), U87-MG and GBM9 cells were lysed using M-PER lysis buffer. Protein concentration was quantified using the Protein Assay Dye Reagent Concentrate, and 10 µg of total protein was loaded per PamChip array. Phosphorylation events were detected using an FITC-conjugated phospho-specific antibody and recorded on a PamStation^®^12 imaging system (PamGene International B.V, Hertogenbosch, The Netherlands). Each condition was analyzed in three independent technical replicates.

#### 3.2.7. Measurement of Extracellular Redox State Variation

According to a previous procedure [56], cells were seeded on a white 96-well plate (1000 cells per well) and treated as indicated. Superoxide production was assessed by adding NADPH (1 mM) and lucigenin (10 μM) and recording luminescence every 45 min using a Fluoroskan plate reader (Fisher Scientific SAS, Illkirch, France). After measurement, cells were fixed (1% glutaraldehyde) for 10 min, stained with crystal violet (0.1%) for 30 min, washed with PBS, and lysed in DMSO. The optical densities (ODs) were recorded using a Multiskan plate reader (Fisher Scientific SAS, Illkirch, France). to quantify cell numbers, and luminescence values (RLU) were normalized using the crystal violet OD values. Data were compared to the control condition and expressed as a percentage.

#### 3.2.8. Measurement of Intracellular Redox State Variation

The intracellular oxidative stress was measured using DCFDA according to a reported protocol [56]. Cells were seeded in black 96-well plates (20,000 cells per well). The culture media was replaced with measurement buffer supplemented with DCFDA (10 μM) for 30 min, and cells were washed with measurement buffer without DCFDA. Fluorescence was monitored at 37 °C using the Fluoroskan (excitation: 495 nm, emission: 550 nm) every minute over a 1 h period to calculate the integrated fluorescence signal (RFU), which was normalized using the crystal violet OD values. Data were compared to the control condition and expressed as a percentage. Cells were pre-incubated with the nitrone mito-PBN (10 μM) for 1h, after which the medium was removed and the DCFDA assay was performed as detailed above.

#### 3.2.9. Measurement of Mitochondrial ROS Production

Cells were seeded in black 96-well plates (20,000 cells per well) and incubated in buffer alone or with different treatments for 4 h. The MitoSOX Red reagent was added at the optimal concentration of 1 µM [43] 7 min before the incubation finished. Experiments were then conducted according to manufacturer protocol (https://www.thermofisher.com/order/catalog/product/M36008#/M36008, accessed on 25 March 2025). Fluorescence was observed and recorded using fluorescence microscopy. MitoSox superoxide indicator has excitation/emission maxima of 396 and 610 nm, respectively. The results obtained were normalized using the crystal violet OD values. They were then compared to the control condition and expressed as a percentage.

### 3.3. Antioxidant Capacity Assays

#### 3.3.1. Chemicals

6-Hydroxy-2,5,7,8-tetramethylchroman-2-carboxylic acid (Trolox), allopurinol, 3,4,5-trimethoxybenzoic acid, 3-hydroxybenzoic acid, 4-hydroxybenzoic acid, and 3,4-dihydroxybenzoic acid were from Thermo Fischer Scientific. 2,2′-azobis(2-methylpropioamidine) dihydrochloride (AAPH), Phosphate-Buffered Saline (PBS), caffeic acid (3-(3,4-dihydroxyphenyl)prop-2-enoic acid), ferulic acid (3-(4-hydroxy-3-methoxyphenyl)prop-2-enoic acid), sinapic acid (3-(4-hydroxy-3,5-dimethoxyphenyl)prop-2-enoic acid), salicylic acid (2-hydroxybenzoic acid) and gallic acid (3,4,5-trihydroxybenzoic acid), glycine buffer, xanthine, xanthine oxidase (from buttermilk), 1,1′-diphenyl-2-picrylhydrazyl (DPPH), lucigenin (*N*,*N*-dimethyl-9,9-biacridinium dinitrate) and luminol (5-amino-2,3-dihydro-phthalazine-1,4-dione), and fluorescein were from Sigma-Aldrich (Saint Quentin Fallavier, France).

#### 3.3.2. DPPH Assay

DPPH radical reducing activity was determined according to a reported procedure [49]. Aliquots of test compound dissolved in ethanol (20–1000 µM, final concentration) were mixed with 150 µL of a fresh DPPH solution (0.19 mM) in ethanol, and the final volume was adjusted to 225 µL. After stirring the mixtures for 3 or 30 min at room temperature in the dark, the absorbance was monitored at 517 nm on a microplate reader (TECAN Infinite M200, Tecan France S.A.S.U., Lyon, France). Percent DPPH scavenged was calculated using Equation (1):

Inhibition (%) = [100 × (*A*_0_ − *A*)/*A*_0_] (1)

where *A*_0_ is the absorbance value of the blank and *A* is the absorbance in the presence of a given concentration of the tested compound. IC_50_ values (the concentration resulting in 50% DPPH scavenging) were calculated (in µM) through the non-linear fitting of dose–response curves.

#### 3.3.3. ORAC Assay

The assay was performed in microplates according to Kandouli et al. [49]. A fluorescein stock solution (821 µM) was prepared in 1X PBS and stored at 4 °C. Solutions of AAPH (153 mM) and fluorescein (82.1 nM) in KH_2_PO_4_ buffer (100 mM, pH 7.4) were prepared daily. Samples (25 μL/well) were mixed with 150 μL/well of fluorescein solution, and the mixture was incubated for 10 min at 37 °C. Aliquots of AAPH solution (25 μL/well, 19.12 mM, final concentration) were added, and the fluorescence (excitation: 485 nm, emission: 530 nm) was recorded every 2 min for 70 min. The blank was made using KH_2_PO_4_ buffer instead of a test compound. In each assay, a calibration curve was performed with Trolox (3.125–100 μM) as a standard. The ORAC values were calculated using the neat areas under the curves (AUCs) and expressed as μmole Trolox Equivalents (TEs) per liter of solution.

#### 3.3.4. Superoxide Quenching

The O_2_^•−^ scavenging capacities were assayed in glycine buffer (6.25 mM, pH 10.1) at 37 °C according to a previous procedure [48] adapted for microplate measurement. The assay measures inhibition by the test compounds of lucigenin-derived chemiluminescence produced by the allopurinol/XO O_2_^•−^ generating system. The assay was performed line by line on the microplate (12 wells with 3 blank wells). Compounds (46.25–185 µL) dissolved in glycine buffer:DMSO (1000:1, *v*:*v*) were diluted in glycine buffer to reach a final volume of 185 µL per well. To these solutions were added 15 µL of allopurinol solution (375 µM) and 30 µL of lucigenin solution (500 µM) per well, both in glycine buffer. A total of 20 µL per well of XO solution (150 mU/mL) was added at the same time, and luminescence of the line was monitored at 37 °C at 15 s intervals for a total period of 10 min following XO addition. The O_2_^•−^ scavenging activity was calculated against controls (in the absence of compound) from AUCs. The results are expressed as IC_50_ values (in µM).

### 3.4. Analysis

#### 3.4.1. Statistical Analysis

Statistical analyses of the data were performed using GraphPad Prim 10 Software (La Jolla, CA, USA). The results are presented as mean ± SD for the indicated number of independent experiments made in triplicate. In biological experiments, normality was assessed using the Shapiro–Wilk test and homogeneity of variances using the Brown–Forsythe test. The evaluation of statistical significance was conducted using Kruskal–Wallis or Student’s *t*-test when appropriate, or one-way analysis of variance (ANOVA), followed, if significant (*p* < 0.05), by Dunnett’s multiple comparisons test. Differences between groups were considered significant when *p* < 0.05.

#### 3.4.2. PamGene Data Analysis

PamChip^®^ tyrosine and serine/threonine kinase activity data were processed and analyzed using the PamStation12 BioNavigator software (PamGene International B.V., ‘s-Hertogenbosch, The Netherlands). Raw signal intensities were background-corrected, log-transformed, and normalized to internal controls. For each kinase, relative activity (setStat) and specificity (pSpecificityScore) were calculated using PamGene’s internal statistical model based on peptide-wise correlation and permutation analysis. Only kinases with pSpecificityScore < 0.05 were considered significantly modulated. The normalized dataset was exported and further analyzed in R software (version 4.3.1). Volcano plots were generated using the ggplot2 4.0.1 package to visualize differential kinase activity under metformin, **5a**, and **5h** treatments in GBM9 and U87 cells.

## 4. Conclusions

In summary, we synthesized nine new metformin–antioxidant hybrid molecules bearing aromatic rings, variably substituted with hydroxy and methoxy groups, to combine the anticancer potential of metformin with the redox-modulating effects of phenolic compounds. These hybrids demonstrated a markedly improved cytotoxicity in glioblastoma cell lines and stem-like cells compared with metformin and their phenolic precursors, while maintaining a selectivity toward cancer cells. Although these cellular models are widely used in preclinical glioblastoma research, they present certain limitations, notably the absence of a complete tumor microenvironment. Future studies using 3D or in vivo models would be necessary to confirm the translational relevance of our findings. The study of redox state modulation indicated that hybrids **5a** and **5h** could induce cytotoxicity through a cytosolic pro-oxidant effect, triggering apoptosis mechanisms following DNA damage. These molecules also displayed an enhanced antioxidant capacity compared to the corresponding parents, thus preventing oxidative stress-induced tumor formation and/or progression. The mechanisms of action of the lead compounds **5a** and **5h** were explored using kinomic and Western blot analyses, revealing a profound reprogramming of kinase signaling. Both hybrids inhibited ERK1/2, CDK2, and JAK2, while simultaneously activating AMPKα and stress-related kinases, supporting a cytostatic and pro-apoptotic response. Altogether, these results suggest that the metformin–phenolic hybrids act through a dual mechanism involving metabolic stress and multi-kinase inhibition, including redox perturbation, leading to apoptosis, extending metformin’s anticancer action beyond its classical AMPK activation. However, further studies are needed to fully elucidate their direct molecular targets.

Comparable strategies based on metformin hybridization have shown that integrating bioactive motifs can enhance anti-proliferative activity through concurrent redox and metabolic modulation [57]. Along with our findings, these observations highlighted the therapeutic potential of multifunctional metformin-based hybrids acting on convergent metabolic and signaling pathways.

Overall, the newly developed metformin–phenolic hybrids, particularly compounds 5a and 5h, emerged as promising bifunctional candidates for glioblastoma treatment by simultaneously targeting redox balance, metabolic stress, and kinase signaling regulation.

## Figures and Tables

**Table 1:** Effect of metformin hybrid molecules and related compounds on cytotoxicity against human glioma cell lines (U87 and U251), stem cells (GBM6 and GBM9), and human dermal fibroblasts (HDFs) ^a^.

Compound	MTT IC_50_ (µM) ^b^				
	U87	U251	GBM6	GBM9	HDFs
**5a**	27.3 ± 0.5	4.8 ± 1.1	11.8 ± 2.2	9.7 ± 2.5	>500
**5b**	68.3 ± 9.6	8.8 ± 1.1	34.7 ± 7.6	216.7 ± 35.3	322.3 ± 39.7
**5c**	127.4 ± 13.0	97.7 ± 10.4	122.7 ± 7.0	285.3 ± 14.7	375.0 ± 13.0
**5d**	>500 ^c^	62.0 ± 3.6	nd ^d^	nd	>500
**5e**	>500	nd	nd	nd	>500
**5f**	239.4 ± 27.2	258.0 ± 38.6	nd	nd	>500
**5g**	>500	nd	nd	nd	>500
**5h**	46.3 ± 2.3	55.3 ± 1.1	40.3 ± 0.6	18.7 ± 4.0	199.0 ± 6.6
**10**	>500	nd	nd	nd	>500
Metformin	6300 ± 1280	4100 ± 300	16,067 ± 116	11,000 ± 1400	40,800 ± 4087
Temozolomide	200 ± 9.6	nd	nd	nd	>500
Caffeic acid	>500	nd	nd	nd	nd
Ferulic acid	>500	nd	nd	nd	nd
Sinapic acid	>500	nd	nd	nd	nd
Salicylic acid	>500	nd	nd	nd	nd
Gallic acid	>500	nd	nd	nd	nd

^a^ Cells were exposed to increasing concentrations of compounds (from 0 to 500 µM for hybrids and phenolic compounds and 0 to 20 mM for metformin) for 72 h in the appropriate medium + 0.1% DMSO (vehicle). Data are means ± SD of 3–6 independent experiments made in triplicate. ^b^ IC_50_ (µM), defined as the concentration of compound resulting in 50% cell viability after 72 h and calculated from concentration–response curves. ^c^ >500 indicates that 50% inhibition of cell viability was not reached at 500 µM. ^d^ nd = not determined.

**Table 2:** Scavenging activity ^a^ for hybrid molecules compared to corresponding phenolic acids and Trolox.

Compound	DPPH 3 min EC_50_^b^ (µM)	DPPH 30 min EC_50_ (µM)
Hybrid molecules		
**5a**	122.5 ± 12.6	42.7 ± 2.8
**5b**	119.0 ± 1.7	47.7 ± 4.0
**5c**	115.7 ± 6.7	47.6 ± 4.1
**5d**	>200	>200
**5e**	147.7 ± 0.6	99.7 ± 4.9
**5f**	120.7 ± 1.2	71.0 ± 5.3
**5g**	54.0 ± 3.6	36.0 ± 1.7
**5h**	195.0 ±10.0	67.4 ± 6.7
**10**	>200	129.0 ± 9.6
Standard antioxidants		
Salicylic acid	>200	>200
3-hydroxybenzoic acid	>200	>200
4-hydroxybenzoic acid	>200	>200
3,4-dihydroxybenzoic acid	>200	>200
Caffeic acid	39.0 ± 1.0	29.7 ± 2.1
Ferrulic acid	>200	122.3 ± 10.8
Sinapic acid	57.7 ± 3.8	47.3 ± 2.5
3,4,5-trimethoxybenzoic acid	>200	>200
Resveratrol	118.1 ± 1.5	89.0 ± 4.3
Metformin	>500	>500
Trolox	35.0 ± 2.0	31.5 ± 0.7

^a^ Data are means ± SD of 3–6 independent experiments made in triplicate. ^b^ EC_50_ defined as the concentration of compound resulting in 50% decrease after 3 or 30 min incubation and calculated from concentration–response curves.

## Data Availability

The original contributions presented in this study are included in the article/Supplementary Materials. Further inquiries can be directed to the corresponding author.

## Supplementary Materials

The following supporting information can be downloaded at https://www.mdpi.com/article/10.3390/ijms27031259/s1. References [21,22,58,59,60,61,62,63,64,65,66,67,68] are cited in the supplementary materials.
