# Supplementary material for: Bifunctional Metformin–Phenolic Hybrids with Improved Anticancer and Antioxidant Properties: Evaluation on Glioma Cells
Source: Int J Mol Sci. 2026 Jan 27;27(3):1259. doi: 10.3390/ijms27031259 (PMC12898376; doi:10.3390/ijms27031259)
Supplement: Supplementary file 1 [file ijms-27-01259-s001.zip › ijms-4080784-supplementary.pdf]

Supplementary Materials

Title

Bifunctional Metformin-Phenolics Hybrids with Improved Anticancer and Antioxidant Properties: Evaluation on Glioma Cells

Authors

Caroline Delehedde [1†], Mathieu Chocry [1,2†], Camille Nguyen [1†*], Alice Asteian [1], Maxime Robin [1], Ludovic Leloup [2], Mathieu Cassien [1,3], Anne Mercier [1], Marcel Culcasi [1], Hervé Kovacic [2] and Sylvia Pietri [1,*]

[†] These authors contributed equally to this work

Addresses

1-Aix Marseille Univ, CNRS, Institut de Chimie Radicalaire (ICR), UMR 7273, Marseille, France

2- Aix Marseille Univ, CNRS, Institut de Neurophysiopathologie (INP), UMR 7051, Marseille, France

3- Yelen Analytics, Ensuès–la-Redonne, France

*Correspondence: sylvia.pietri@univ-amu.fr

Co-correspondence: camille.nguyen@univ-amu.fr

Table of contents

1. Synthetic procedures and characterization 4
   1.1 General procedure for the synthesis of **1a–g** 4
      1.1.1 2-Isoproproxybenzaldehyde **1a** 4
      1.1.2 3-Isoproproxybenzaldehyde **1b** 4
      1.1.3 4-Isoproproxybenzaldehyde **1c** 4
      1.1.4 3,5-Diisopropoxybenzaldehyde **1d** 4
      1.1.5 3,4-Diisopropoxybenzaldehyde **1e** 4
      1.1.6 4-Isopropoxy-3-methoxybenzaldehyde **1f** 5
      1.1.7 4-Isopropoxy-3,5-dimethoxybenzaldehyde **1g** 5
   1.2 General procedure for the synthesis of **2a–h** 5

1.2.1 (*E*)-3-(2-Isopropoxyphenyl)acrylic acid **2a** 5
1.2.2 (*E*)-3-(3-Isopropoxyphenyl)acrylic acid **2b** 5
1.2.3 (*E*)-3-(4-Isopropoxyphenyl)acrylic acid **2c** 6
1.2.4 (*E*)-3-(3,5-Diisopropoxyphenyl)acrylic acid **2d** 6
1.2.5 (*E*)-3-(3,4-Diisopropoxyphenyl)acrylic acid **2e** 6
1.2.6 (*E*)-3-(4-Isopropoxy-3-methoxyphenyl)acrylic acid **2f** 6
1.2.7 (*E*)-3-(4-Isopropoxy-3,5-dimethoxyphenyl)acrylic acid **2g** 6
1.2.8 (*E*)-3-(3,4,5-Trimethoxyphenyl)acrylic acid **2h** 7
2. NMR spectra (^{1}H and ^{13}C) of metformin-phenolic acid hybrids and precursors 8
2.1. Spectra of **3a** 8
2.2. Spectra of **3b** 9
2.3. Spectra of **3c** 10
2.4. Spectra of **3d** 11
2.5. Spectra of **3e** 12
2.6. Spectra of **3f** 13
2.7. Spectra of **3g** 14
2.8. Spectra of **3h** 15
2.9. Spectra of **4a** 16
2.10. Spectra of **4b** 17
2.11. Spectra of **4c** 18
2.12. Spectra of **4d** 19
2.13. Spectra of **4e** 20
2.14. Spectra of **4f** 21
2.15. Spectra of **4g** 22
2.16. Spectra of **5a** 23
2.17. Spectra of **5b** 24
2.18. Spectra of **5c** 25
2.19. Spectra of **5d** 26
2.20. Spectra of **5e** 27
2.21. Spectra of **5f** 28
2.22. Spectra of **5g** 29
2.23. Spectra of **5h** 30
3. Procedure for the synthesis of compounds **6-7** 31
3.1 2-(4-Isopropoxyphenyl)acetic acid (**6**) 31
3.2 (*E*)-3-(3,5-Diisopropoxyphenyl)-2-(4-isopropoxyphenyl)acrylic acid (**7**) 31
4. NMR spectra (^{1}H and ^{13}C) of metformin-resveratrol hybrids 32
4.1 Spectra of **8** 32

4.2 Spectra of **9** 33

4.3 Spectra of **10** 34

5. Calculated AlogP values for metformin, phenolic acids and hybrid derivatives (**Table S1**) ……………………………………………………………………………35

6. Impact of a 4 h treatment with metformin, **5a** or **5h** on serine threonine kinase (STK) and tyrosine kinase (PTK) activity as compared to control (DMSO 0,01%) using kinomic Pamgene assay.. 35

6.1 List of kinases significantly inhibited or activated by metformin in GBM9 and U87 cells (**Table S2**) 35

6.2 List of kinases significantly inhibited or activated by **5a** in GBM9 and U87 cells (**Table S3**)... 38

6.3 List of kinases significantly inhibited or activated by **5h** in GBM9 and U87 cells (**Table S4**) .. 42

7. Effect of DMSO on Akt, ERK1/2 and AMPKα phosphorylation on U87 cells (**Figure S1**) ……………………………………………………………………………..45

8. Original, uncropped Western blot images (**Figure S2-10**)…………..………………46

8.1 Western blot source images for Akt (**Figure S2**)……………………………………………46

8.2 Western blot source images for P-Akt (**Figure S3**) …………………………………………47

8.3 Western blot source images for GAPDH (loading control for Akt and P-Akt) (**Figure S4)**…...48

8.4 Western blot source images for AMPK (**Figure S5**) ………………………………………..49

8.5 Western blot source images for P-AMPK (**Figure S6**) …………………………………… 50

8.6 Western blot source images for GAPDH (loading control for AMPK and P-AMPK) (**Figure S7)** ……………………………………………………………………………………………51

8.7 Western blot source images for ERK1/2 (**Figure S8**) ……………………………………...52

8.8 Western blot source images for P-ERK1/2 (**Figure S9**) …………………………………...53

8.9 Western blot source images for GAPDH (loading control for ERK1/2 and P-ERK1/2) (**Figure S10)** ……………………………………………………………………………………………54

References 55

1. Synthetic procedures and characterization

1.1 General procedure for the synthesis of **1a–g**

To the corresponding hydroxylated benzaldehyde dissolved in DMF (1.8 M, final concentration) were successively added K_2CO_3 (2.2 eq for each OH group) and 2-bromopropane (2.2 eq / OH group to protect) and the mixture was heated at 55 °C for 16 h. After cooling down to room temperature followed by addition of ethyl acetate, the mixture was washed with water and the organic layer was dried over $MgSO_4$, filtered and concentrated to afford the desired compound used without further purification.

1.1.1 2-Isoproproxybenzaldehyde **1a**

Title compound was obtained from 2-hydroxybenzaldehyde (3.2 mL, 41 mmol), K_2CO_3 (12.4 g, 90.2 mmol) and 2-bromopropane (8.5 mL, 90.2 mmol). Yellow pale oil (5 g, 80%). ^{1}H NMR (300 MHz, DMSO-d_6) δ 10.37 (s, 1H, CHO), 7.67 (dd, 1H, J = 7.7 Hz and 1.8 Hz, H-6), 7.58 (td, 1H, J = 7.5 Hz and 2.0 Hz, H-4), 7.17 (bd, 1H, J = 8.4 Hz, H-5), 7.00 (bt, 1H, J = 7.7 Hz, H-3), 4.73 (sept, 1H, J = 6.1 Hz, C*H*(CH_3)$_2$), 1.30 (d, 6H, J = 6.1 Hz, CH(C*H*$_3$)$_2$); ^{13}C NMR (75 MHz, DMSO-d_6) δ 189.7 (C=O), 160.6 (C-2), 136.6 (C-4), 128.0 (C-6), 125.5 (C-1), 120.8 (C-5), 115.3 (C-3), 71.2 (*C*H(CH_3)$_2$), 22.0 (CH(*C*H$_3$)$_2$). HRMS calc. for $C_{10}H_{13}O_2^+$ [M+H]$^+$ 165.0910, found 165.0911. Data in accordance with the literature [1].

1.1.2 3-Isoproproxybenzaldehyde **1b**

Title compound was obtained from 3-hydroxybenzaldehyde (3 g, 25 mmol), K_2CO_3 (7.6 g, 55 mmol) and 2-bromopropane (5 mL, 55 mmol). Colorless oil (4.1 g, quant. yield). ^{1}H NMR (300 MHz, $CDCl_3$) δ 9.81 (s, 1H, CHO), 7.29 (bs, 1H, H-6), 7.26 (bs, 1H, H-2), 7.22 (bd, 1H, J = 2.5 Hz, H-4), 7.00 (m, 1H, H-5), 4.48 (sept, 1H, J = 6.1 Hz, C*H*(CH_3)$_2$), 1.22 (d, 6H, J = 6.1 Hz, CH(C*H*$_3$)$_2$); ^{13}C NMR (75 MHz, $CDCl_3$) δ 192.2 (C=O), 158.7 (C-3), 138.0 (C-1), 130.2 (C-5), 123.1 (C-4, C-6), 114.4 (C-2), 70.4 (*C*H(CH_3)2), 22.0 (CH(*C*H$_3$)$_2$). HRMS calc. for $C_{10}H_{13}O_2^+$ [M+H]$^+$ 165.0910, found 165.0913. Data in accordance with the literature [2].

1.1.3 4-Isoproproxybenzaldehyde **1c**

Title compound was obtained from 4-hydroxybenzaldehyde (3 g, 25 mmol), K_2CO_3 (7.6 g, 55 mmol) and 2-bromopropane (5 mL, 55 mmol). Colorless oil (4.1 g, quant. yield). ^{1}H NMR (300 MHz, $CDCl_3$) δ 9.86 (s, 1H, CHO), 7.79 (d, 2H, J = 8.4 Hz, H-2), 6.95 (d, 2H, J = 8.4 Hz, H-3), 4.65 (sept, 1H, J = 6.2 Hz, C*H*(CH_3)$_2$), 1.36 (d, 6H, J = 6.2 Hz, CH(C*H*$_3$)$_2$); ^{13}C NMR (75 MHz, $CDCl_3$) δ 190.8 (C=O), 163.2 (C-4), 132.1 (C-2), 129.7 (C-1), 115.9 (C-3), 70.4 (*C*H(CH_3)$_2$), 22.1 (CH(*C*H$_3$)$_2$). HRMS calc. for $C_{10}H_{13}O_2^+$ [M+H]$^+$ 165.0910, found 165.0911. Data in accordance with the literature [3].

1.1.4 3,5-Diisopropoxybenzaldehyde **1d**

Title compound was obtained from 3,5-dihydroxybenzaldehyde (2 g, 14.5 mmol), K_2CO_3 (8.8 g, 63.8 mmol, 4.4 eq) and 2-bromopropane (6.0 mL, 63.8 mmol, 4.4 eq). Red oil (3.2 g, quant. yield). ^{1}H NMR (300 MHz, $CDCl_3$) δ 9.86 (s, 1H, CHO), 6.94 (s, 2H, H-2), 6.65 (s, 1H, H-4), 4.57 (sept, 2H, J = 6.0 Hz, 2 × C*H*(CH_3)$_2$), 1.33 (d, 12H, J = 6.0 Hz, 2 × CH(C*H*$_3$)$_2$); ^{13}C NMR (75 MHz, $CDCl_3$) δ 191.9 (C=O), 159.5 (C-3), 138.3 (C-1), 110.2 (C-4), 108.5 (C-2), 70.2 (*C*H(CH_3)$_2$), 21.8 (CH(*C*H$_3$)$_2$). HRMS calc. for $C_{13}H_{19}O_3^+$ [M+H]$^+$ 223.1329, found 223.1332. Data in accordance with the literature [4].

1.1.5 3,4-Diisopropoxybenzaldehyde **1e**

Title compound was obtained from 3,4-dihydroxybenzaldehyde (5 g, 36.2 mmol), K_2CO_3 (22 g, 159 mmol) and 2-bromopropane (15 mL, 159 mmol). Yellow pale oil (6.4 g, 80%). ^{1}H NMR (300 MHz, $CDCl_3$) δ 9.83 (s, 1H, CHO), 7.46-7.43 (m, 2H, H-2 and H-3), 6.99 (d, 1H,

J = 8.8 Hz, H-5), 4.64 (sept, 1H, *J* = 6.2 Hz, C*H*(CH_3)$_2$), 4.53 (sept, 1H, *J* = 6.2 Hz, C*H*(CH_3)$_2$), 1.40 (d, 6H, *J* = 6.2 Hz, CH(C*H*$_3$)$_2$), 1.36 (d, 6H, *J* = 6.2 Hz, CH(C*H*$_3$)$_2$); ^{13}C NMR (75 MHz, $CDCl_3$) δ 190.9 (C=O), 155.0 (C-4), 149.0 (C-3), 130.1 (C-1), 126.4 (C-6), 116.1 (C-2), 114.9 (C-5), 72.4 (*C*H(CH_3)$_2$), 71.8 (*C*H(CH_3)$_2$), 22.1 (CH(*C*H$_3$)$_2$), 22.0 (CH(*C*H$_3$)$_2$). HRMS calc. for $C_{13}H_{19}O_3^+$ $[M+H]^+$ 223.1329, found 223.1329. Data in accordance with the literature [5].

1.1.6 4-Isopropoxy-3-methoxybenzaldehyde **1f**

Title compound was obtained from 4-hydroxy-3-methoxybenzaldehyde (5 g, 32.9 mmol), K_2CO_3 (10 g, 72.5 mmol) and 2-bromopropane (6.9 mL, 72.5 mmol). Yellow pale oil (5.8 g, 91%). ^{1}H NMR (300 MHz, $CDCl_3$) δ 9.83 (s, 1H, CHO), 7.42 (dd, 1H, *J* = 7.9 Hz and *J* = 1.8 Hz, H-6), 7.40 (bs, 1H, H-2), 6.97 (d, 1H, *J* = 7.9 Hz, H-5), 4.68 (sept, 1H, *J* = 6.1 Hz, C*H*(CH_3)$_2$), 3.91 (s, 3H, OCH_3), 1.42 (d, 6H, *J* = 6.1 Hz, CH(C*H*$_3$)$_2$); ^{13}C NMR (75 MHz, $CDCl_3$) δ 190.8 (C=O), 153.1 (C-3), 150.4 (C-4), 129.8.1 (C-1), 126.6 (C-6), 112.9 (C-5), 109.6 (C-2), 71.3 (*C*H(CH_3)$_2$), 56.0 (OCH_3), 21.9 (CH(*C*H$_3$)$_2$). HRMS calc. for $C_{11}H_{15}O_3^+$ $[M+H]^+$ 195.1016, found 195.1014. Data in accordance with the literature [6].

1.1.7 4-Isopropoxy-3,5-dimethoxybenzaldehyde **1g**

Title compound was obtained from 4-hydroxy-3,5-dimethoxybenzaldehyde (5 g, 27.5 mmol), K_2CO_3 (8.3 g, 60.5 mmol) and 2-bromopropane (5.7 mL, 60.5 mmol). Yellow pale oil (4.9 g, 81%). ^{1}H NMR (300 MHz, $CDCl_3$) δ 9.88 (s, 1H, CHO), 7.14 (s, 2H, H-2), 4.55 (sept, 1H, *J* = 6.2 Hz, C*H*(CH_3)$_2$), 3.92 (s, 6H, 2 × OCH_3), 1.33 (d, 6H, *J* = 6.2 Hz, CH(C*H*$_3$)$_2$); ^{13}C NMR (75 MHz, $CDCl_3$) δ 191.2 (C=O), 154.3 (C-3), 142.0 (C-4), 131.5 (C-1), 106.7 (C-2), 76.0(*C*H(CH_3)$_2$), 56.2 (OCH_3), 22.5 (CH(*C*H$_3$)$_2$). HRMS calc. for $C_{12}H_{17}O_4^+$ $[M+H]^+$ 225.1121, found 225.1124. Data in accordance with the literature [7].

1.2 General procedure for the synthesis of **2a–h**

To the benzaldehyde derivative **1a−g**, dissolved in glacial acetic acid (0.5 M, final concentration) at room temperature was successively added malonic acid (2.2 eq) and piperidine (2.7 eq) dropwise and the mixture was heated at 120 °C for 1 day. The reaction mixture was allowed to cool at room temperature, poured onto a water/ice mixture and the precipitate was filtered off, washed with water and dried to afford the desired compound used without further purification.

1.2.1 (*E*)-3-(2-Isopropoxyphenyl)acrylic acid **2a**

Title compound was obtained from **1a** (5.0 g, 30.5 mmol), malonic acid (7.0 g, 67.1 mmol) and piperidine (8.1 mL, 82.3 mmol). Beige powder (6.45 g, 95%), mp 112.4 °C. ^{1}H NMR (300 MHz, DMSO-d_6) δ 7.83 (d, 1H, *J* = 16.1 Hz, Ar-C*H*=CH), 7.64 (d, 1H, *J* = 7.7 Hz, H-6), 7.34 (t, 1H, *J* = 7.7 Hz, H-5), 7.05 (d, 1H, *J* = 8.3 Hz, H-3), 6.93 (t, 1H, *J* = 7.5 Hz, H-4), 6.50 (d, 1H, *J* = 16.1 Hz, Ar-CH=C*H*), 4.66 (sept, 1H, *J* = 5.9 Hz, C*H*(CH_3)$_2$), 1.28 (d, 6H, *J* = 5.9 Hz, CH(C*H*$_3$)$_2$); ^{13}C NMR (75 MHz, DMSO-d_6) δ 168.2 (C=O), 156.3 (C-2), 139.2 (Ar-*C*H=CH), 131.8 (C-4), 128.8 (C-6), 123.5 (C-1), 120.7 (Ar-CH=*C*H), 119.2 (C-1), 114.2 (C-3), 70.4 (*C*H(CH_3)$_2$), 21.9 (CH(*C*H$_3$)$_2$). HRMS calc. for $C_{12}H_{15}O_3^+$ $[M+H]^+$ 207.1016, found 207.1017. Data in accordance with the literature [8].

1.2.2 (*E*)-3-(3-Isopropoxyphenyl)acrylic acid **2b**

Title compound was obtained from **2b** (4.1 g, 25 mmol), malonic acid (5.70 g, 55 mmol) and piperidine (6.71 mL, 68.0 mmol). Beige powder (4.86 g, 94%), mp 93.3–94.3 °C. ^{1}H NMR (300 MHz, $CDCl_3$) δ 7.69 (d, 1H, *J* = 15.8 Hz, Ar-C*H*=CH), 7.28 (t, 1H, *J* = 8.1 Hz, H-5), 7.10 (bd, 1H, *J* = 7.7 Hz, H-6), 7.05 (d, 1H, *J* = 1.9 Hz, H-2), 6.92 (dd, 1H, *J* = 8.1 and 2.3 Hz, H-4), 6.43 (d, 1H, *J* = 15.8 Hz, Ar-CH=C*H*), 6.33 (bs, 1H, OH), 4.57 (sept, 1H, *J* = 6.3

Hz, C*H*(CH_3)$_2$), 1.36 (d, 6H, *J* = 6.3 Hz, CH(C*H*$_3$)$_2$); ^{13}C NMR (75 MHz, $CDCl_3$) δ 166.2 (C=O), 158.5 (C-3), 146.1 (Ar-*C*H=CH), 136.0 (C-1), 130.1 (C-5), 120.9 (C-6), 118.9 (Ar-CH=*C*H), 118.3 (C-4), 115.4 (C-2), 70.3 (*C*H(CH_3)2), 22.2 (CH(*C*H$_3$)$_2$). HRMS calc. for $C_{12}H_{13}O_3^-$ [M-H]$^-$ 205.0870, found 205.0868. Data in accordance with the literature [9].

1.2.3 (*E*)-3-(4-Isopropoxyphenyl)acrylic acid **2c**

Title compound was obtained from **1c** (4.1 g, 25 mmol), malonic acid (5.70 g, 55 mmol) and piperidine (6.71 mL, 68.0 mmol). Beige powder (4.30 g, 83%), mp 154.5 °C. ^{1}H NMR (300 MHz, $CDCl_3$) δ 9.16 (s, 1H, CO_2H), 7.70 (d, 1H, *J* = 15.9 Hz, Ar-C*H*=CH), 7.48 (d, 2H, *J* = 8.8 Hz, H-2), 6.89 (d, 2H, *J* = 8.6 Hz, H-3), 6.33 (d, 1H, *J* = 15.9 Hz, Ar-CH=C*H*), 4.60 (sept, 1H, *J* = 6.1 Hz, C*H*(CH_3)$_2$), 1.36 (d, 6H, *J* = 6.1 Hz, CH(C*H*$_3$)$_2$);^{13}C NMR (75 MHz, $CDCl_3$) δ 160.1 (C=O), 145.9 (C-4, Ar-*C*H=CH), 130.1 (C-2), 126.9 (C-1), 116.0 (C-3), 115.7(Ar-CH=*C*H), 70.2 (*C*H(CH_3)$_2$), 22.2 (CH(*C*H$_3$)$_2$). HRMS calc. for $C_{12}H_{13}O_3^-$ [M-H]$^-$ 205.0870, found 205.0868. Data in accordance with the literature [8].

1.2.4 (*E*)-3-(3,5-Diisopropoxyphenyl)acrylic acid **2d**

Title compound was obtained from **1d** (3.0 g, 13.5 mmol), malonic acid (3.1 g, 29.7 mmol) and piperidine (3.6 mL, 36.5 mmol). Beige powder (3.6 g, quant. yield), mp 115 °C (litt: 117–118 °C [10]). ^{1}H NMR (300 MHz, DMSO-*d*$_6$) δ 7.45 (d, 1H, *J* = 15.9 Hz, Ar-C*H*=CH), 6.73 (d, 2H, *J* = 2.2 Hz, H-2), 6.46 (d, 1H, *J* = 16.1 Hz, Ar-CH=C*H*), 6.43 (d, 1H, *J* = 2.2 Hz, H-4), 4.61 (sept, 2H, *J* = 6.0 Hz, 2 × C*H*(CH_3)$_2$), 1.22 (d, 12H, *J* = 6.1 Hz, 2 × CH(C*H*$_3$)$_2$); ^{13}C NMR (75 MHz, DMSO-*d*$_6$) δ 168.1 (C=O), 159.2 (C-3), 145.5 (Ar-*C*H=CH), 136.6 (C-1), 119.8 (Ar-CH=*C*H), 107.8 (C-2), 105.7 (C-4), 69.8 (*C*H(CH_3)$_2$), 22.1 (CH(*C*H$_3$)$_2$). HRMS calc. for $C_{15}H_{21}O_4^+$ [M+H]$^+$ 265.1434, found 265.1435.

1.2.5 (*E*)-3-(3,4-Diisopropoxyphenyl)acrylic acid **2e**

Title compound was obtained from **1e** (6.4 g, 28.8 mmol), malonic acid (6.6 g, 63.4 mmol) and piperidine (7.7 mL, 77.8 mmol). Beige powder (6.9 g, 91%), mp 115 °C (litt: 117-118 °C [10]). ^{1}H NMR (300 MHz, $CDCl_3$) δ 11.90 (bs, 1 H, CO_2H), 7.74 (d, 1H, *J* = 15.6 Hz, Ar-C*H*=CH), 7.13 (bs, 2H, H-2 and H-6), 6.92 (d, 1H, *J* = 8.6 Hz, H-5), 6.32 (d, 1H, *J* = 15.6 Hz, Ar-CH=C*H*), 4.58 (sept, 1H, *J* = 5.9 Hz, C*H*(CH_3)$_2$), 4.49 (sept, 1H, *J* = 5.9 Hz, C*H*(CH_3)$_2$), 1.37 (m, 12H, 2 × CH(C*H*$_3$)$_2$); ^{13}C NMR (75 MHz, $CDCl_3$) δ 172.9 (C=O), 151.7 (C-4), 148.7 (C-3), 147.0 (Ar-*C*H=CH), 127.3 (C-6), 123.7 (Ar-CH=*C*H), 117.6 (C-2), 116.2 (C-5), 115.0 (C-1), 72.7 (*C*H(CH_3)$_2$), 72.0 (*C*H(CH_3)$_2$), 22.2 (CH(*C*H$_3$)$_2$). HRMS calc. for $C_{15}H_{21}O_4^+$ [M+H]$^+$ 265.1434, found 265.1534.

1.2.6 (*E*)-3-(4-Isopropoxy-3-methoxyphenyl)acrylic acid **2f**

Title compound was obtained from **1f** (5.8 g, 30.0 mmol), malonic acid (6.9 g, 66.0 mmol) and piperidine (8.0 mL, 81.0 mmol). Beige powder (5.4 g, 77%), mp 151 °C. ^{1}H NMR (300 MHz, $CDCl_3$) δ 11.70 (bs, 1H, CO_2H), 7.75 (d, 1H, *J* = 15.8 Hz, Ar-C*H*=CH), 7.13 (d, 1H, *J* = 8.3 Hz, H-6), 7.10 (bs, 1H, H-2), 6.90 (d, 1H, *J* = 8.3 Hz, H-5), 6.33 (d, 1H, *J* = 15.8 Hz, Ar-CH=C*H*), 4.63 (sept, 1H, *J* = 6.1 Hz, C*H*(CH_3)$_2$), 3.91 (s, 3H, OCH_3), 1.42 (d, 6H, *J* = 6.1 Hz, CH(C*H*$_3$)$_2$); ^{13}C NMR (75 MHz, $CDCl_3$) δ 172.8 (C=O), 150.3 (C-4), 150.0 (C-3), 147.0 (Ar-*C*H=CH), 126.9 (C-1), 122.9 (C-6), 114.8 (Ar-CH=*C*H), 114.3 (C-5), 110.6 (C-2), 71.3 (*C*H(CH_3)$_2$), 55.8 (OCH_3), 21.9 (CH(*C*H$_3$)$_2$). HRMS calc. for $C_{13}H_{17}O_4^+$ [M+H]$^+$ 237.1121, found 237.1122. Data in accordance with the literature [8].

1.2.7 (*E*)-3-(4-Isopropoxy-3,5-dimethoxyphenyl)acrylic acid **2g**

Title compound was obtained from **1g** (1.9 g, 10.4 mmol), malonic acid (2.4 g, 23.0 mmol) and piperidine (2.80 mL, 28.1 mmol). Beige powder (1.6 g, 59%), mp 159 °C. ^{1}H NMR (300 MHz, $CDCl_3$) δ 11.55 (bs, 1H, CO_2H), 7.73 (d, 1H, *J* = 15.8 Hz, Ar-C*H*=CH), 6.79 (s, 2H, H-

2), 6.38 (d, 1H, J = 15.8 Hz, Ar-CH=C*H*), 4.46 (sept, 1H, J = 6.2 Hz, C*H*(CH_3)$_2$), 3.88 (s, 6H, 2 × OCH_3), 1.32 (d, 6H, J = 6.2 Hz, CH(C*H*$_3$)$_2$); ^{13}C NMR (75 MHz, $CDCl_3$) δ 172.6 (C=O), 154.1 (C-3), 147.20 (Ar-*C*H=CH), 138.7 (C-4), 129.2 (C-1), 116.3 (Ar-CH=*C*H), 105.6 (C-2), 75.8 (*C*H(CH_3)$_2$), 56.3 (OCH_3), 22.5 (CH(*C*H$_3$)$_2$). HRMS calc. for $C_{14}H_{19}O_5^+$ $[M+H]^+$ 267.1227, found 267.1227.

1.2.8 (*E*)-3-(3,4,5-Trimethoxyphenyl)acrylic acid **2h**

Title compound was obtained from commercial 3,4,5-trimethoxybenzaldehyde (450 mg, 2.74 mmol), malonic acid (628 mg, 6.03 mmol) and piperidine (730 µL, 7.40 mmol). Since no precipitate was directly obtained using the general procedure described above, the aqueous layer was extracted with ethyl acetate (3 × 100 mL), the organic layers were gathered, dried over $MgSO_4$ and concentrated under vacuum to yield a beige powder (560 mg, 99%), mp 126 °C (litt: 127–128 °C [8]). ^{1}H NMR (300 MHz, $CDCl_3$) δ 7.60 (d, 1H, J = 15.8 Hz, Ar-C*H*=CH), 6.71 (s, 2H, 2 × H-2), 6.27 (d, 1H, J = 15.8 Hz, Ar-CH=C*H*), 3.80 (s, 9H, 3 × OCH_3); ^{13}C NMR (75 MHz, $CDCl_3$) δ 172.1 (C=O), 153.2 (C-3), 146.8 (Ar-*C*H=CH), 140.3 (C-4), 129.4 (C-1), 116.2 (Ar-CH=*C*H), 105.5 (C-2), 55.9 (OCH_3). HRMS calc. for $C_{12}H_{15}O_5^+$ $[M+H]^+$ 239.0914, found 239.0913. Data in accordance with the literature [11].

2. NMR spectra (^{1}H and ^{13}C) of metformin-phenolic acid hybrids and precursors

2.1. Spectra of **3a**

^{1}H NMR (300 MHz, DMSO-d_6)

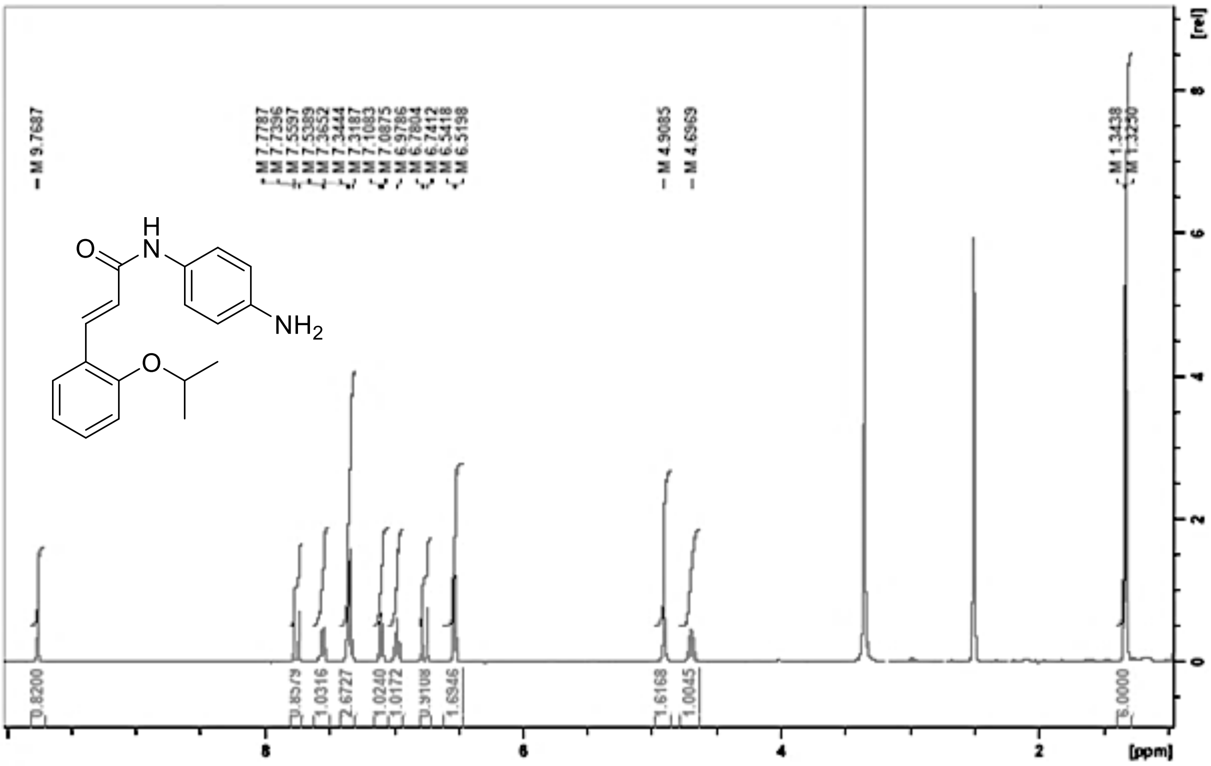

^{13}C NMR (75 MHz, DMSO-d_6)

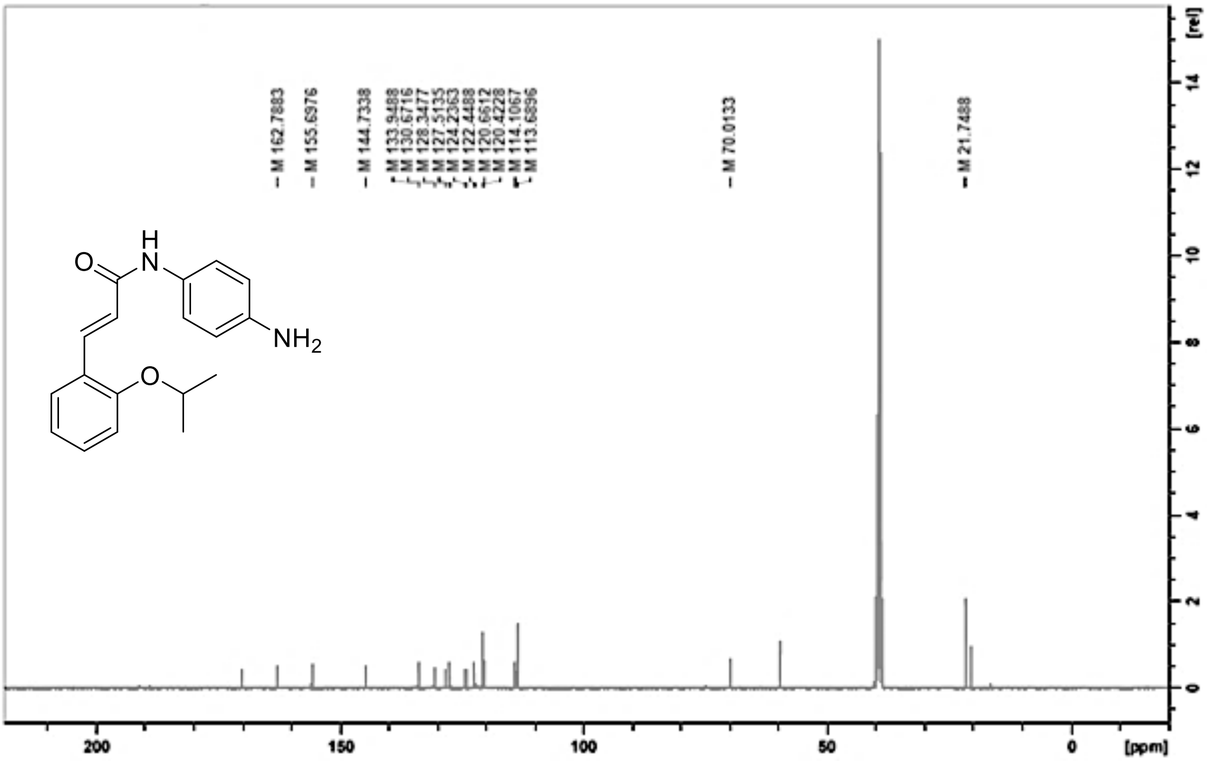

2.2. Spectra of **3b**

^{1}H NMR (300 MHz, DMSO-*d*$_6$)

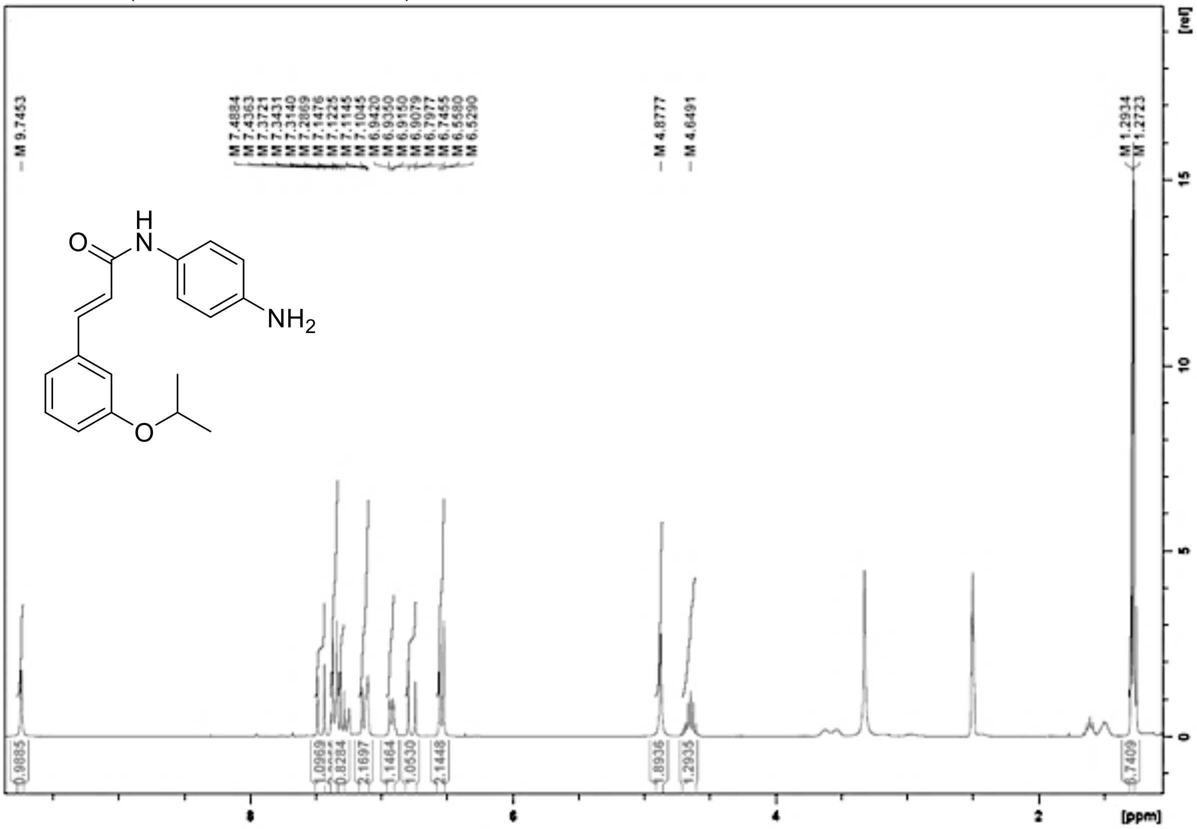

^{13}C NMR (75 MHz, DMSO-*d*$_6$)

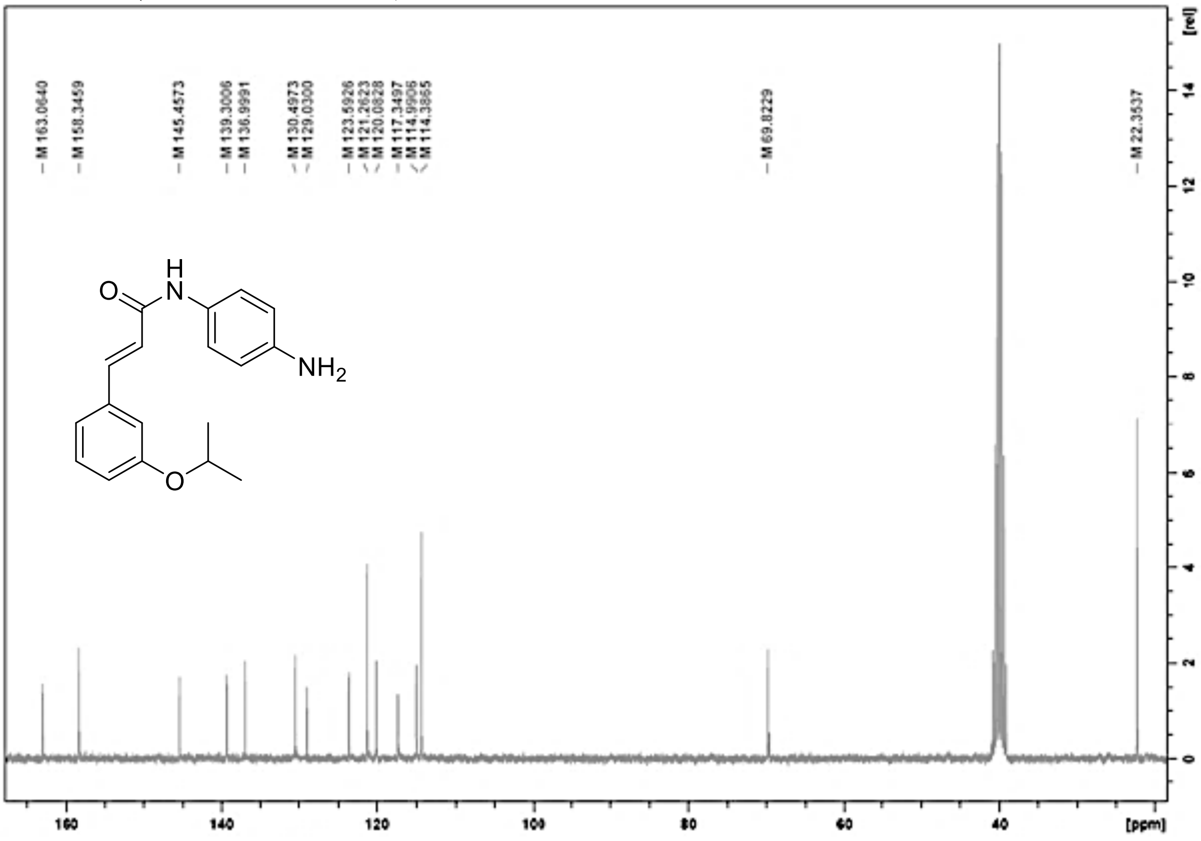

2.3. Spectra of **3c**

^{1}H NMR (300 MHz, DMSO-*d*$_6$)

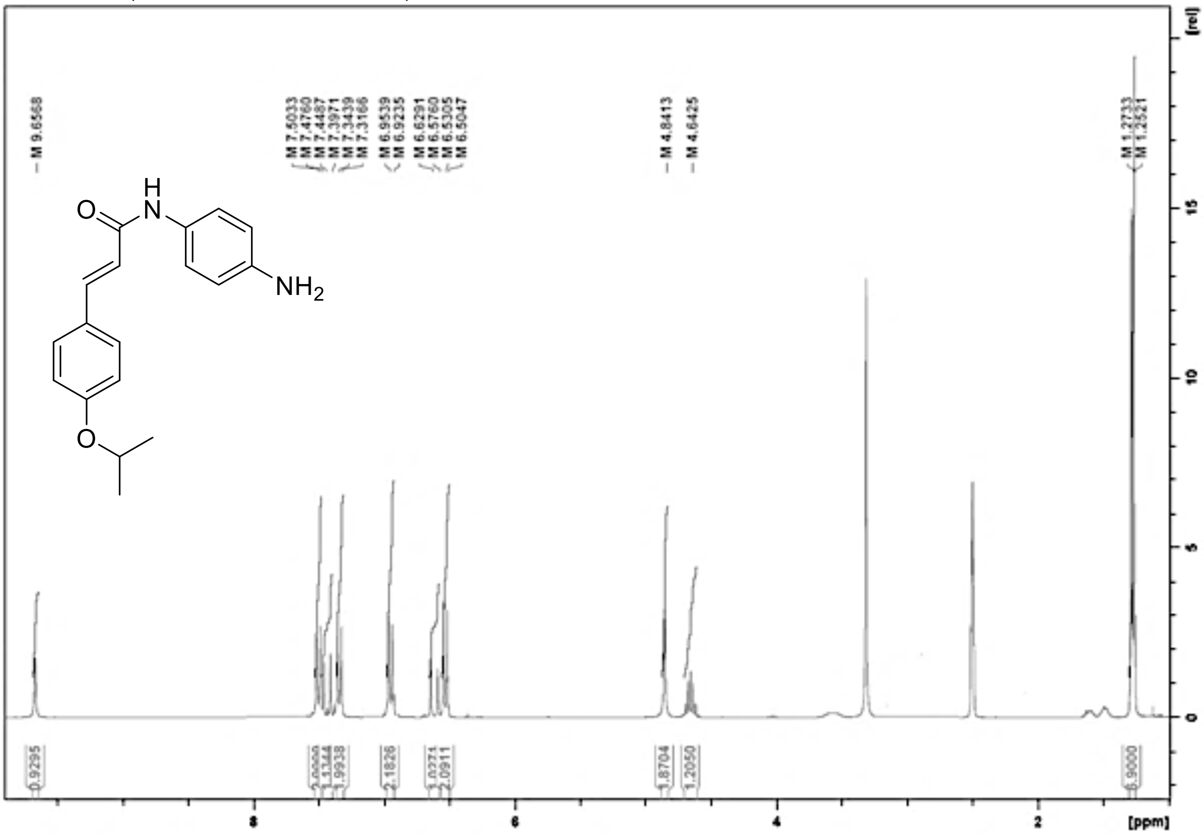

^{13}C NMR (75 MHz, DMSO-*d*$_6$)

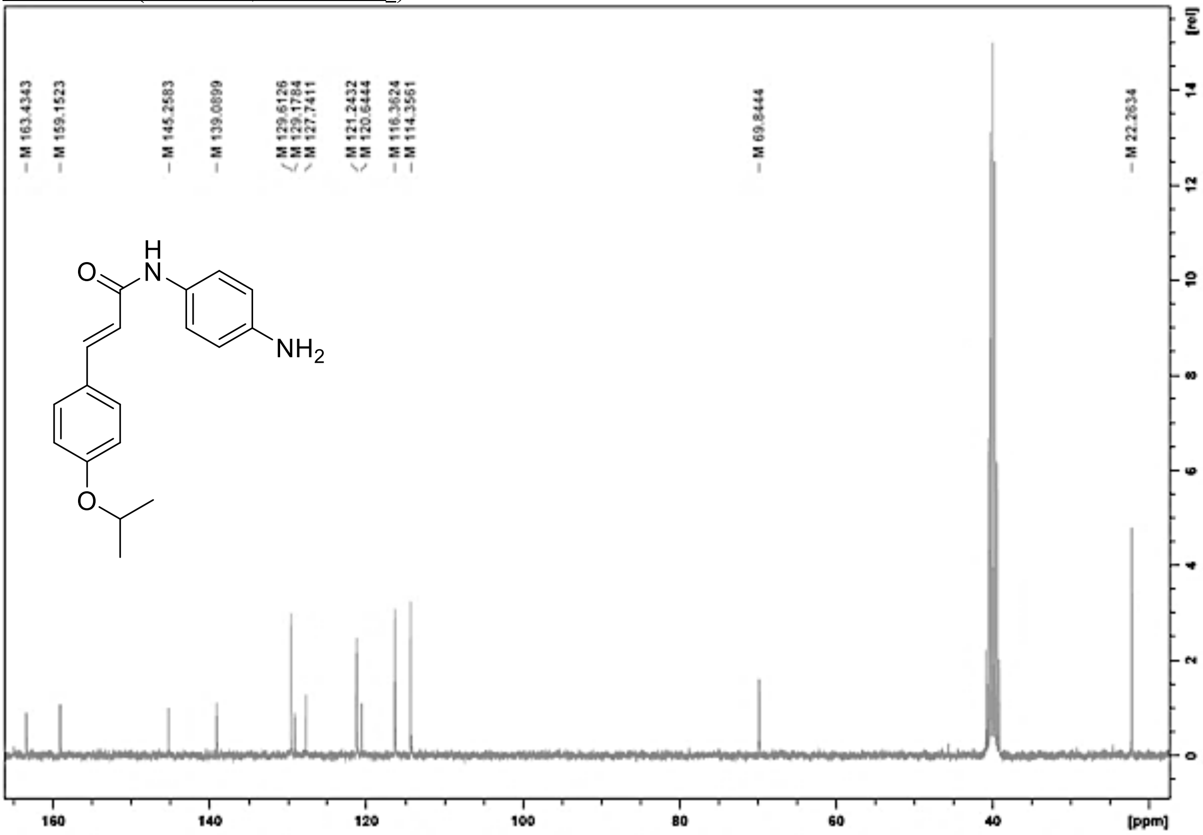

2.4. Spectra of **3d**

^{1}H NMR (400 MHz, DMSO-d_6)

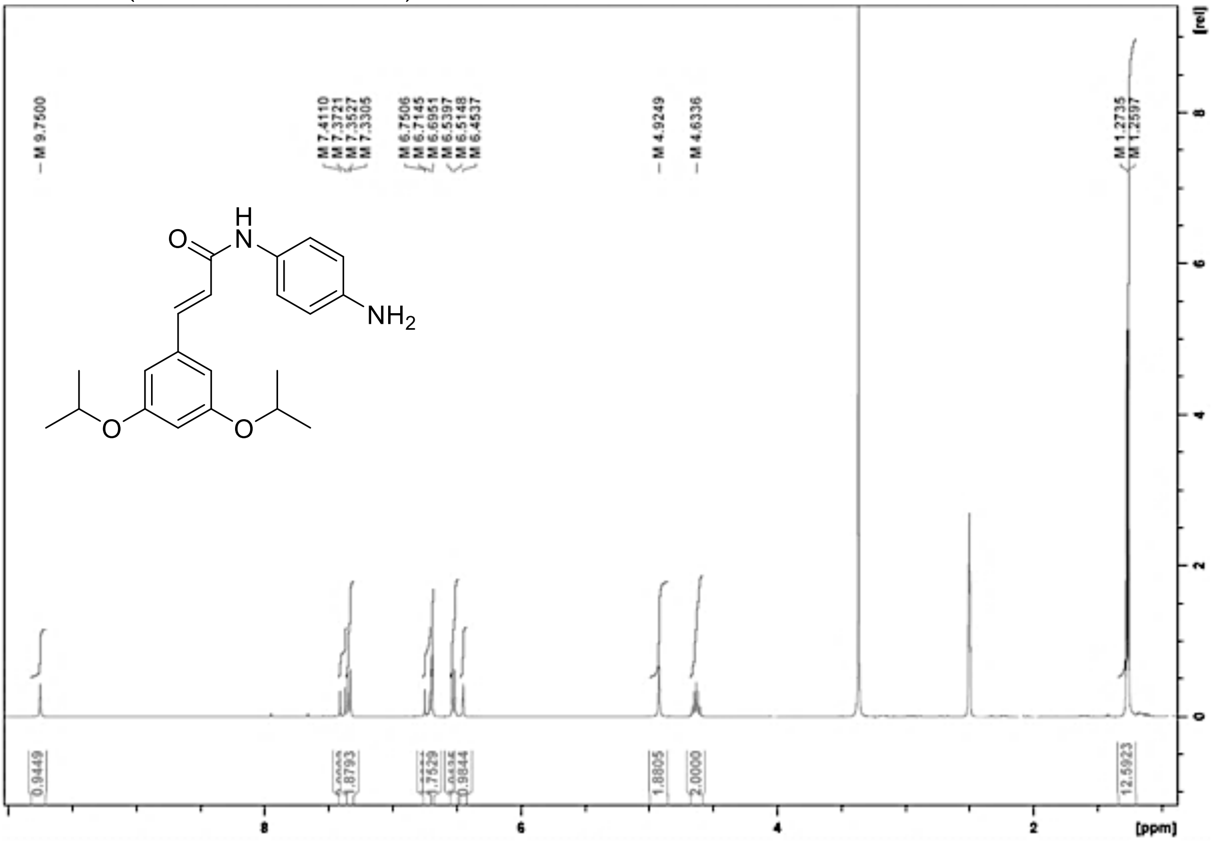

^{13}C NMR (100.6 MHz, DMSO-d_6)

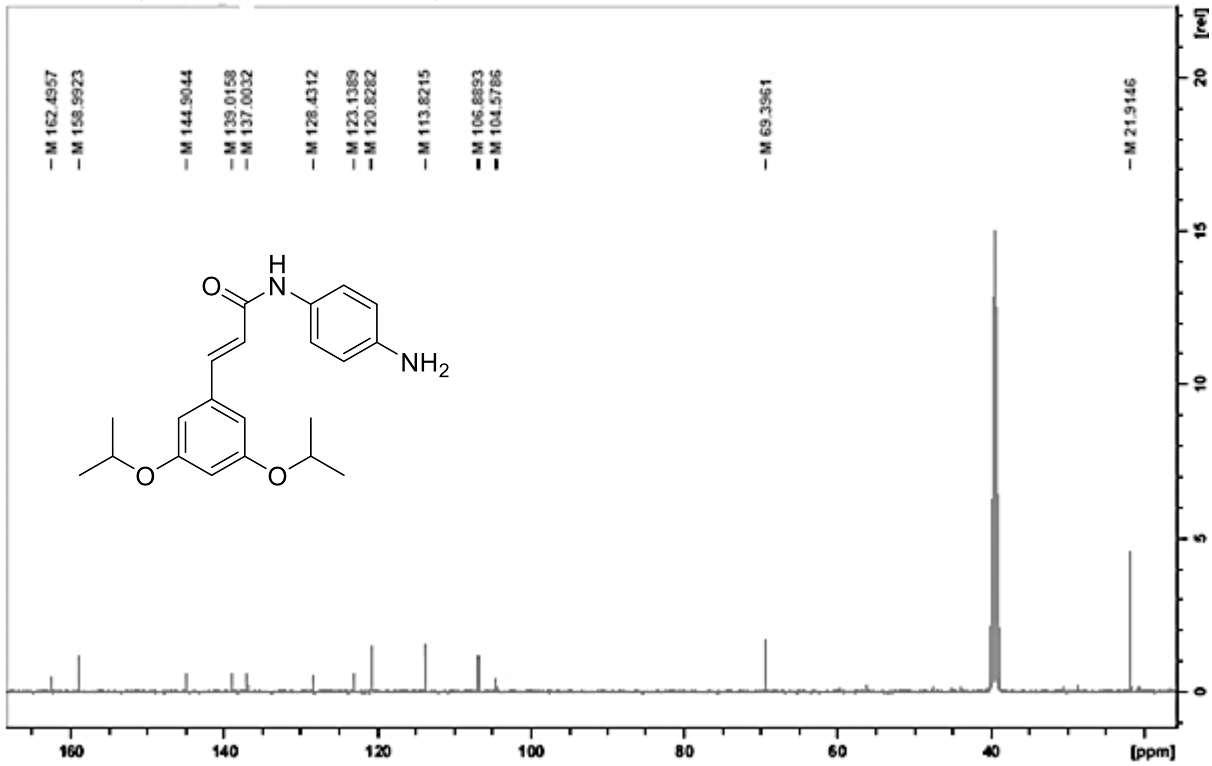

2.5. Spectra of **3e**

^{1}H NMR (400 MHz, DMSO-d_6)

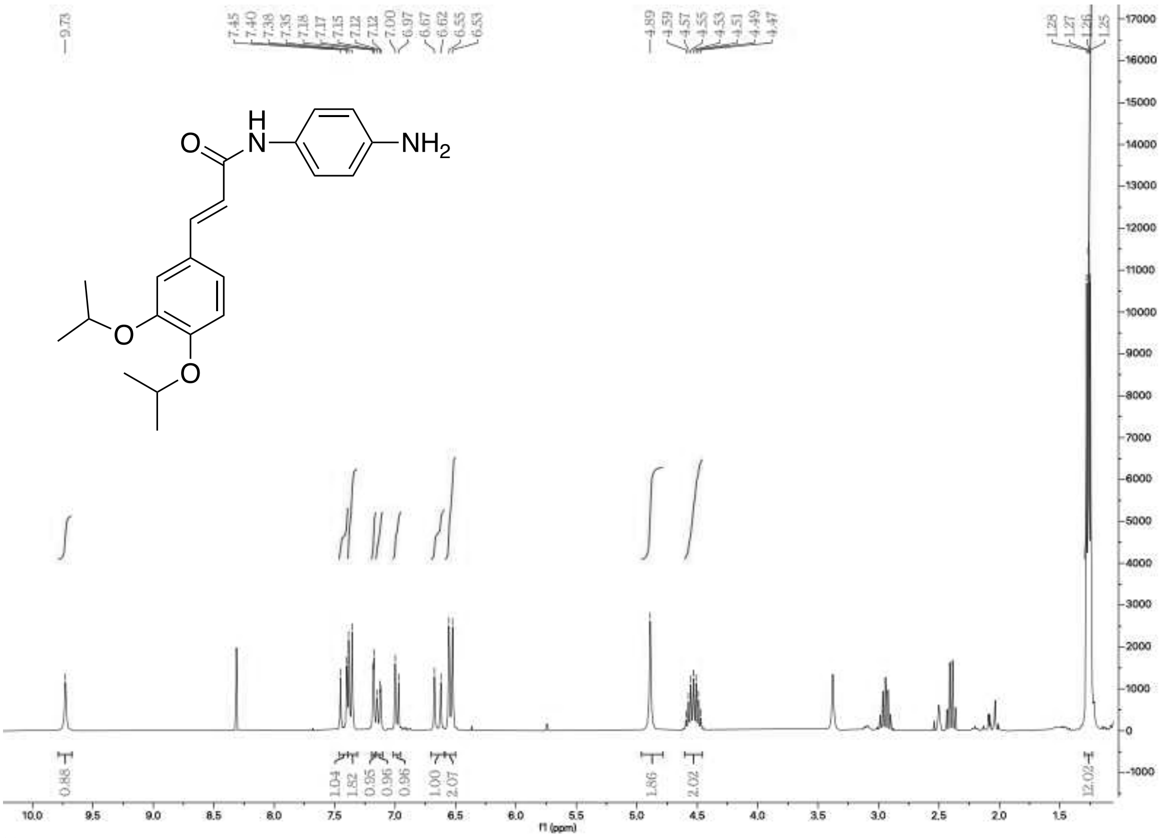

^{13}C NMR (100.6 MHz, DMSO-d_6)

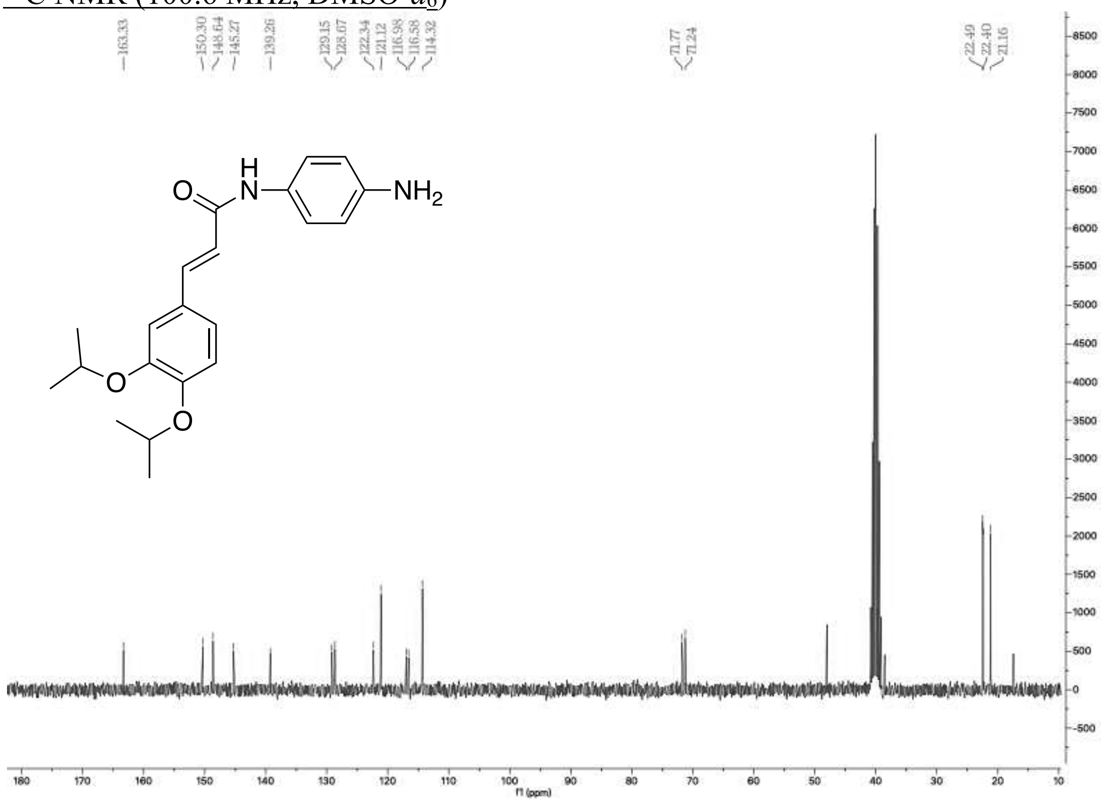

2.6. Spectra of **3f**

^{1}H NMR (400 MHz, DMSO-d_6)

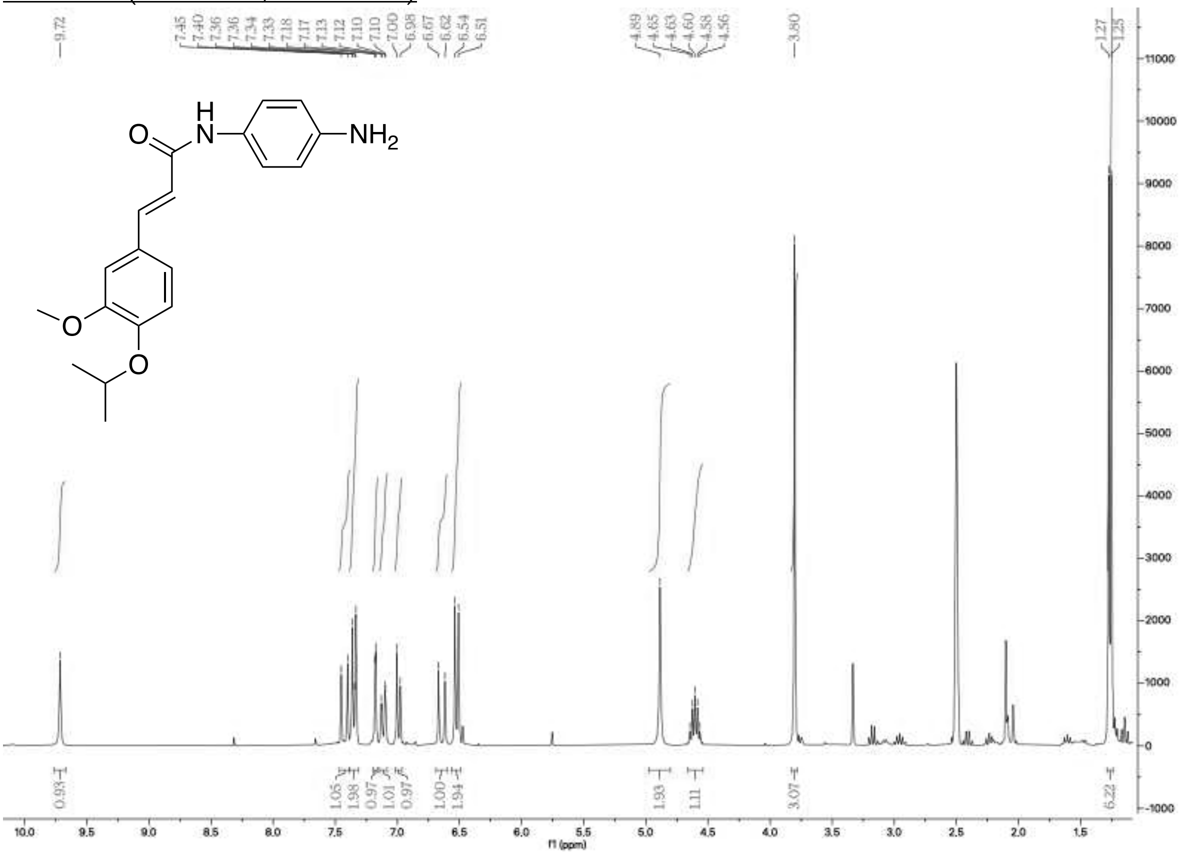

^{13}C NMR (100.6 MHz, DMSO-d_6)

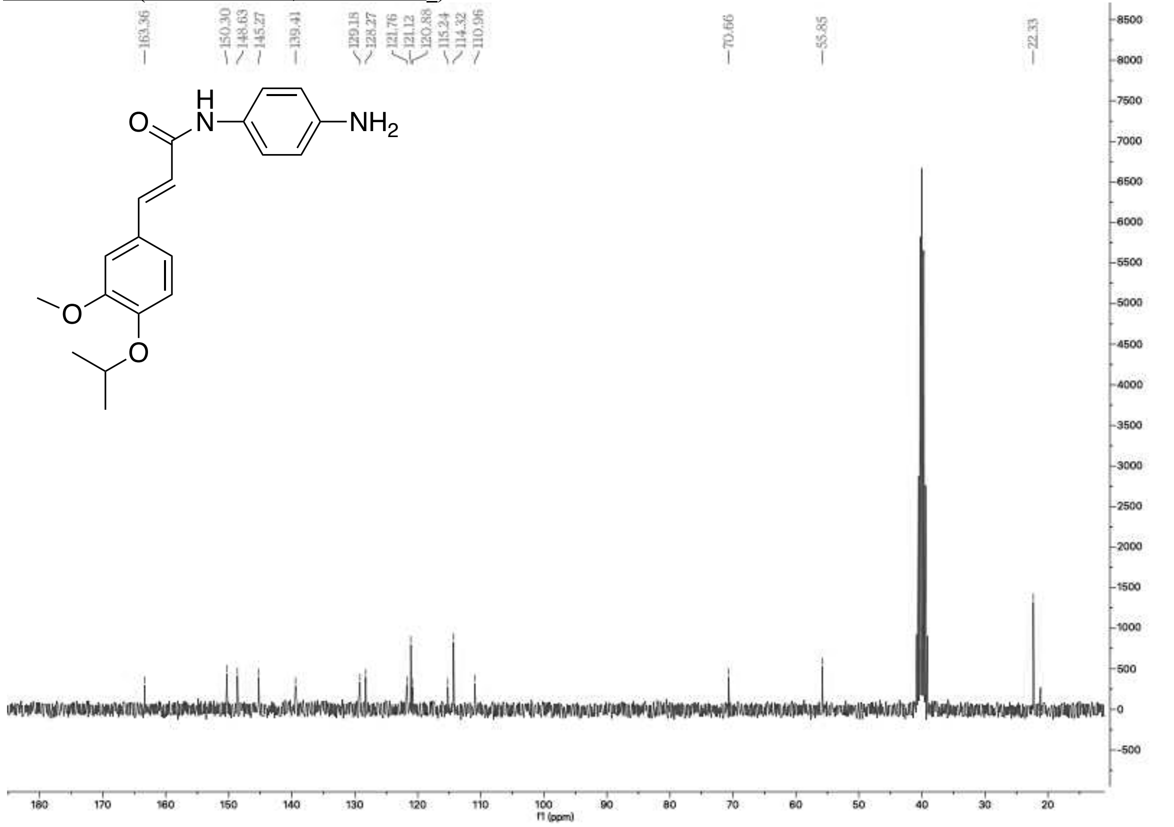

2.7. Spectra of **3g**

^{1}H NMR (400 MHz, DMSO-d_6)

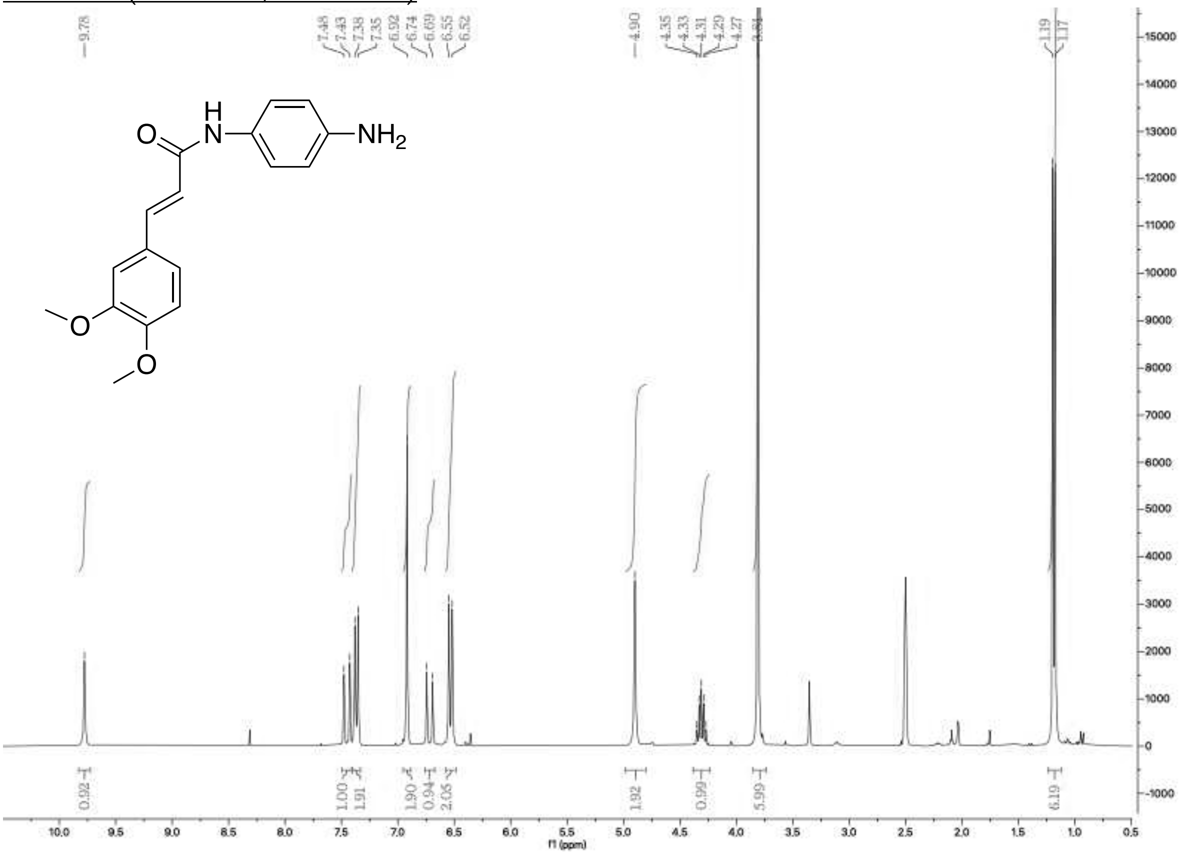

^{13}C NMR (100.6 MHz, DMSO-d_6)

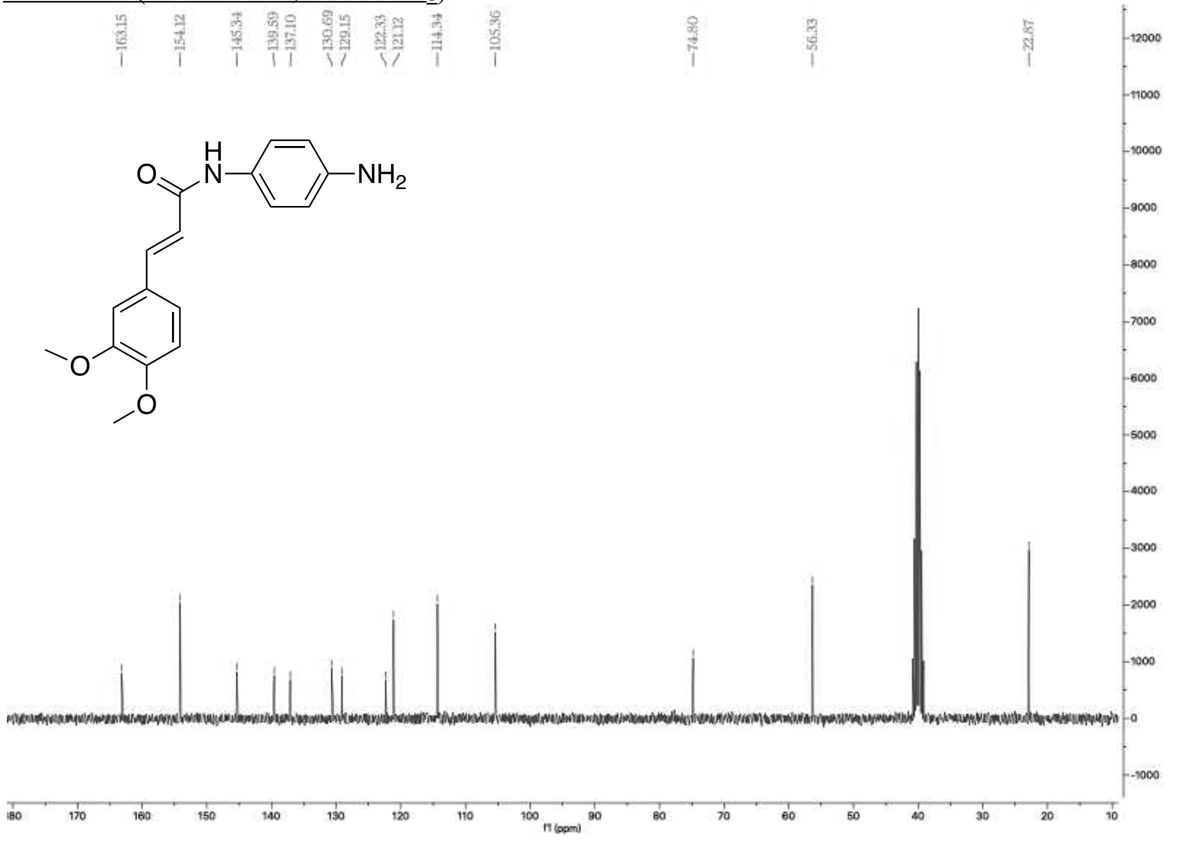

2.8. Spectra of **3h**

^{1}H NMR (300 MHz, DMSO-d_6)

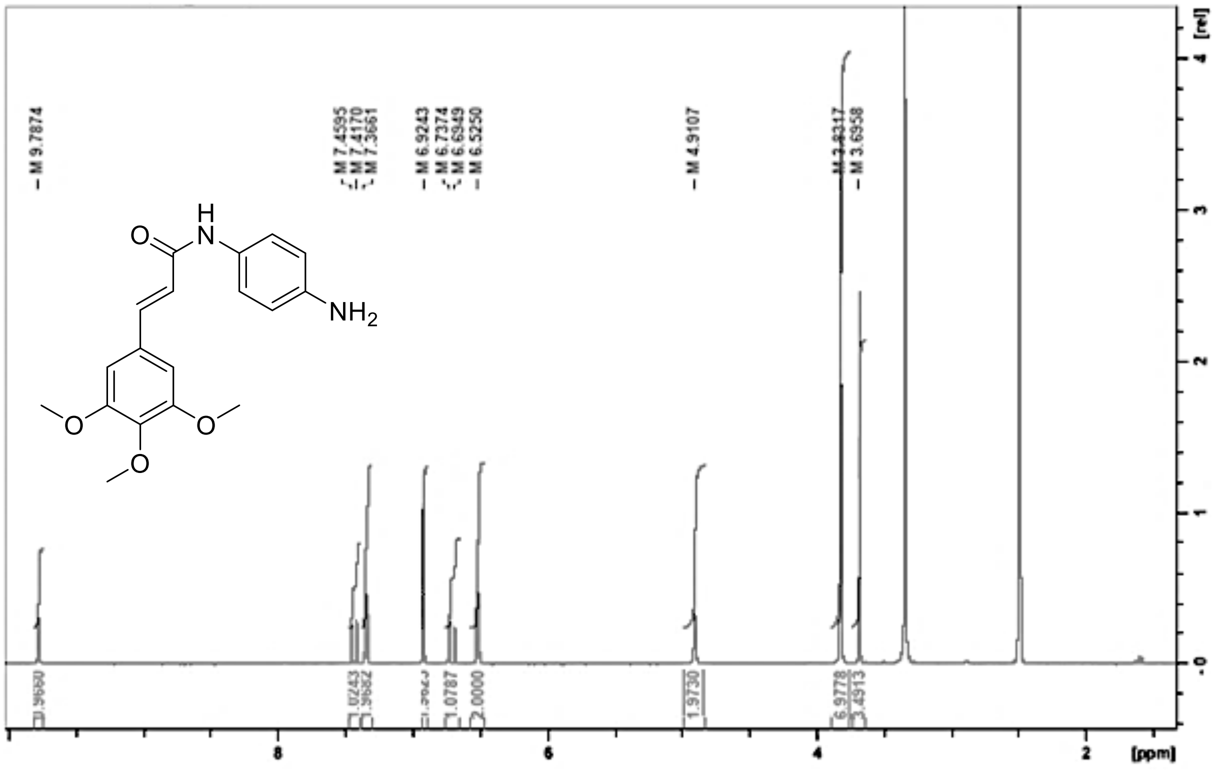

^{13}C NMR (75 MHz, DMSO-d_6)

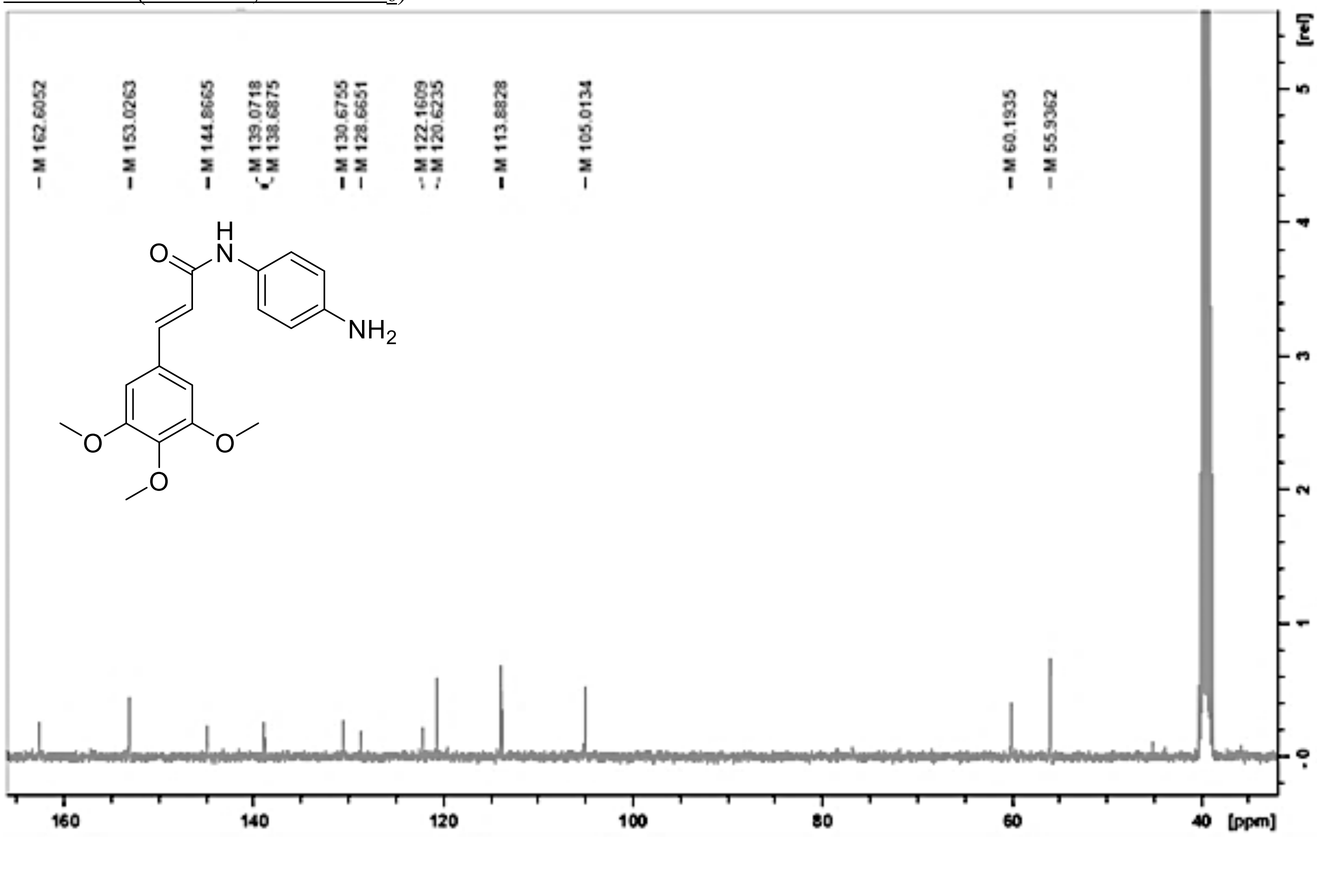

2.9. Spectra of **4a**

^{1}H NMR (400 MHz, DMSO-d_6)

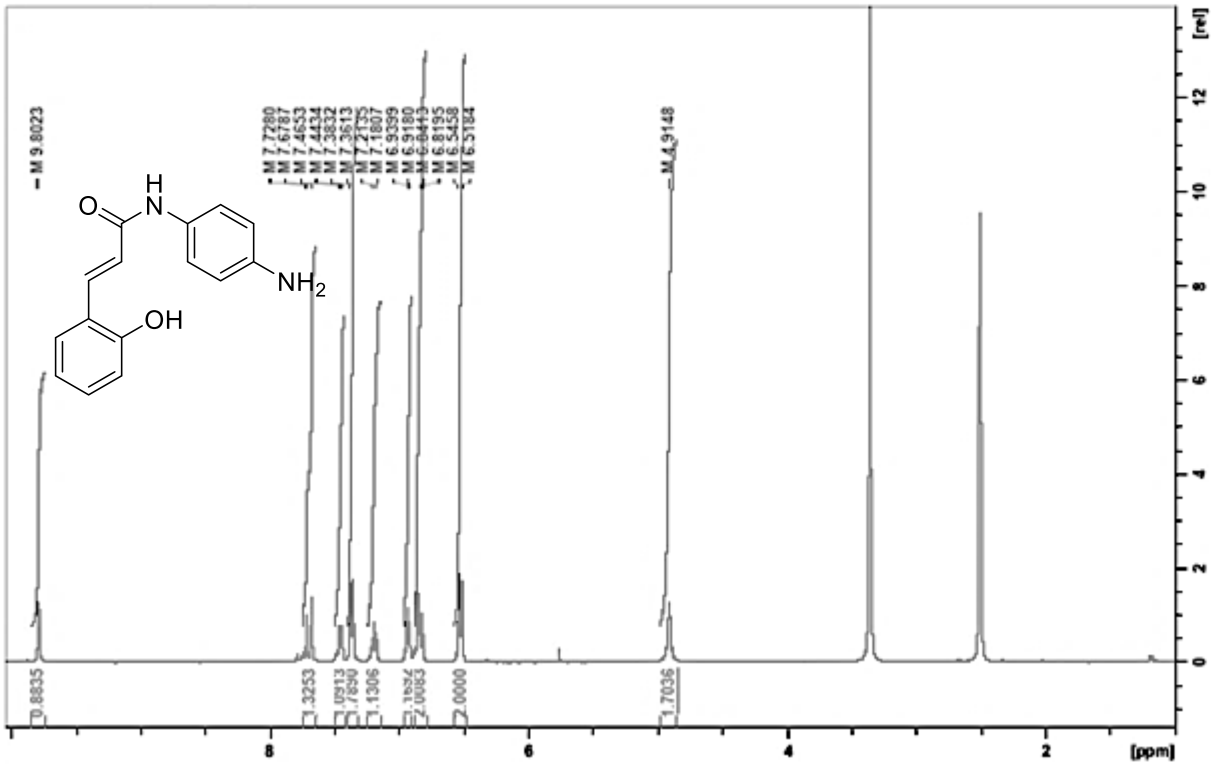

^{13}C NMR (100.6 MHz, DMSO-d_6)

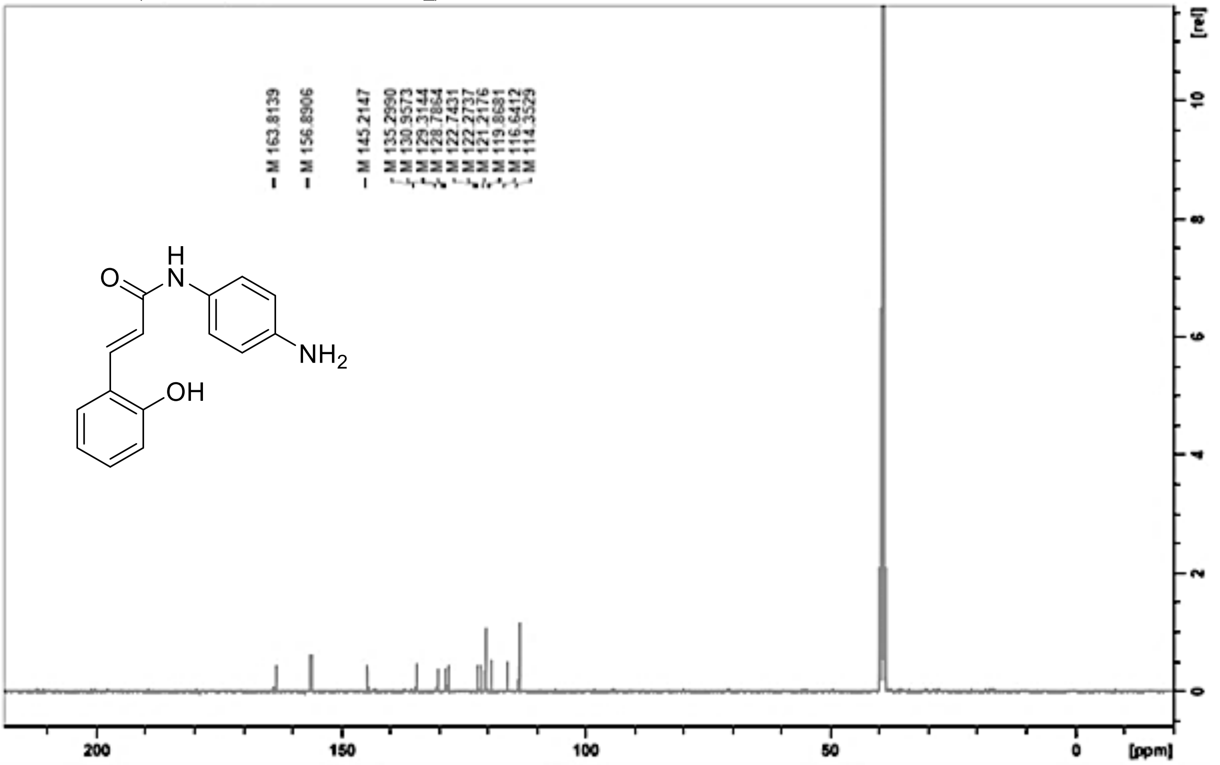

2.10. Spectra of **4b**

^{1}H NMR (300 MHz, DMSO-d_6)

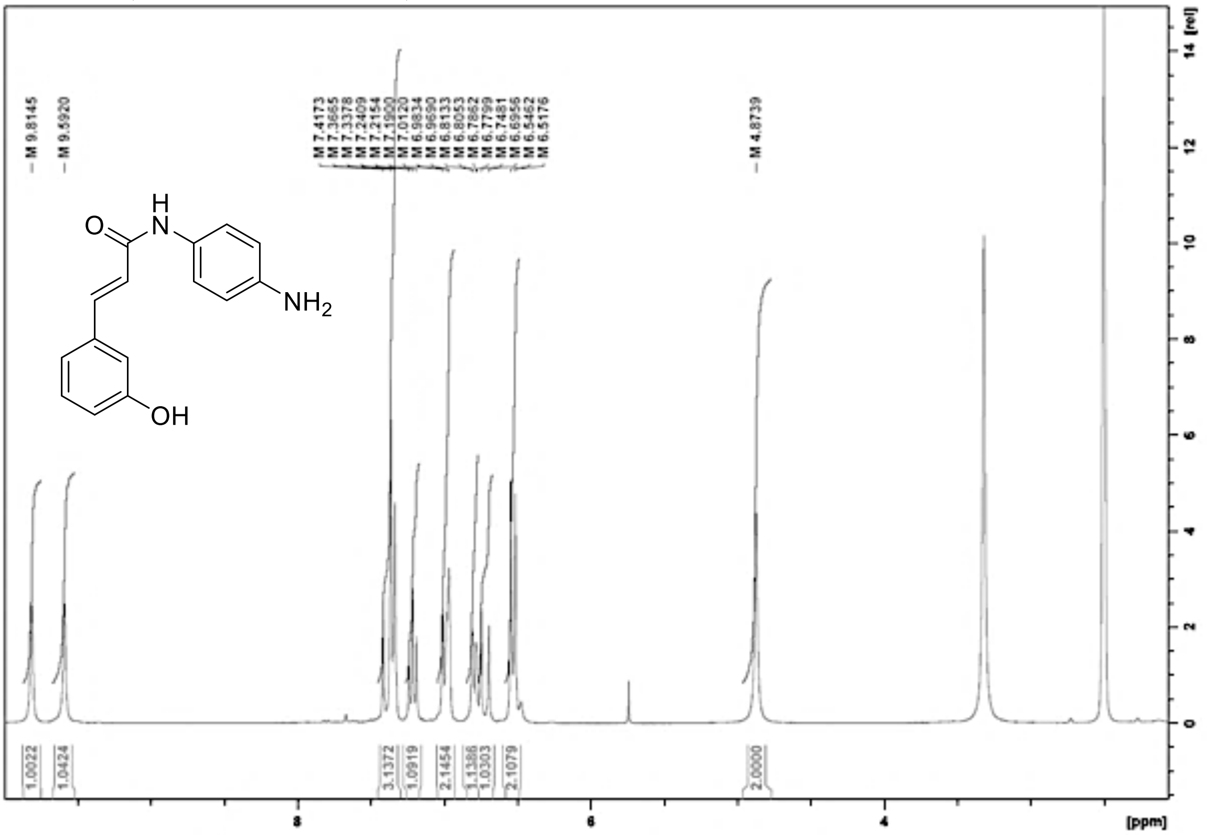

^{13}C NMR (75 MHz, DMSO-d_6)

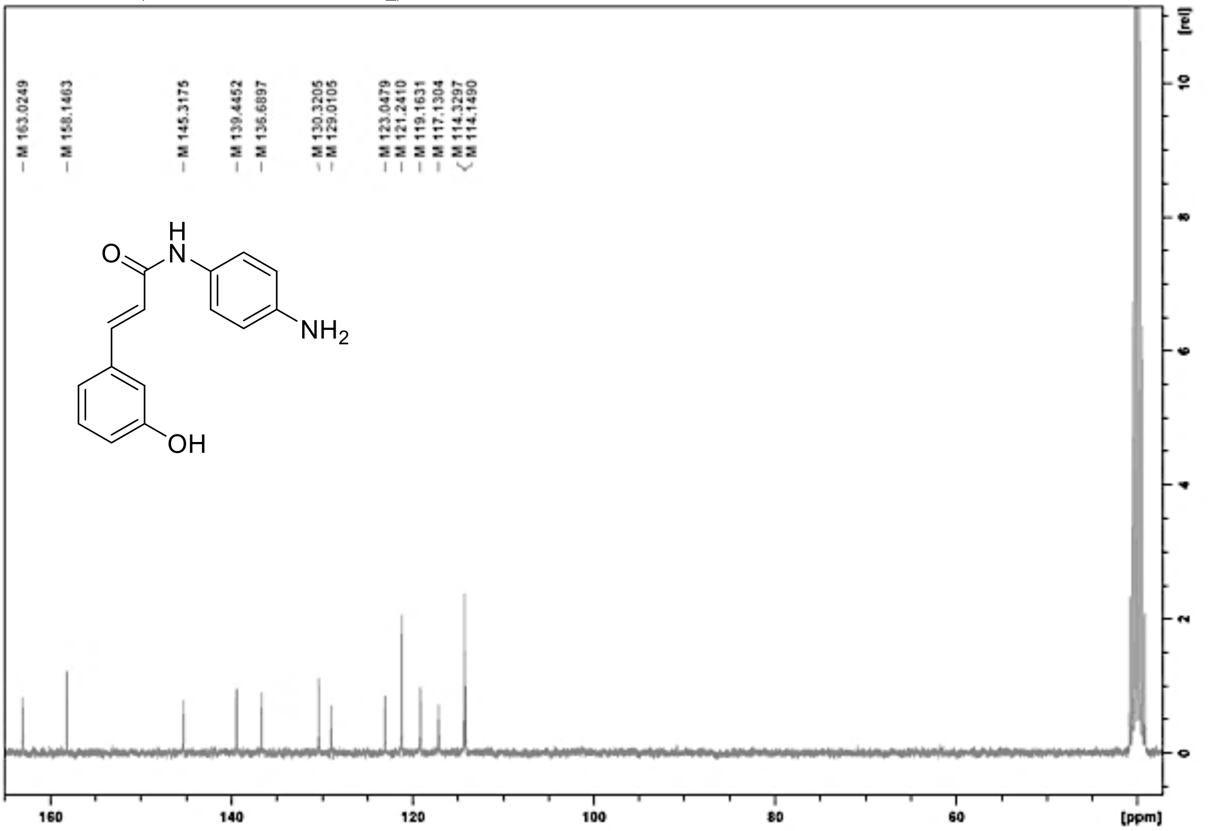

2.11. Spectra of **4c**

^{1}H NMR (300 MHz, DMSO-d_6)

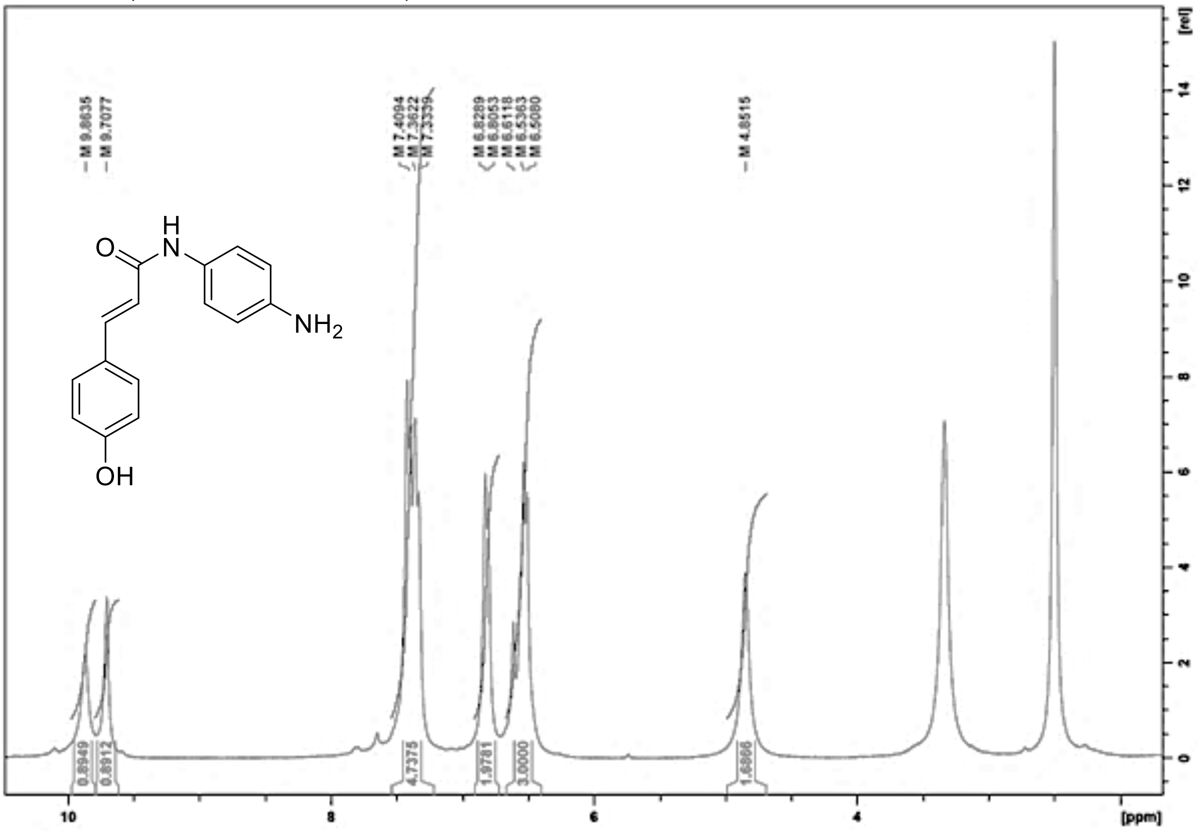

^{13}C NMR (75 MHz, DMSO-d_6)

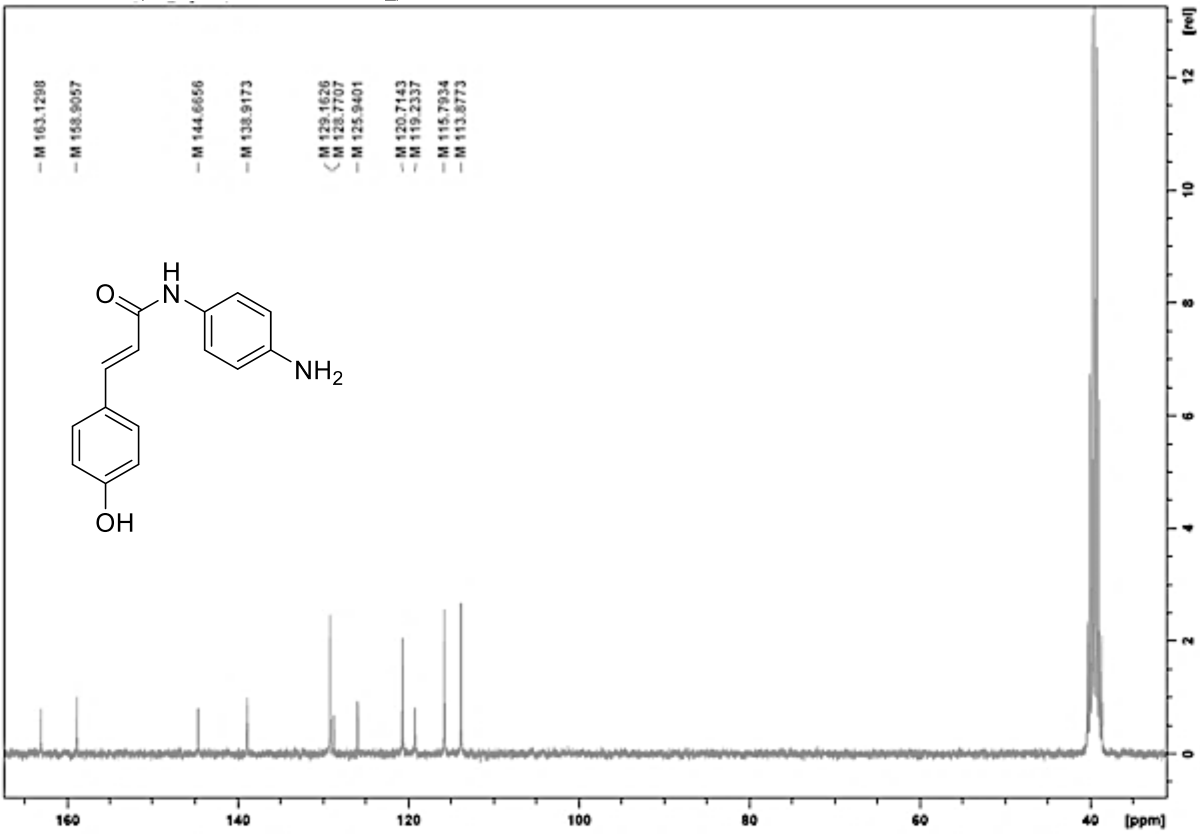

2.12. Spectra of **4d**

^{1}H NMR (400 MHz, DMSO-d_6)

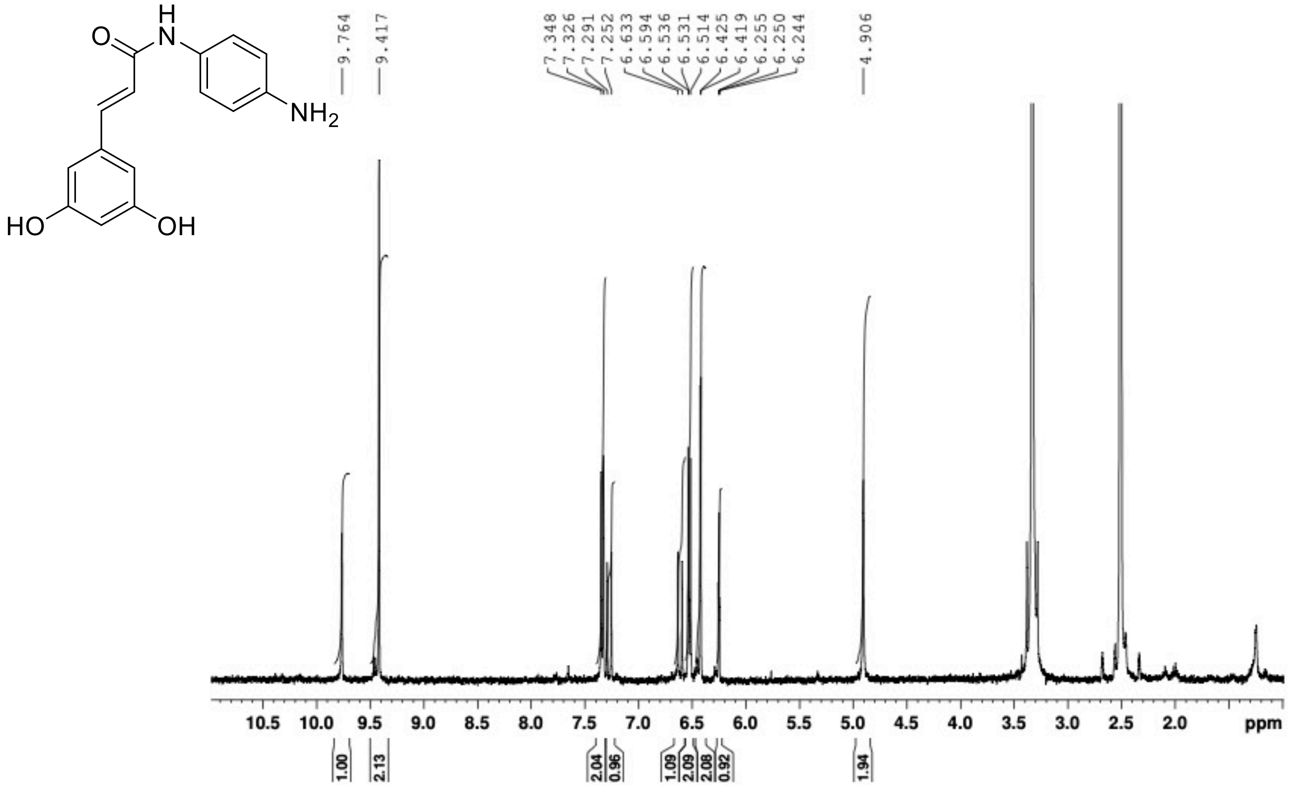

^{13}C NMR (100.6 MHz, DMSO-d_6)

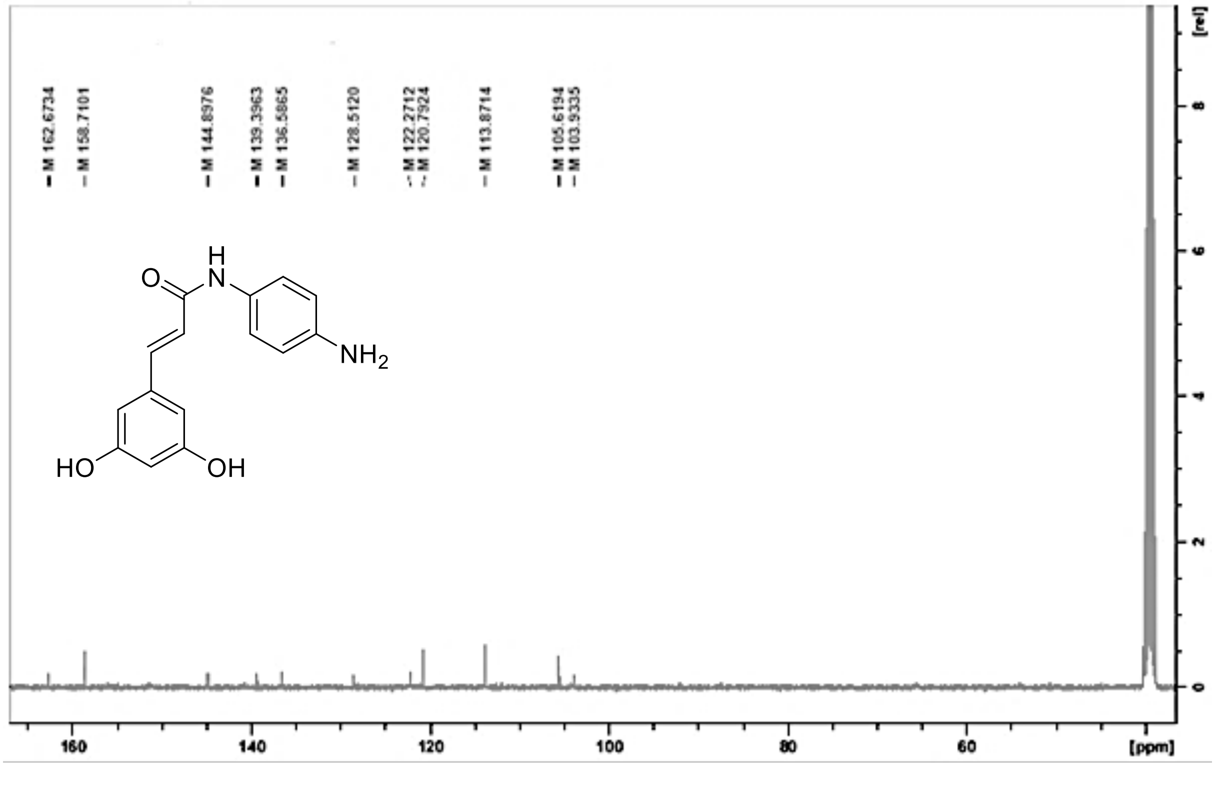

2.13. Spectra of **4e**

^{1}H NMR (300 MHz, DMSO-d_6)

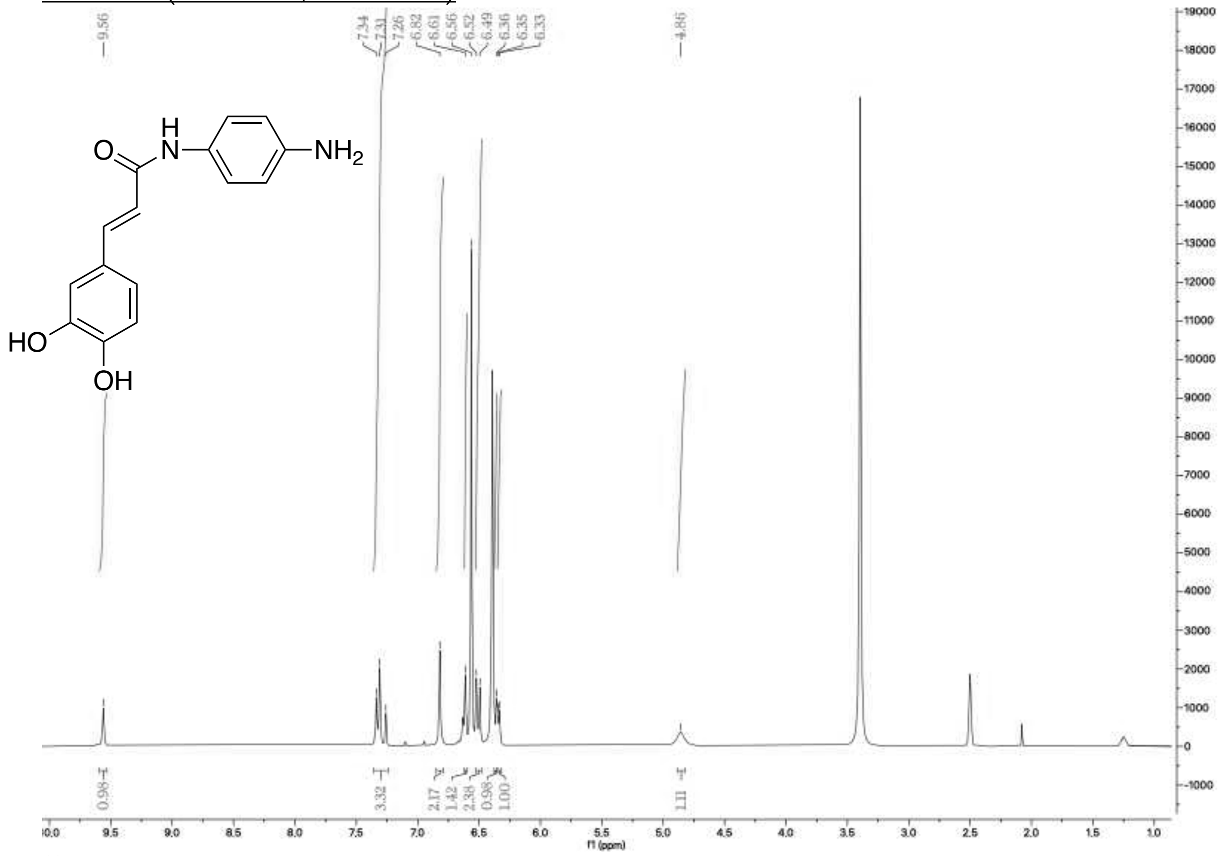

^{13}C NMR (100.6 MHz, DMSO-d_6)

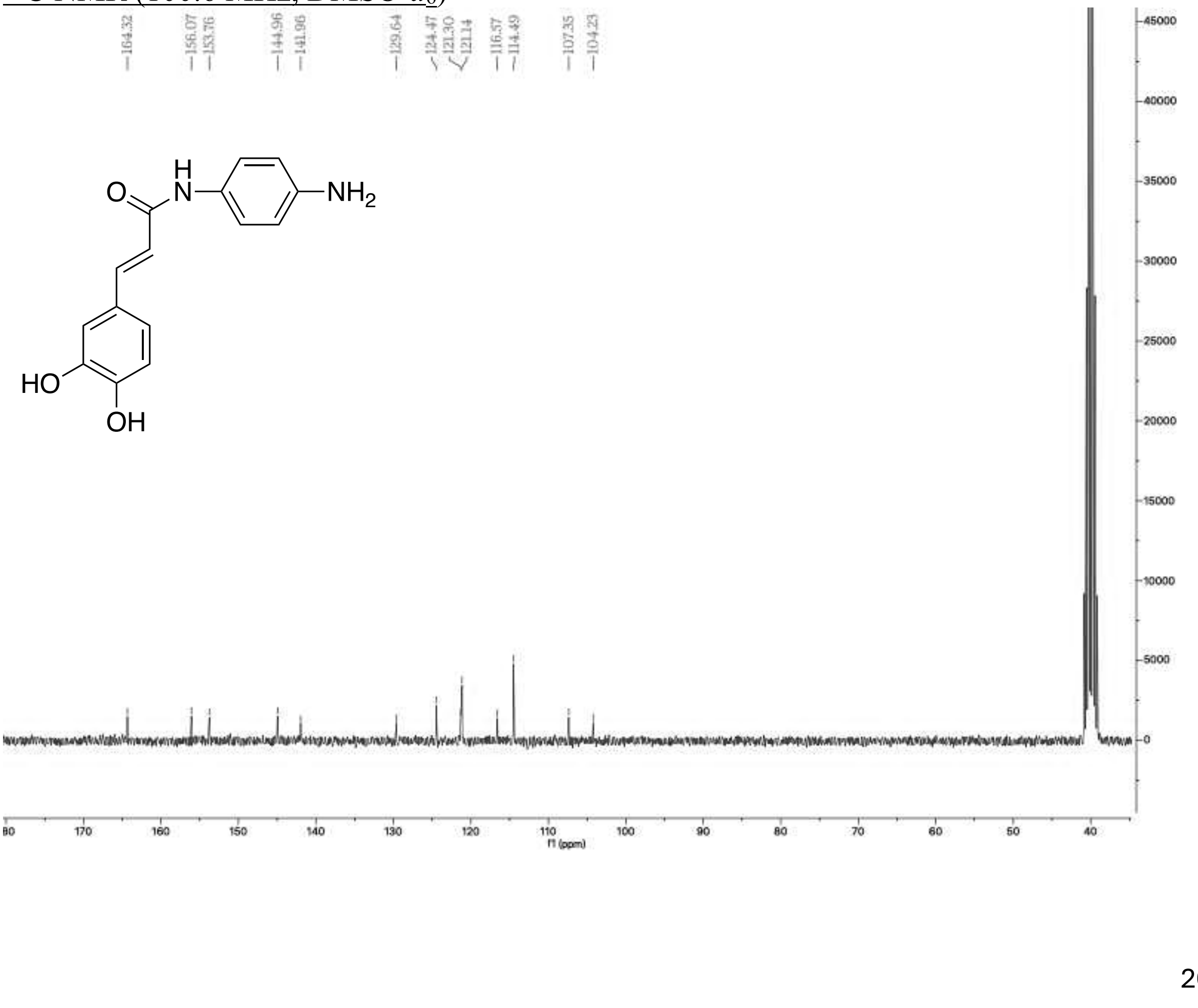

2.14. Spectra of **4f**

^{1}H NMR (300 MHz, DMSO-d_6)

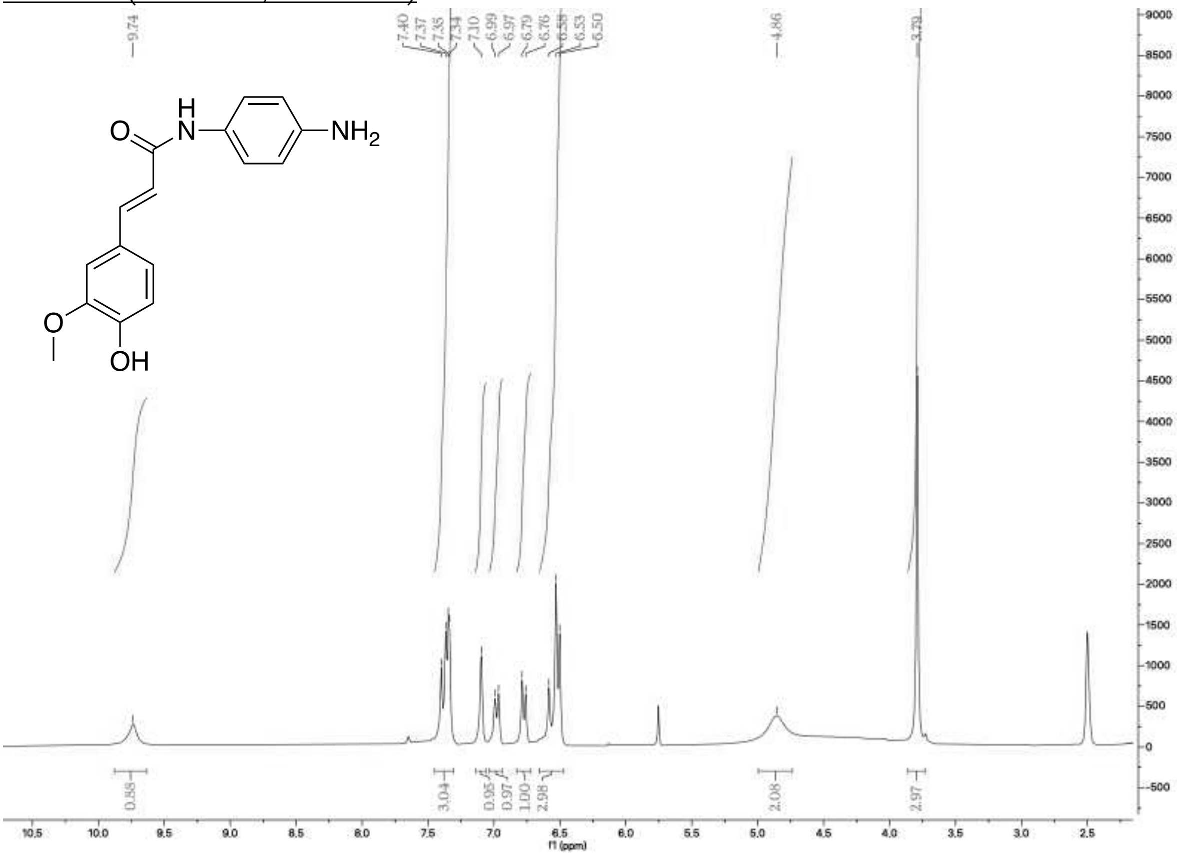

^{13}C NMR (100.6 MHz, DMSO-d_6)

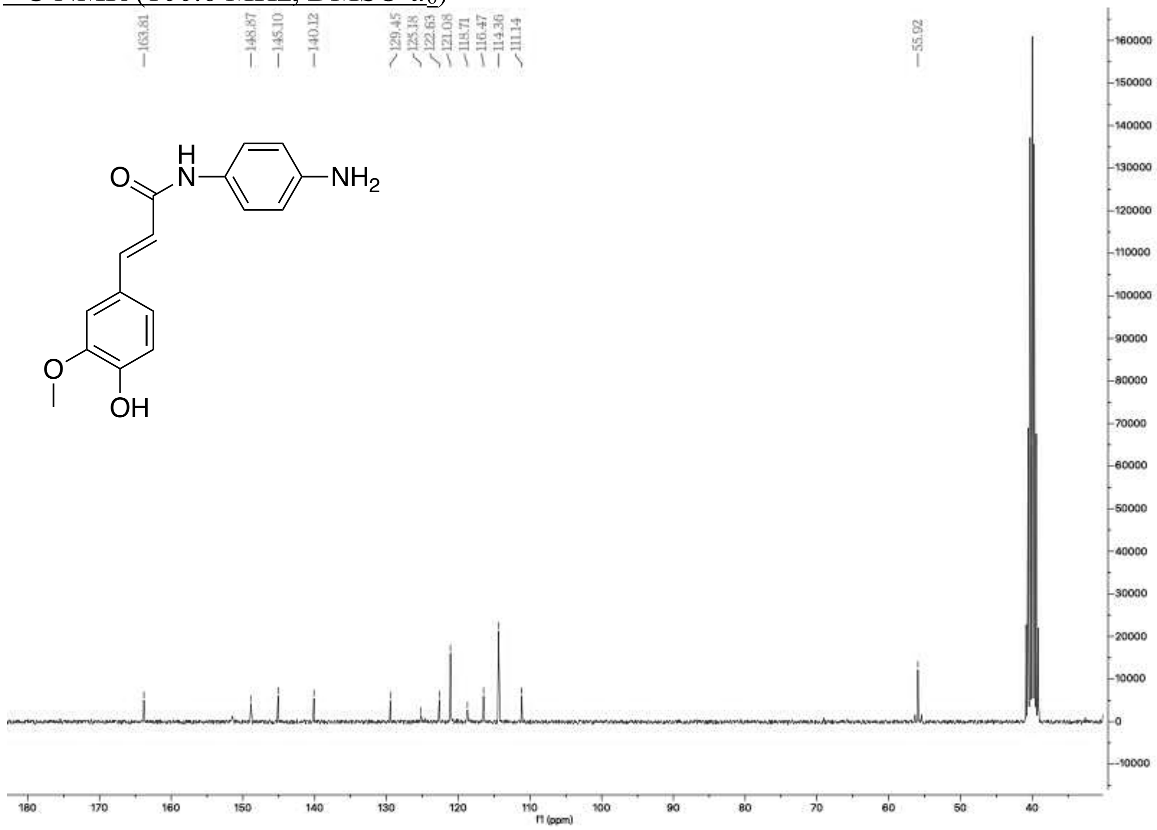

2.15. Spectra of **4g**

^{1}H NMR (300 MHz, DMSO-d_6)

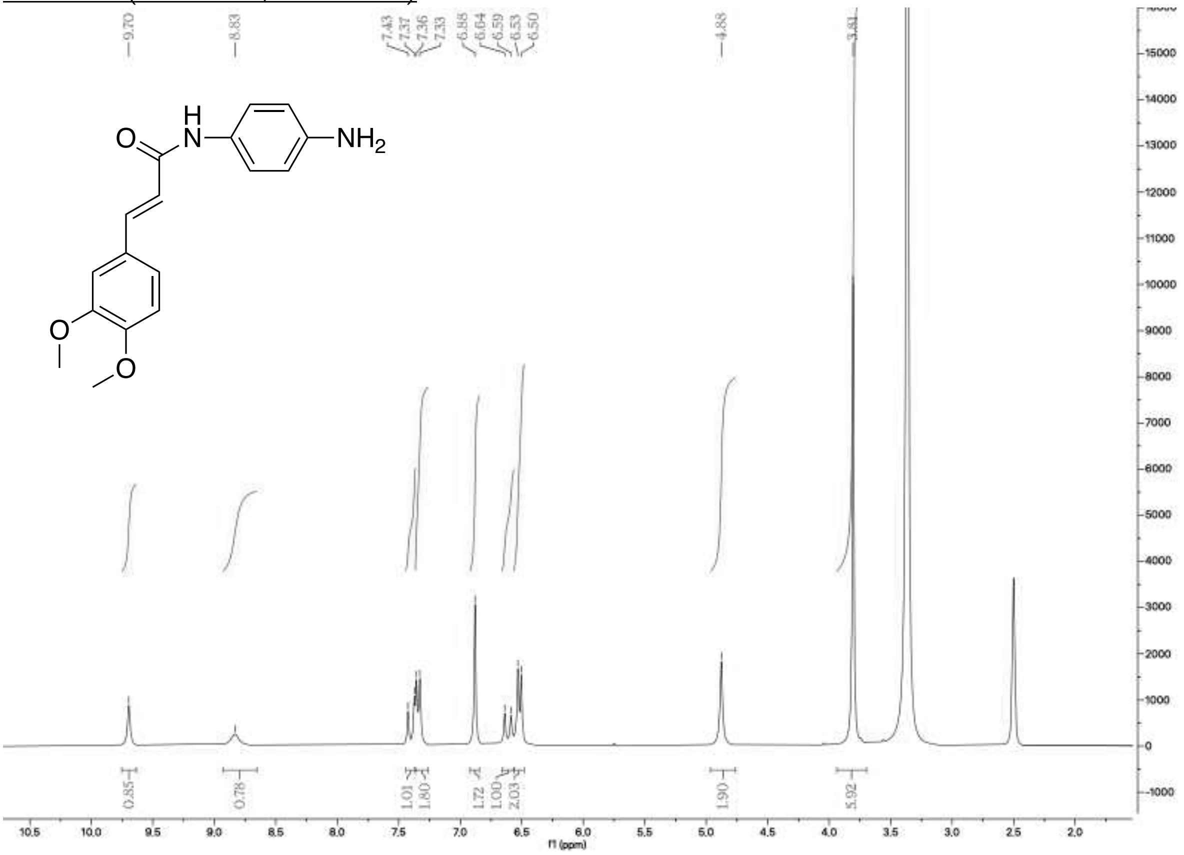

^{13}C NMR (100.6 MHz, DMSO-d_6)

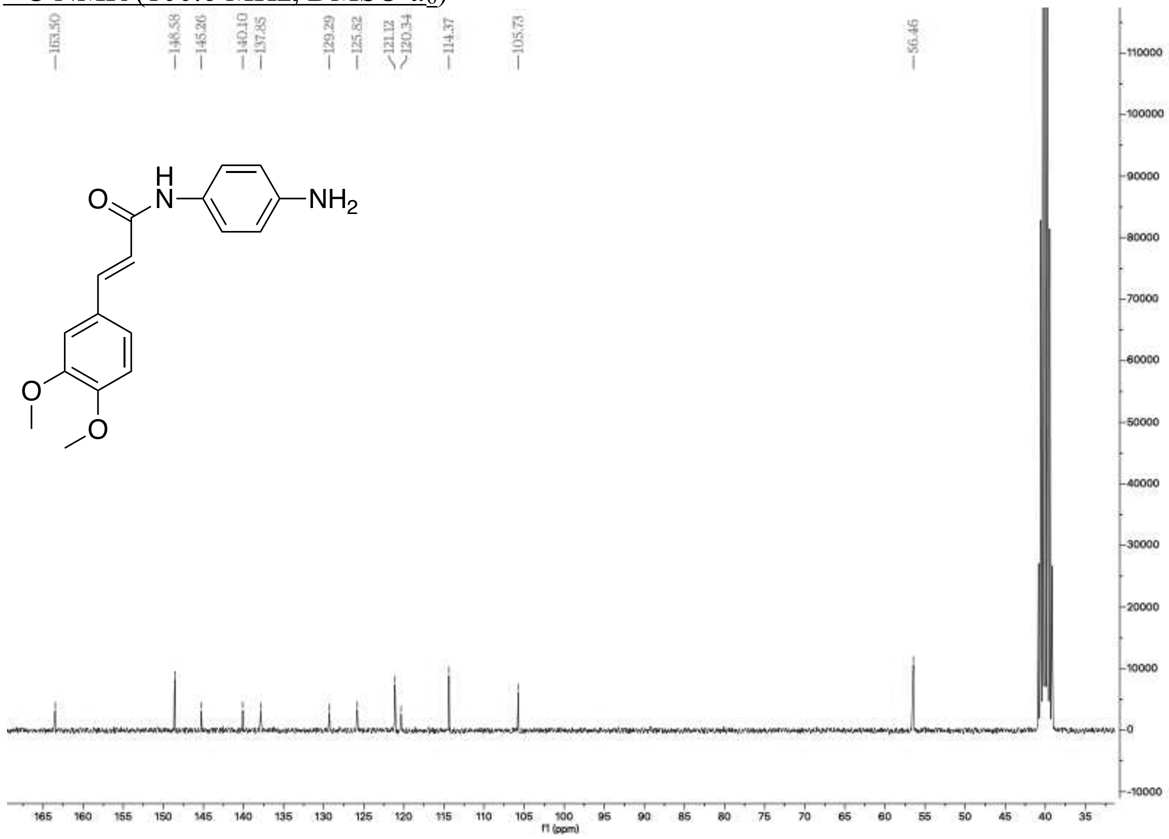

2.16. Spectra of **5a**

^{1}H NMR (400 MHz, DMSO-d_6)

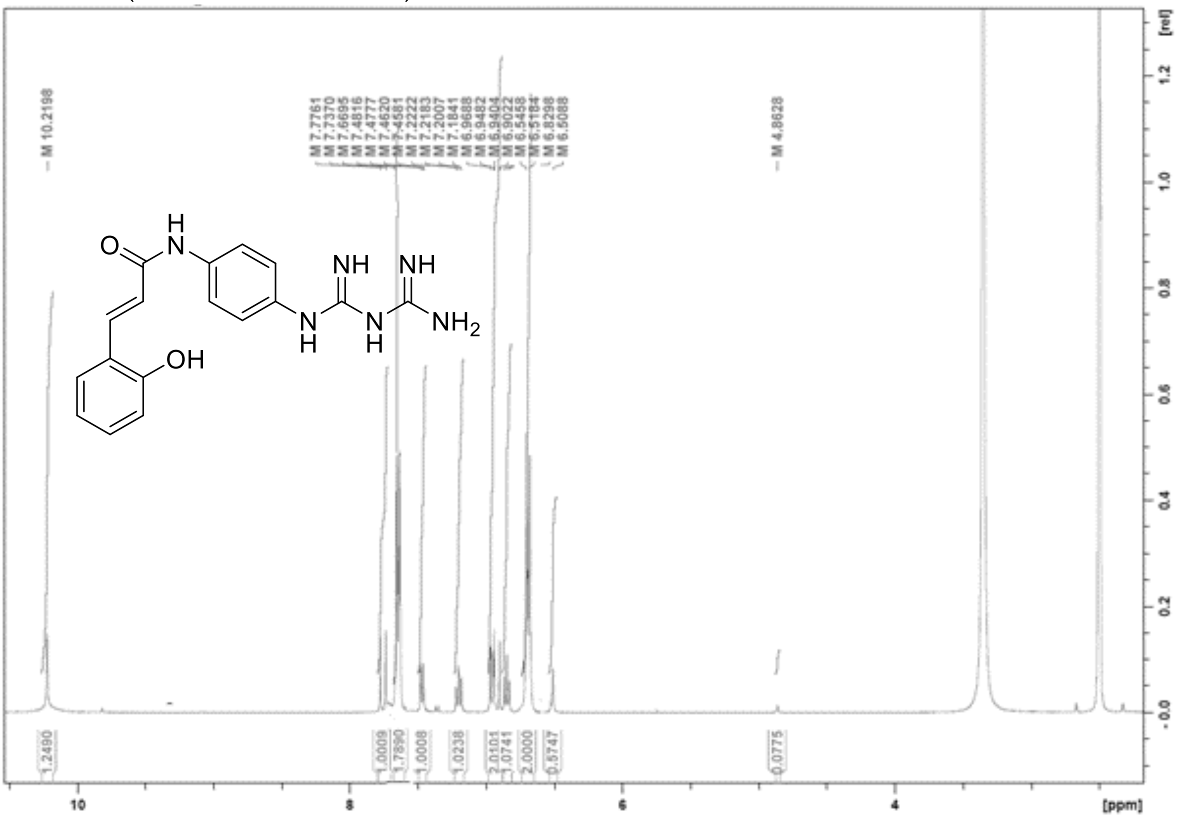

^{13}C NMR (100.6 MHz, DMSO-d_6)

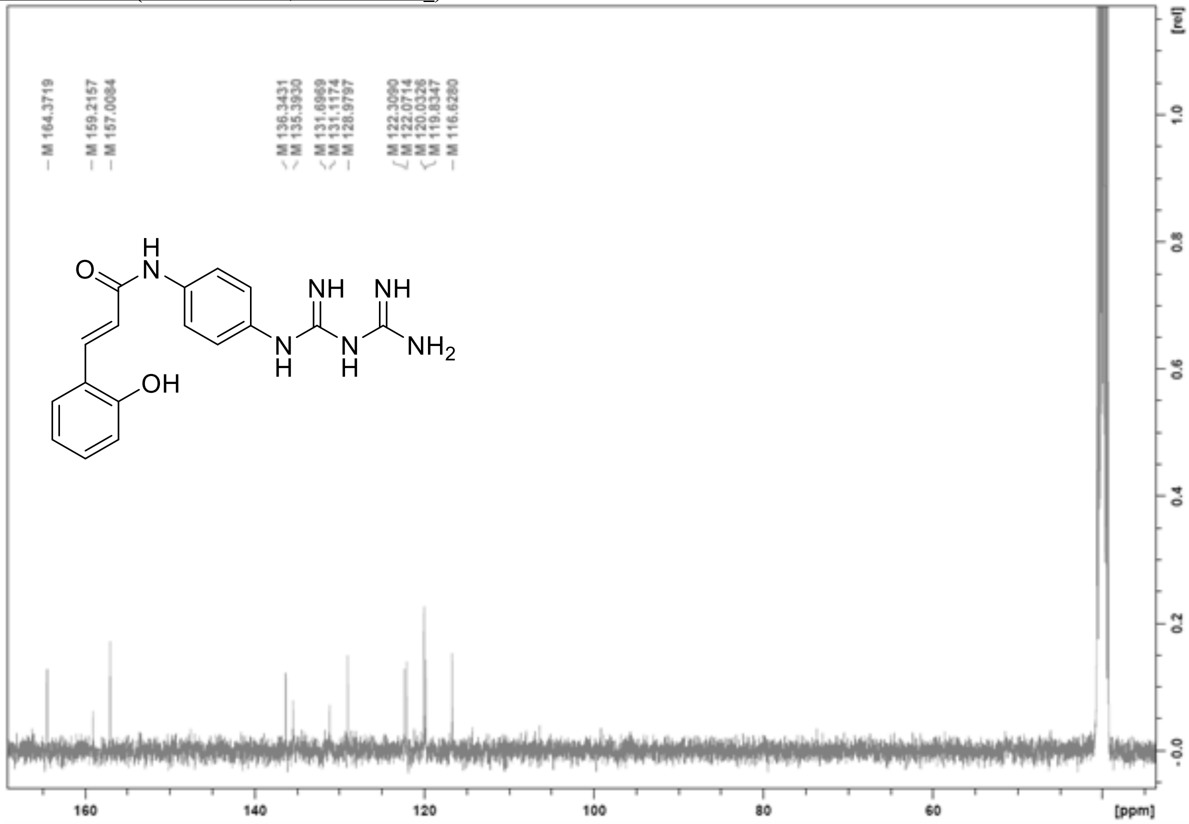

2.17. Spectra of **5b**

^{1}H NMR (400 MHz, DMSO-d_6)

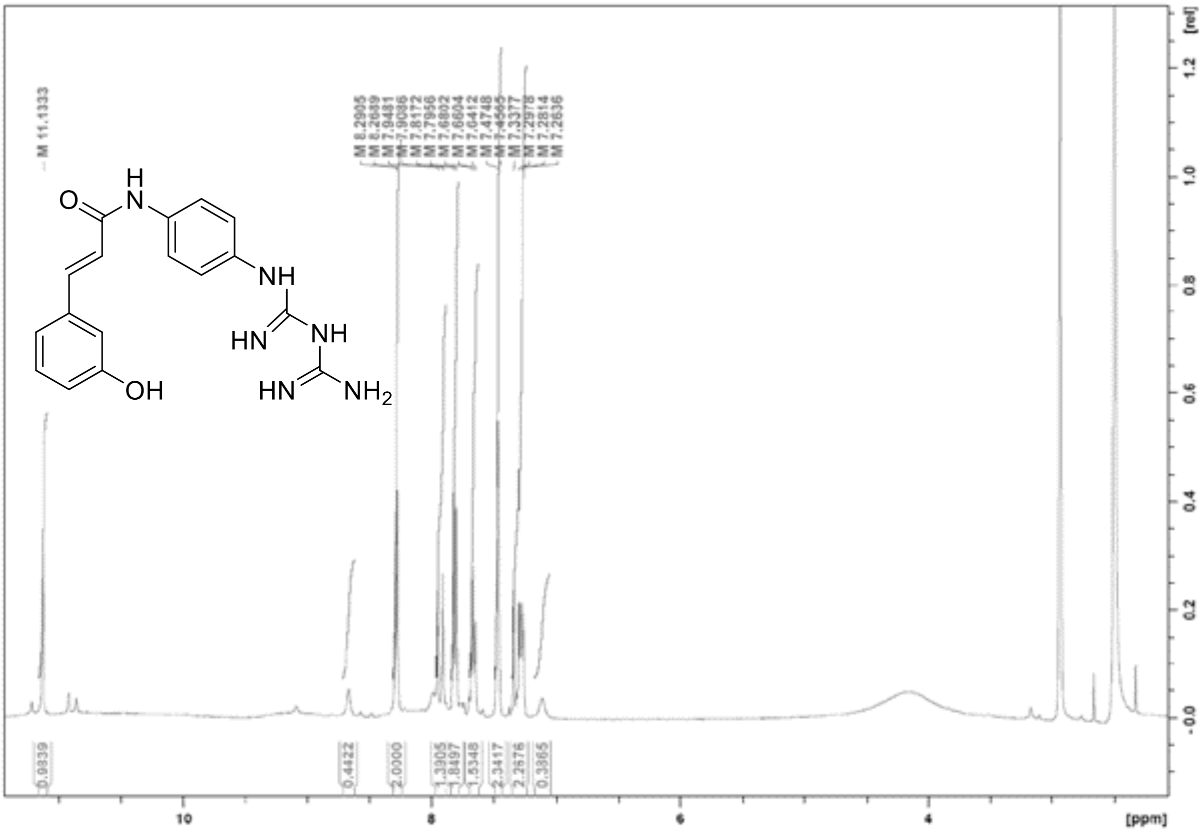

^{13}C NMR (100.6 MHz, DMSO-d_6)

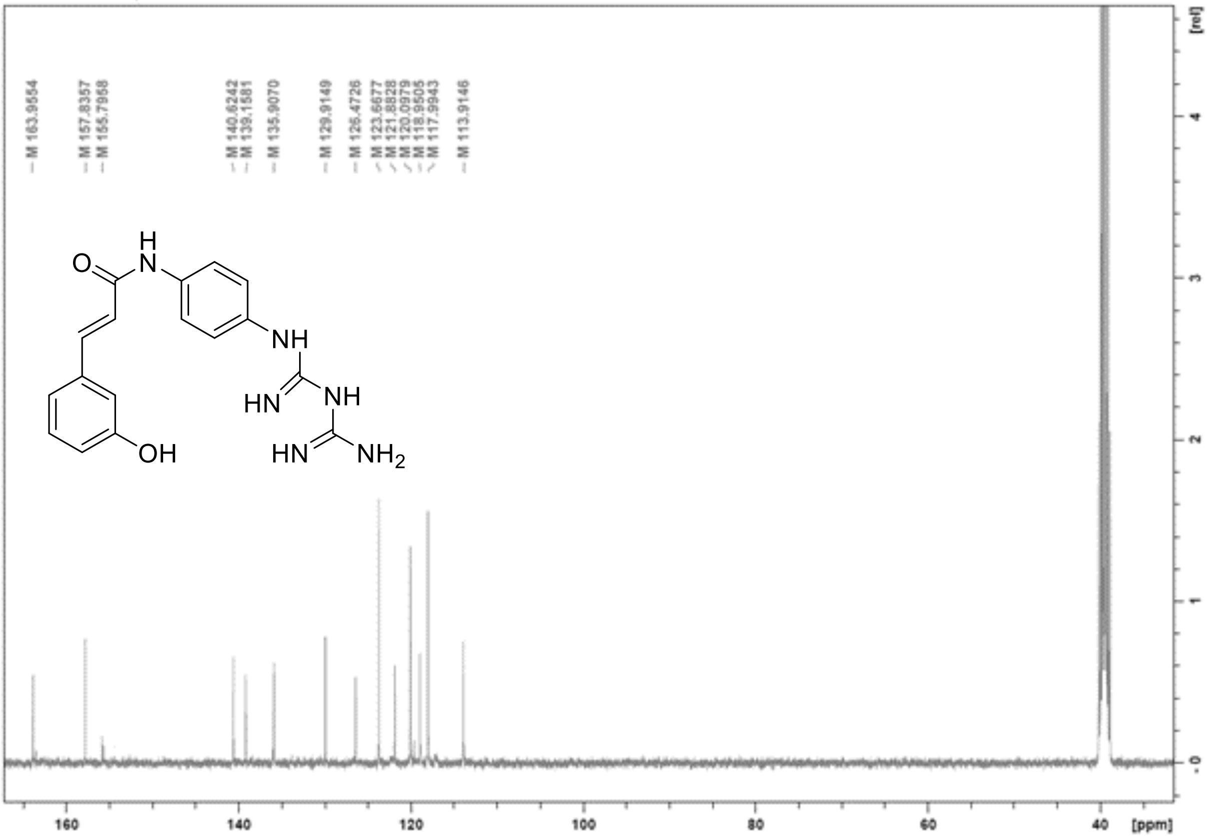

2.18. Spectra of **5c**

^{1}H NMR (400 MHz, DMSO-d_6)

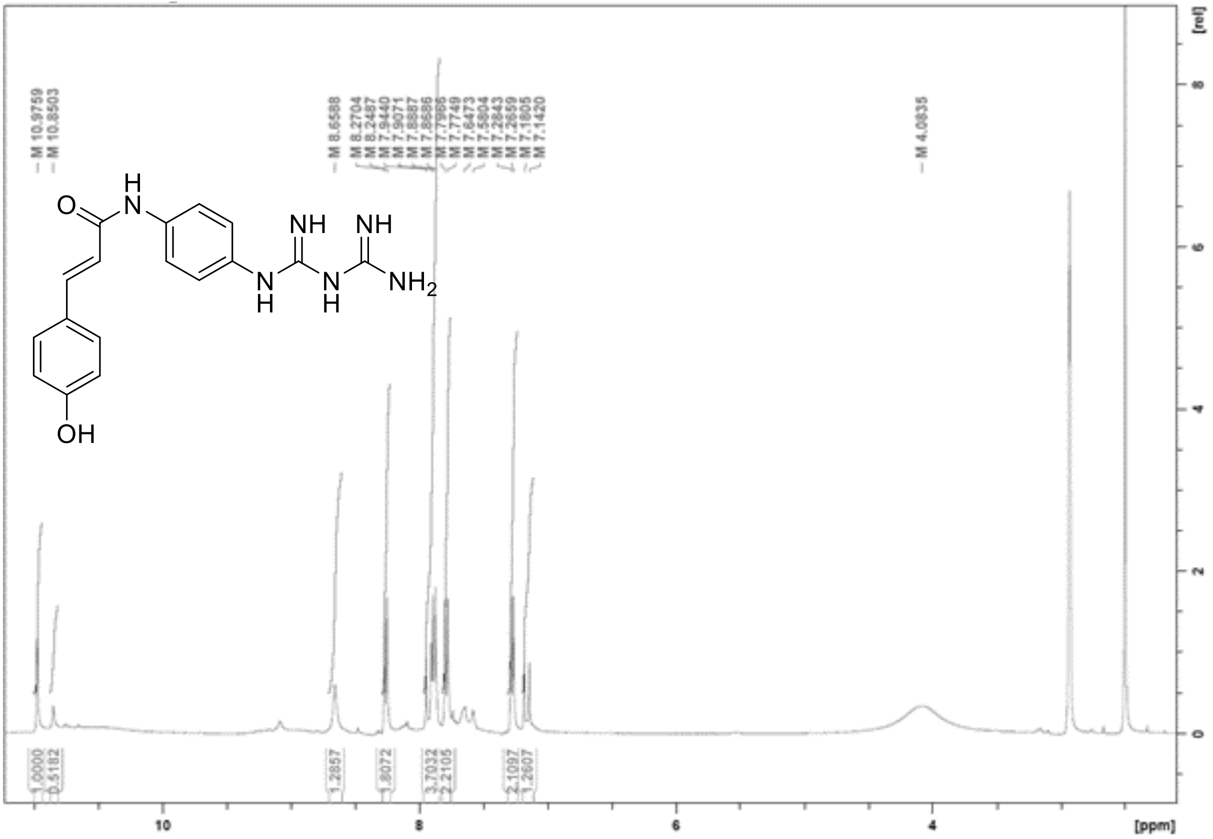

^{13}C NMR (100.6 MHz, DMSO-d_6)

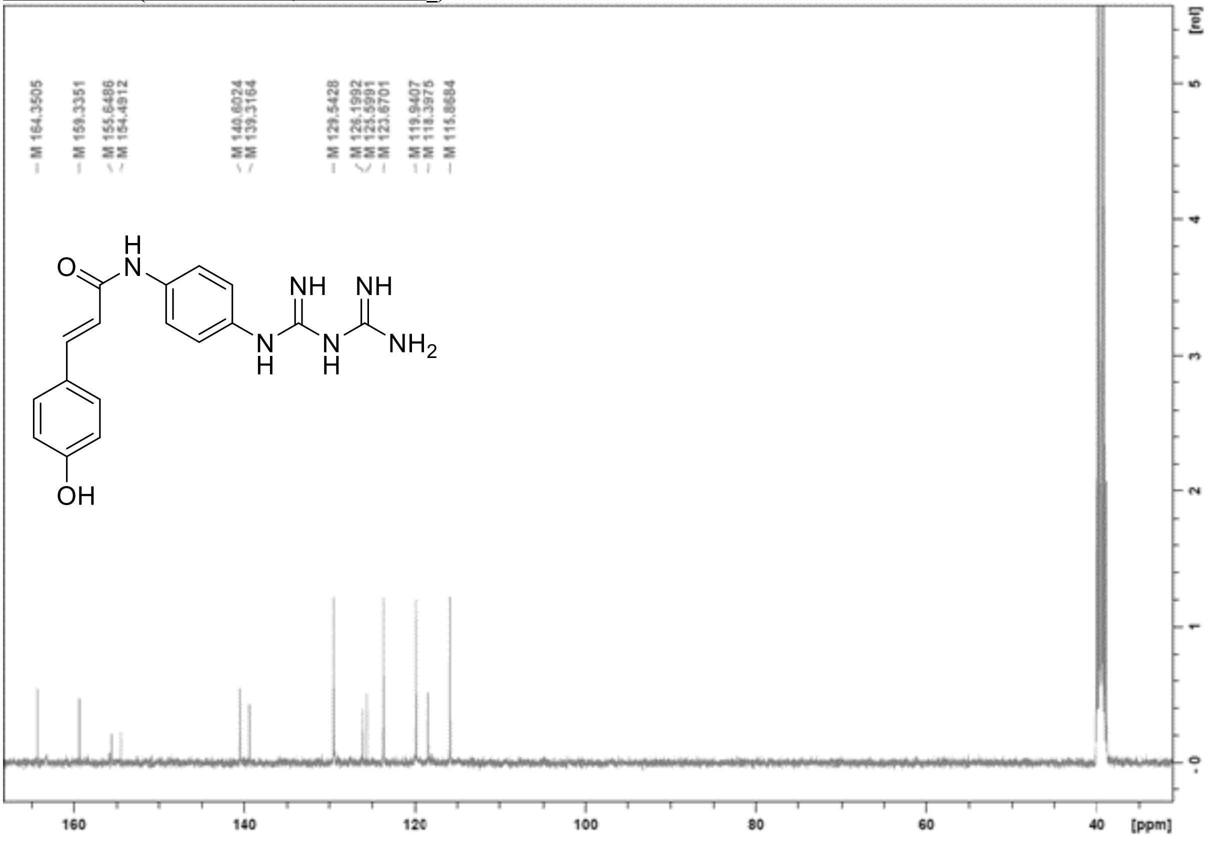

2.19. Spectra of **5d**

^{1}H NMR (400 MHz, DMSO-d_6)

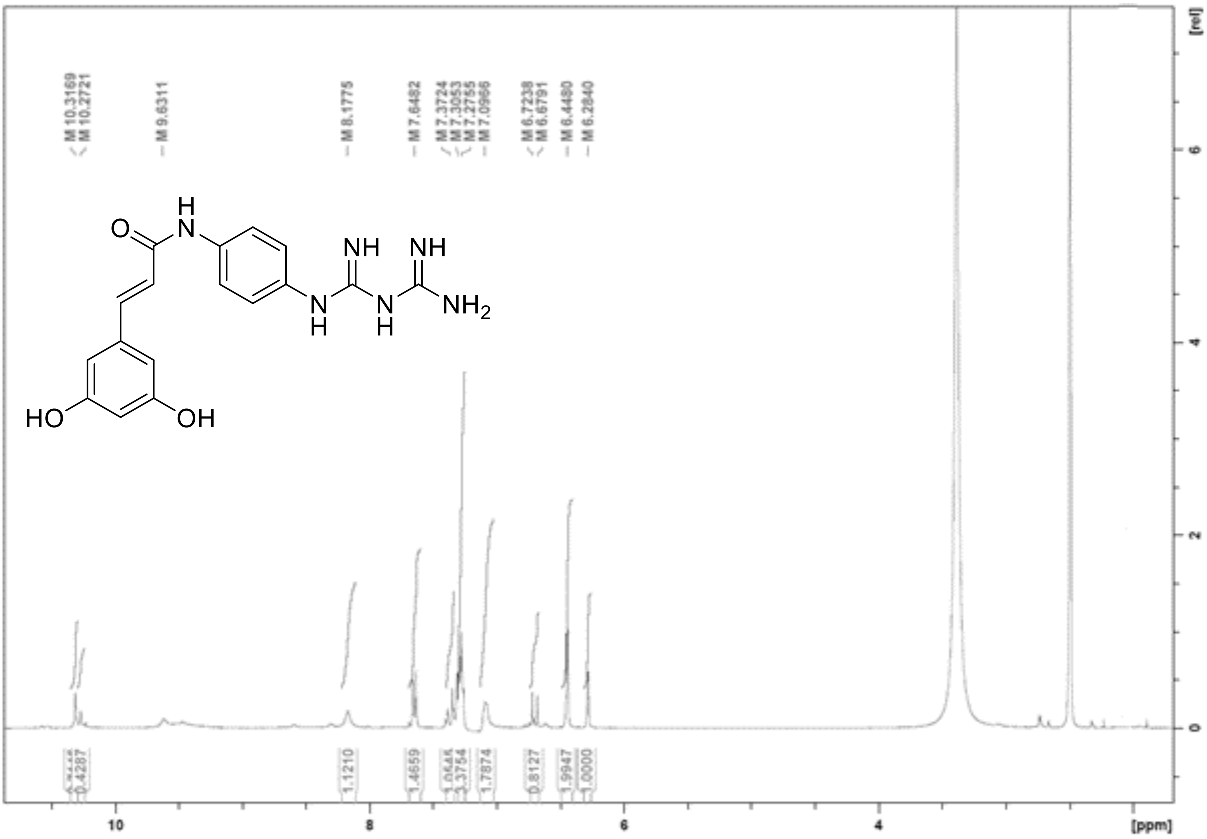

^{13}C NMR (100.6 MHz, DMSO-d_6)

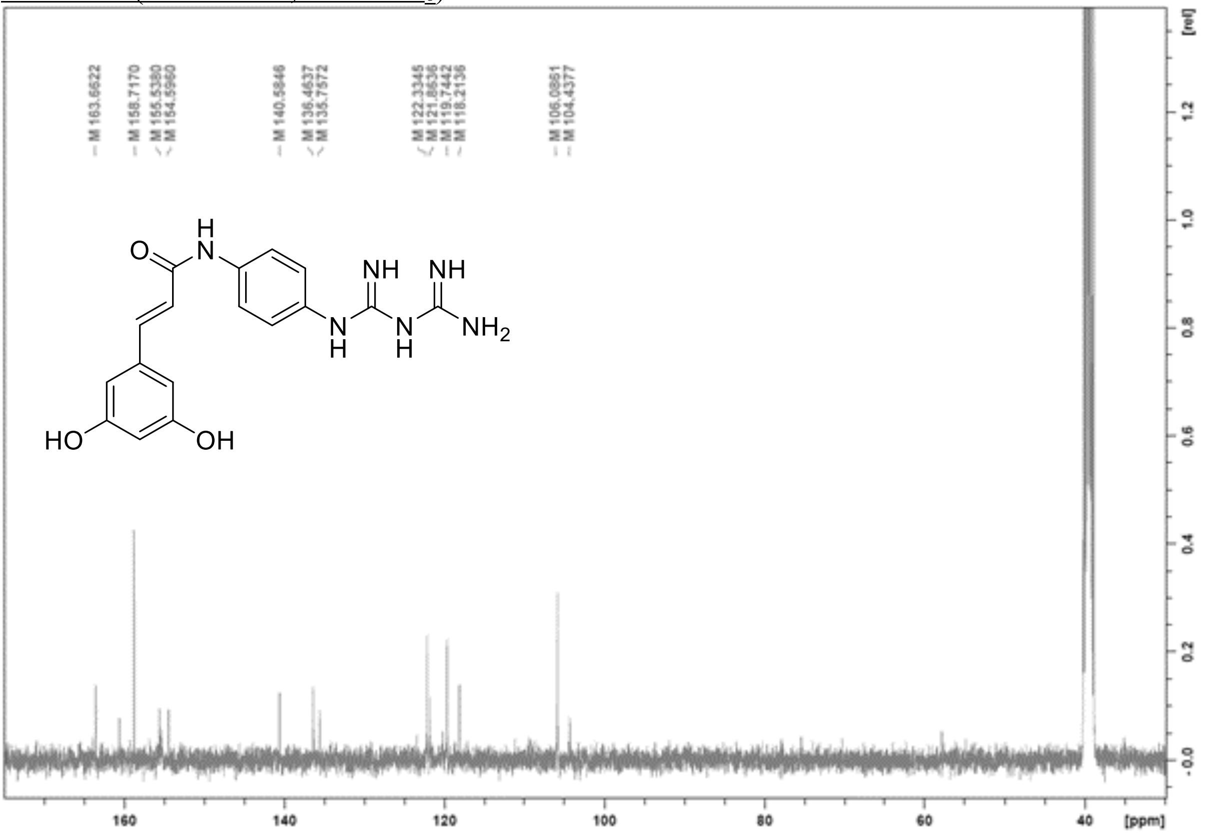

2.20. Spectra of **5e**

^{1}H NMR (400 MHz, DMSO-d_6)

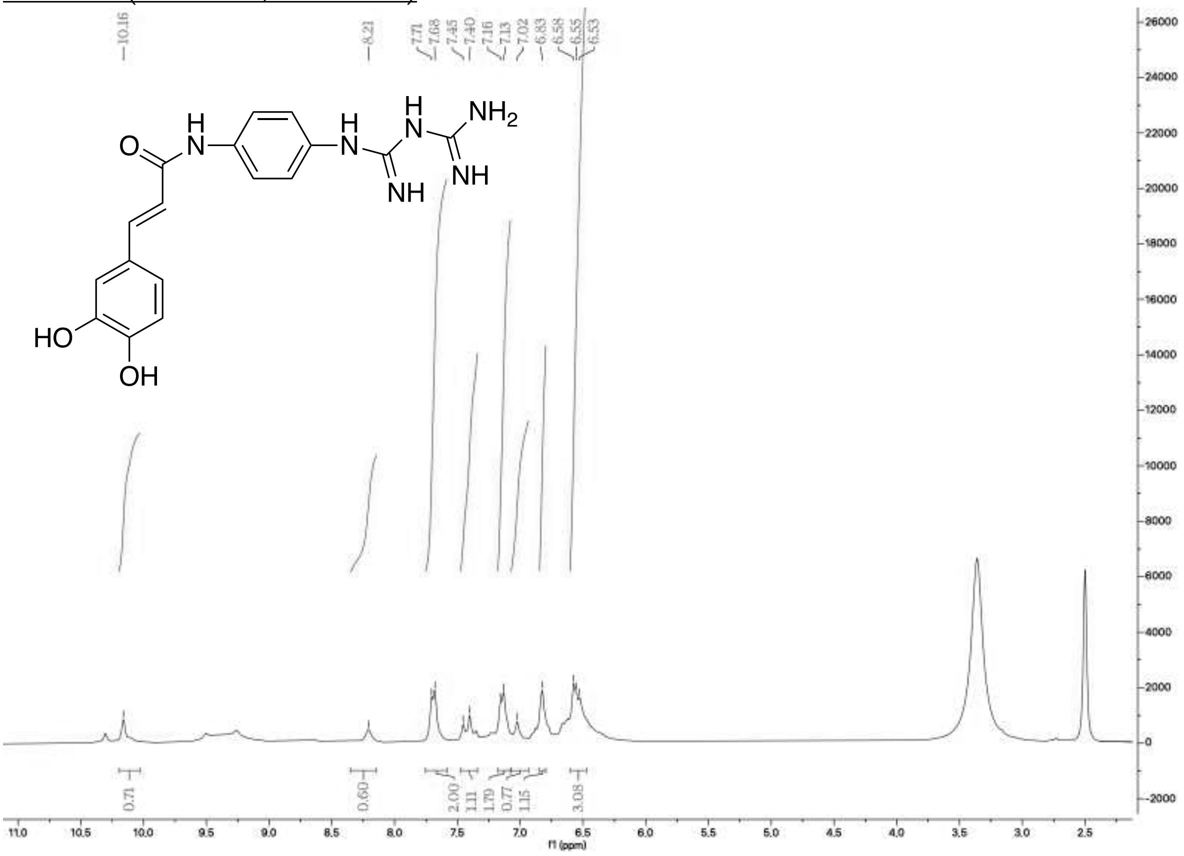

^{13}C NMR (100.6 MHz, DMSO-d_6)

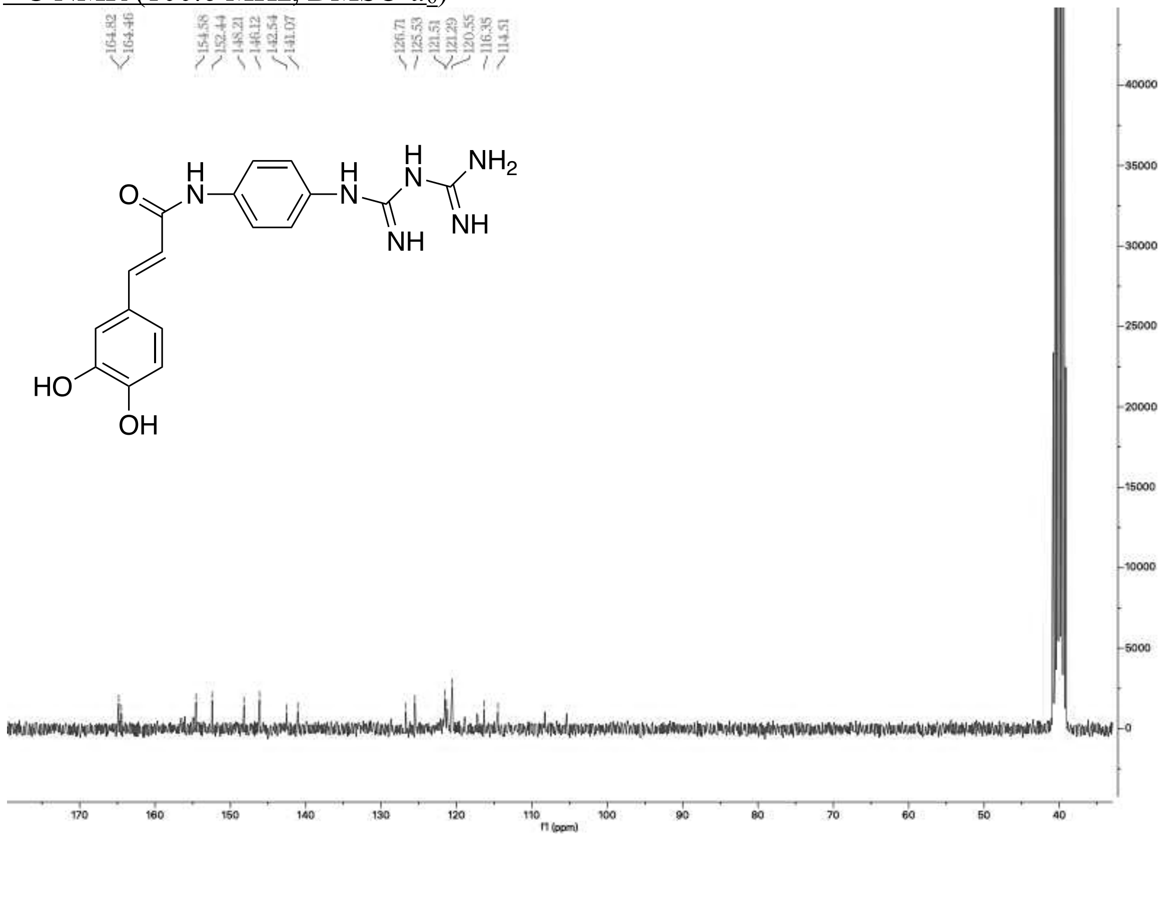

2.21. Spectra of **5f**

^{1}H NMR (400 MHz, DMSO-d_6)

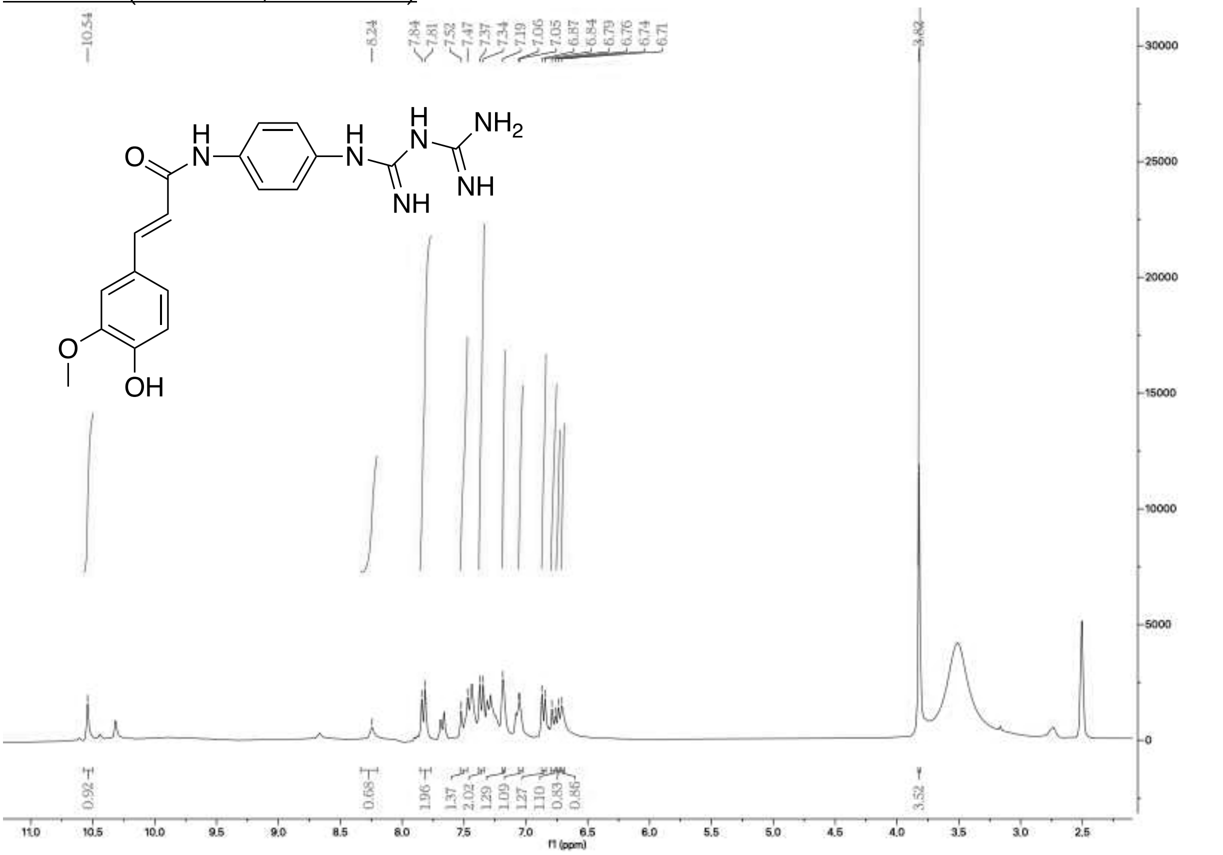

^{13}C NMR (100.6 MHz, DMSO-d_6)

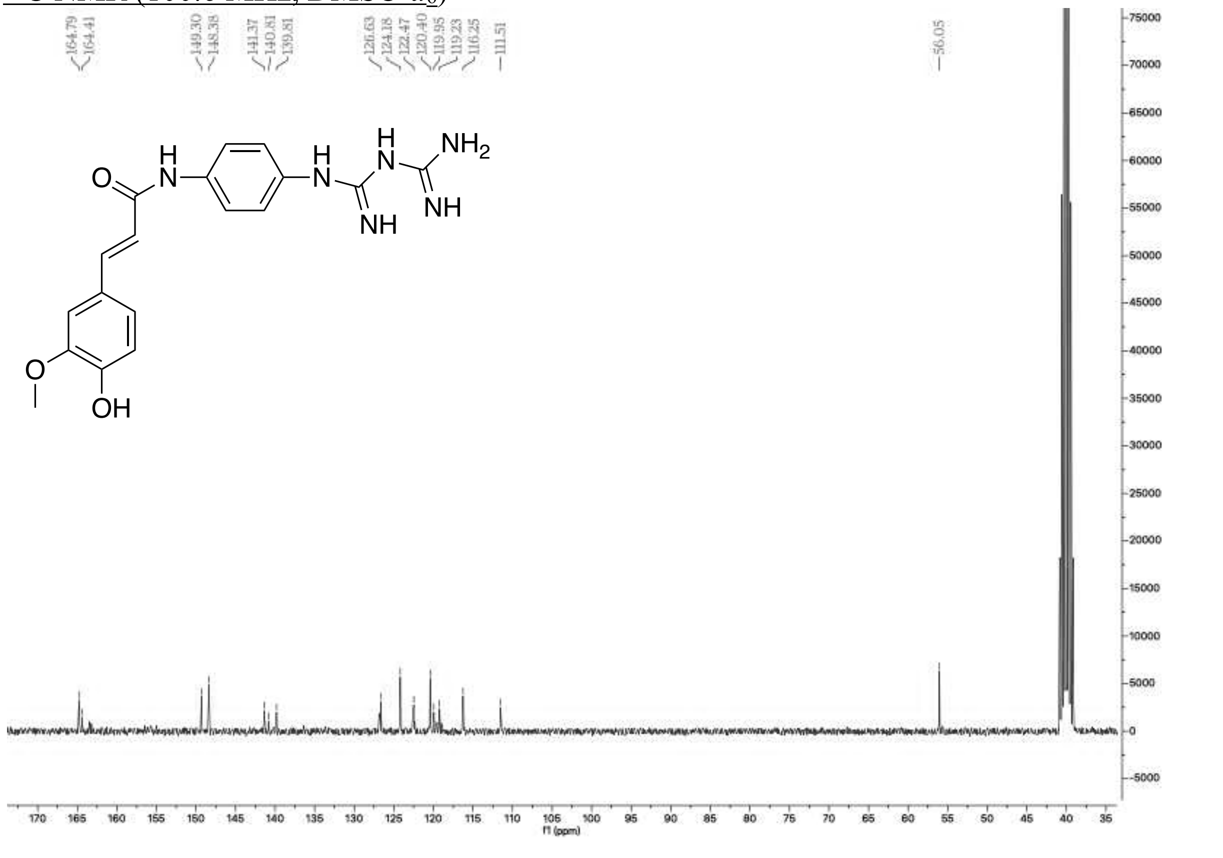

2.22. Spectra of **5g**

^{1}H NMR (400 MHz, DMSO-d_6)

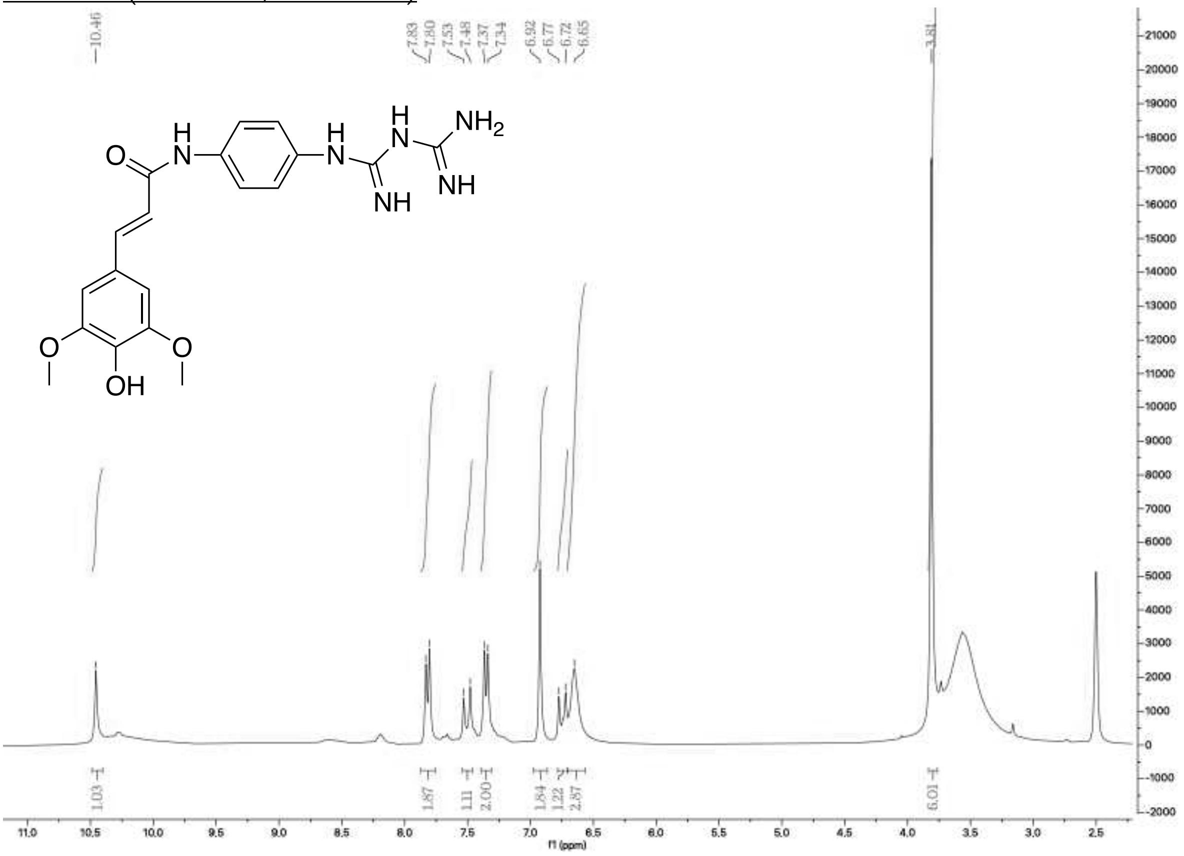

^{13}C NMR (100.6 MHz, DMSO-d_6)

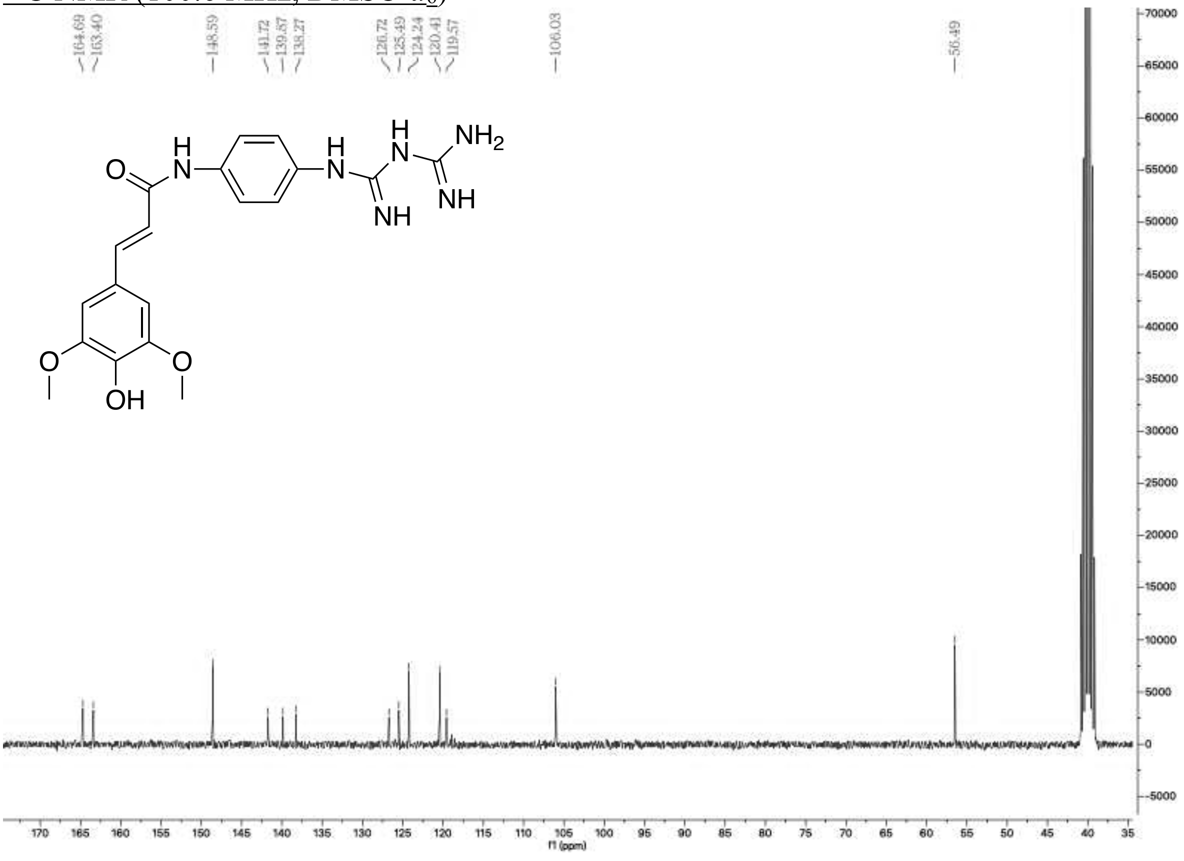

2.23. Spectra of **5h**

^{1}H NMR (300 MHz, DMSO-d_6)

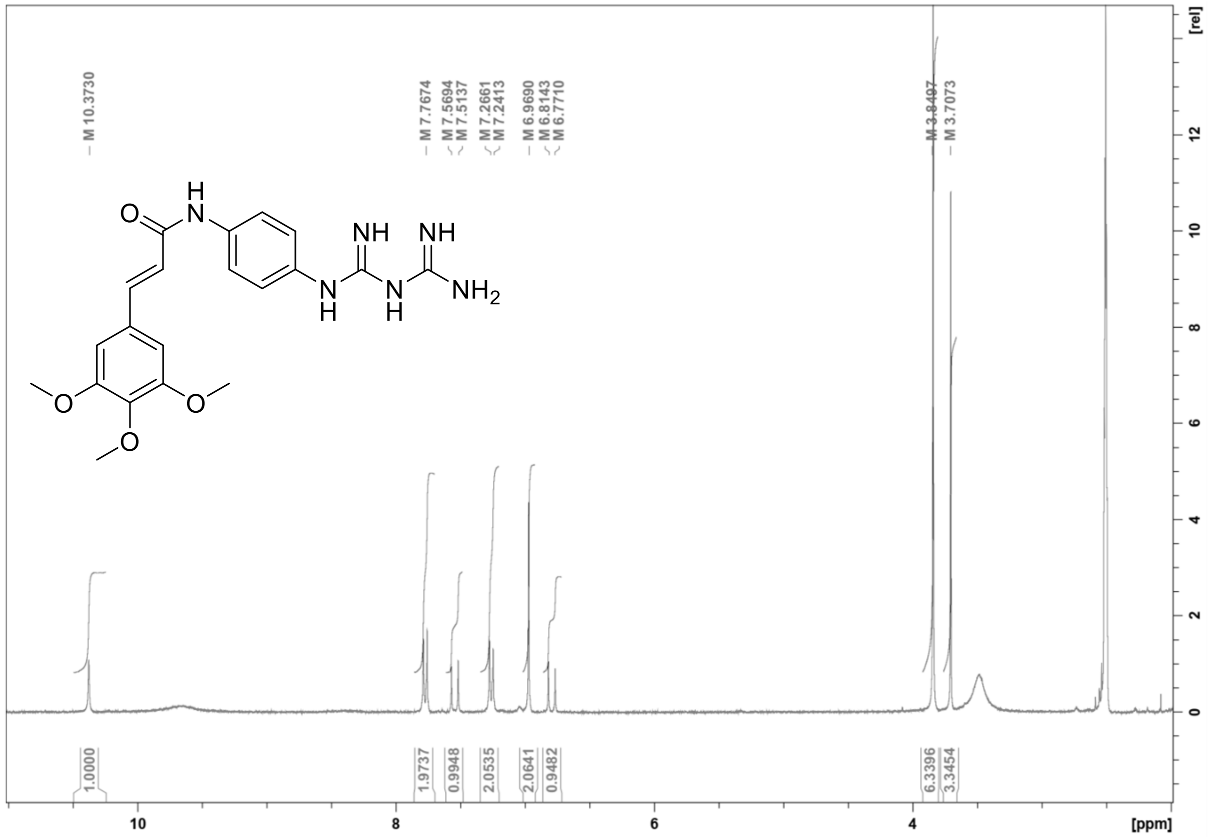

^{13}C NMR (75 MHz, DMSO-d_6)

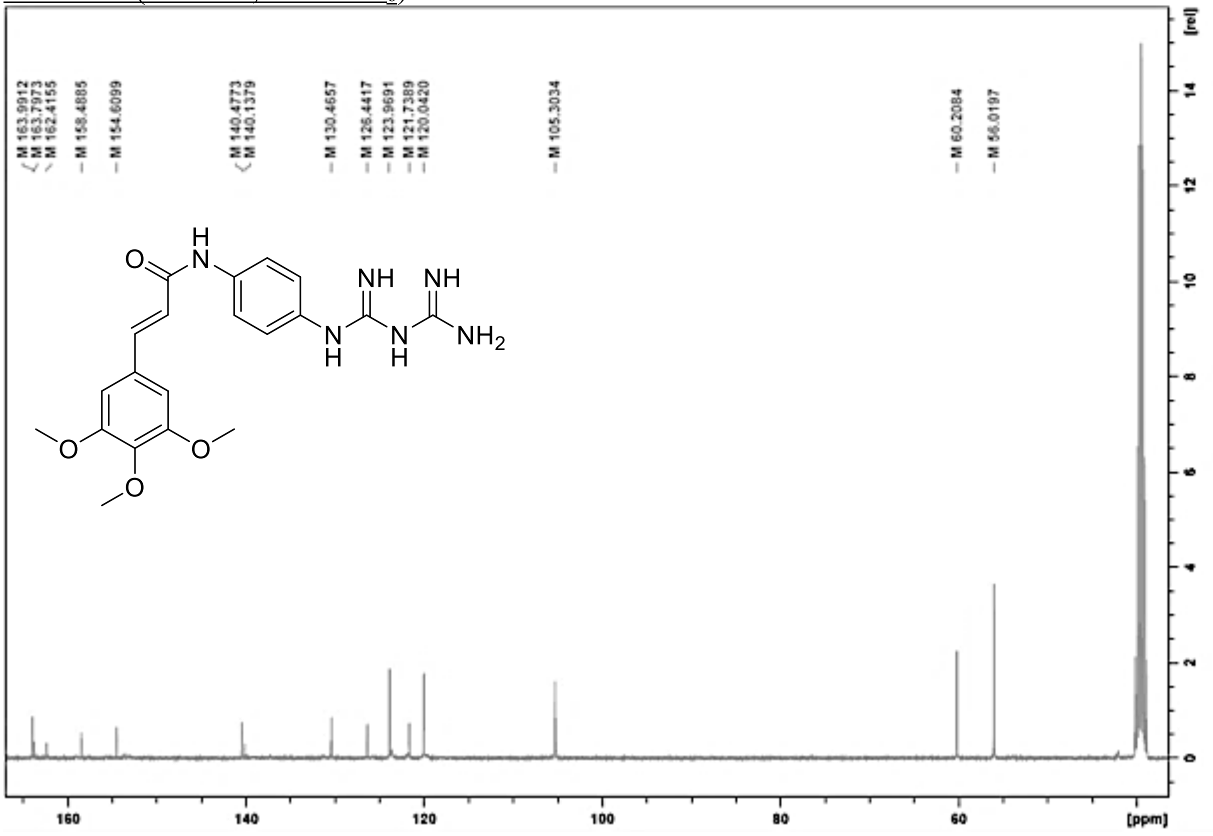

3. Procedure for the synthesis of compounds **6-7**

3.1 2-(4-Isopropoxyphenyl)acetic acid (**6**)

Title compound was obtained by stirring a mixture in DMF (20 mL) of commercial 4-hydroxyphenylacetic acid (2.0 g, 13.2 mmol), K_2CO_3 (6.0 g, 43.4 mmol, 3.3 eq) and 2-bromopropane (5.7 mL, 60.5 mmol, 4.6 eq) for 16 h at 110 °C according to the procedure reported by Pasturel *et al.* [12]. After cooling the reaction mixture to room temperature water (20 mL) was added and the mixture was extracted with ethyl acetate (3 × 30 mL). The gathered organic layers were dried over $MgSO_4$, filtered and concentrated to yield isopropyl 2-(4-isopropoxyphenyl)acetate as a colorless oil (6.2 g, quant). ^1H NMR (300 MHz, $CDCl_3$) δ 7.17 (d, 2H, J = 8.6 Hz, H-2), 6.83 (d, 2H, J = 8.6 Hz, H-3), 5.00 (sept, 1H, J = 6.4 Hz, $CO_2C\textit{H}(CH_3)_2$), 4.51 (sept, 1H, J = 6.1 Hz, $OC\textit{H}(CH_3)_2$), 3.50 (s, 2H, CH_2), 1.32 (d, 6H, J = 6.1 Hz, $OCH(C\textit{H}_3)_2$), 1.22 (d, 6H, J = 6.2 Hz, $CO_2CH(C\textit{H}_3)_2$); ^{13}C NMR (75 MHz, $CDCl_3$) δ 171.4 (C=O), 156.9 (C-4), 130.1 (C-2), 126.2 (C-1), 115.9 (C-3), 69.9 ($O\textit{C}H(CH_3)_2$), 68.0 ($CO_2\textit{C}H(CH_3)_2$), 40.8 (CH_2), 22.0 ($OCH(\textit{C}H_3)_2$), 21.7 ($CO_2CH(\textit{C}H_3)_2$). To perform saponification, the residue, dissolved in water:THF (1:1, v/v) (40 mL), was added NaOH (7.4 g, 185 mmol, 14 eq) and refluxed for 16 h. After gradual cooling of the mixture down to room temperature, then to 0 °C, concentrated HCl was added as to reach pH 1. Ethyl acetate (50 mL) was the added and the aqueous layer was extracted with ethyl acetate (2 × 30 mL). The gathered organic layers were dried over $MgSO_4$, filtered and concentrated. The residue was crystallized in ethyl acetate:n-hexane (1:9, v/v; 20 mL) to give **8**. White crystals (1.31 g, 51%), mp 60 °C. ^1H NMR (300 MHz, DMSO-d_6) δ 7.13 (d, 2H, J = 8.6 Hz, H-2), 6.82 (d, 2H, J = 8.6 Hz, H-3), 4.53 (sept, 1H, J = 6.1 Hz, $C\textit{H}(CH_3)_2$), 3.45 (s, 2H, CH_2), 1.22 (d, 6H, J = 6.1 Hz, $CH(C\textit{H}_3)_2$); ^{13}C NMR (75 MHz, $CDCl_3$) δ 173.6 (C=O), 156.6 (C-4), 130.8 (C-2), 127.1 (C-1), 115.9 (C-3), 70.0 ($\textit{C}H(CH_3)_2$), 40.3 (CH_2), 22.2 ($CH(\textit{C}H_3)_2$). HRMS calc. for $C_{11}H_{15}O_3^+$ $[M+H]^+$ 193.0870, found 193.0868.

3.2 (*E*)-3-(3,5-Diisopropoxyphenyl)-2-(4-isopropoxyphenyl)acrylic acid (**7**)

Compounds **6** (500 mg, 2.58 mmol, 1 eq) and **1d** (572 mg, 2.58 mmol, 1 eq) were mixed under argon in a mixture of acetic anhydride (500 µL) and triethylamine (250 µL) in a sealed tube and stirred at 100 °C for 1 day [13]. The solution was allowed to cool to room temperature and diluted with ethyl acetate (10 mL). The organic layer was washed with water (2 × 10 mL) and brine (2 × 10 mL). The organic layer was dried over $MgSO_4$, filtered and concentrated and the residue was dissolved in acetic acid (10 mL). The solution was poured over a mixture of water and ice (20 g / 20g) and the precipitate was filtered off to yield a yellow powder (700 mg, 68%), mp 166 °C. ^1H NMR (400 MHz, DMSO-d_6) δ 12.7 (bs, 1H, CO_2H), 7.61 (s, 1H, $=CH_{vinylic}$), 7.06 (d, 2H, J = 6.9 Hz, $H_{\textit{ortho}\ monosubstituted}$), 6.93 (d, 2H, J = 6.9 Hz, $H_{\textit{meta}\ monosubstituted}$), 6.26 (m, 1H, $H_{\textit{para}\ disubstituted}$), 6.23 (m, 2H, $H_{\textit{ortho}\ disubstituted}$); 4.61 (sept, 1H, J = 6.0 Hz, $C\textit{H}(CH_3)_2$), 4.22 (sept, 2 H, J = 6.0 Hz, 2 × $C\textit{H}(CH_3)_2$), 1.28 (d, 6 H, 1 H, J = 5.90 Hz, $CH(C\textit{H}_3)_2$), 1.10 (d, 12 H, J = 6.1 Hz, 2 × $CH(C\textit{H}_3)_2$); ^{13}C NMR (100 MHz, DMSO-d_6) δ 169.0 (C=O), 158.6 ($C_{\textit{meta}\ disubstituted}$), 157.4 ($C_{\textit{para}\ monosubstituted}$), 139.4 ($\textit{C}H$=C), 136.8 ($C_{\textit{ipso}\ disubstituted}$), 133.6 (C=$\textit{C}$H), 131.1 ($C_{\textit{ortho}\ monosubstituted}$), 128.7 ($C_{\textit{ipso}\ monosubstituted}$), 115.9 ($C_{\textit{meta}\ monosubstituted}$), 109.7 ($C_{\textit{ortho}\ disubstituted}$), 105.2 ($C_{\textit{para}\ disubstituted}$), 69.6 ($\textit{C}H(CH_3)_2$), 69.5 ($\textit{C}H(CH_3)_2$), 22.3 ($CH(\textit{C}H_3)_2$), 22.2 ($CH(\textit{C}H_3)_2$). HRMS calc. for $C_{24}H_{29}O_5^-$ $[M-H]^-$ 397.1993, found 397.1995.

4. NMR spectra (^{1}H and ^{13}C) of metformin-resveratrol hybrids

4.1 Spectra of **8**

^{1}H NMR (400 MHz, DMSO-*d*6)

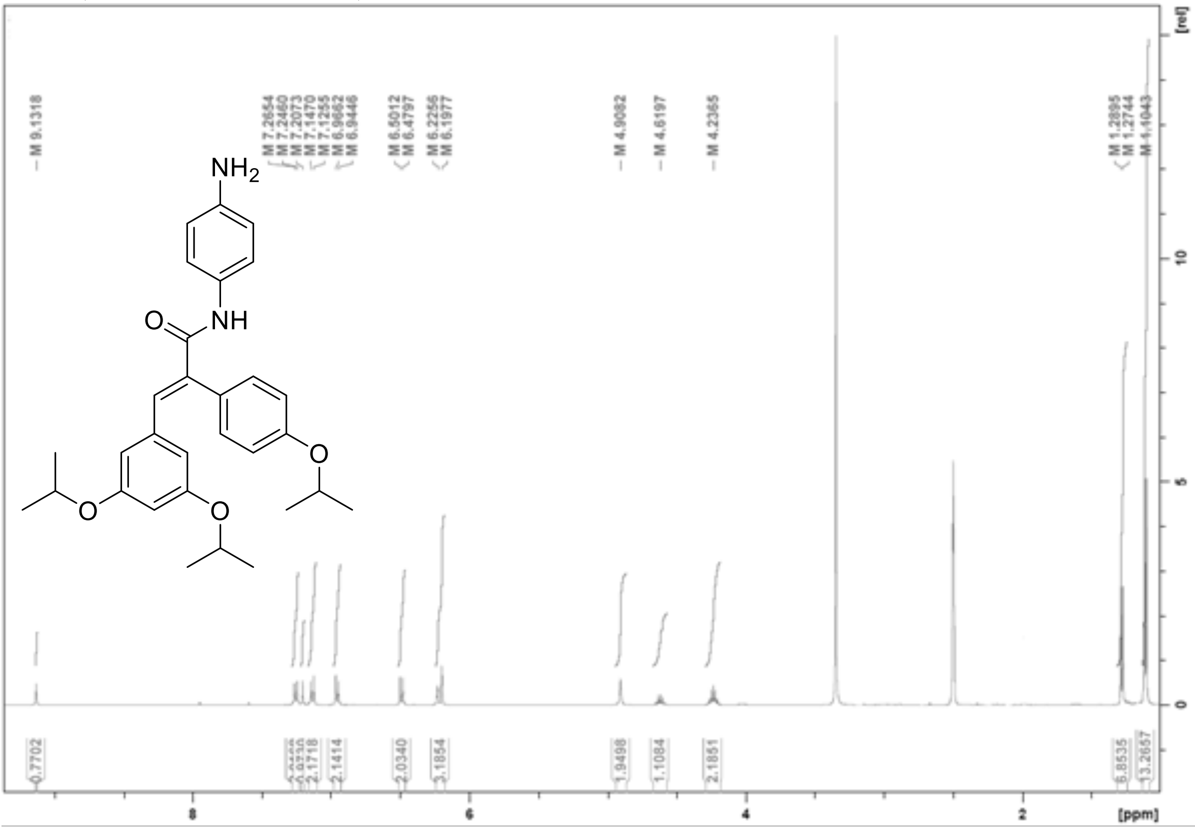

^{13}C NMR (100.6 MHz, DMSO-*d*6)

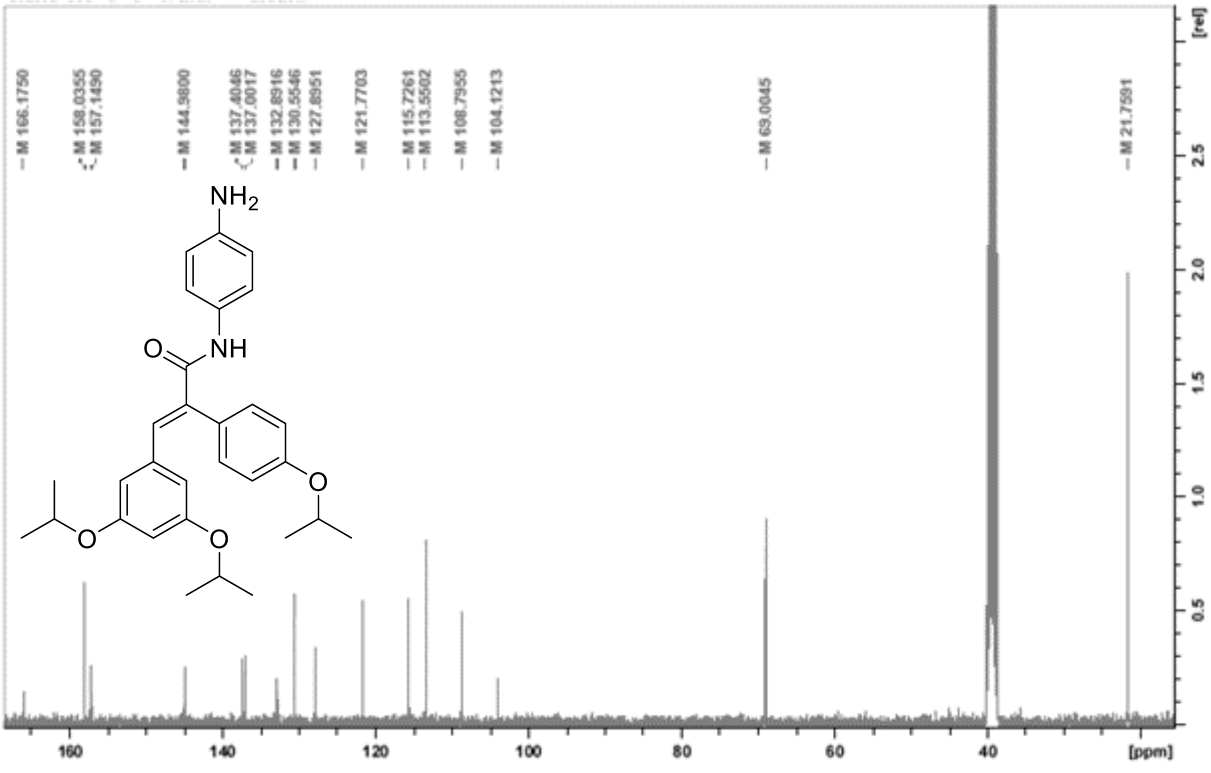

4.2 Spectra of **9**

^{1}H NMR (400 MHz, DMSO-d_6)

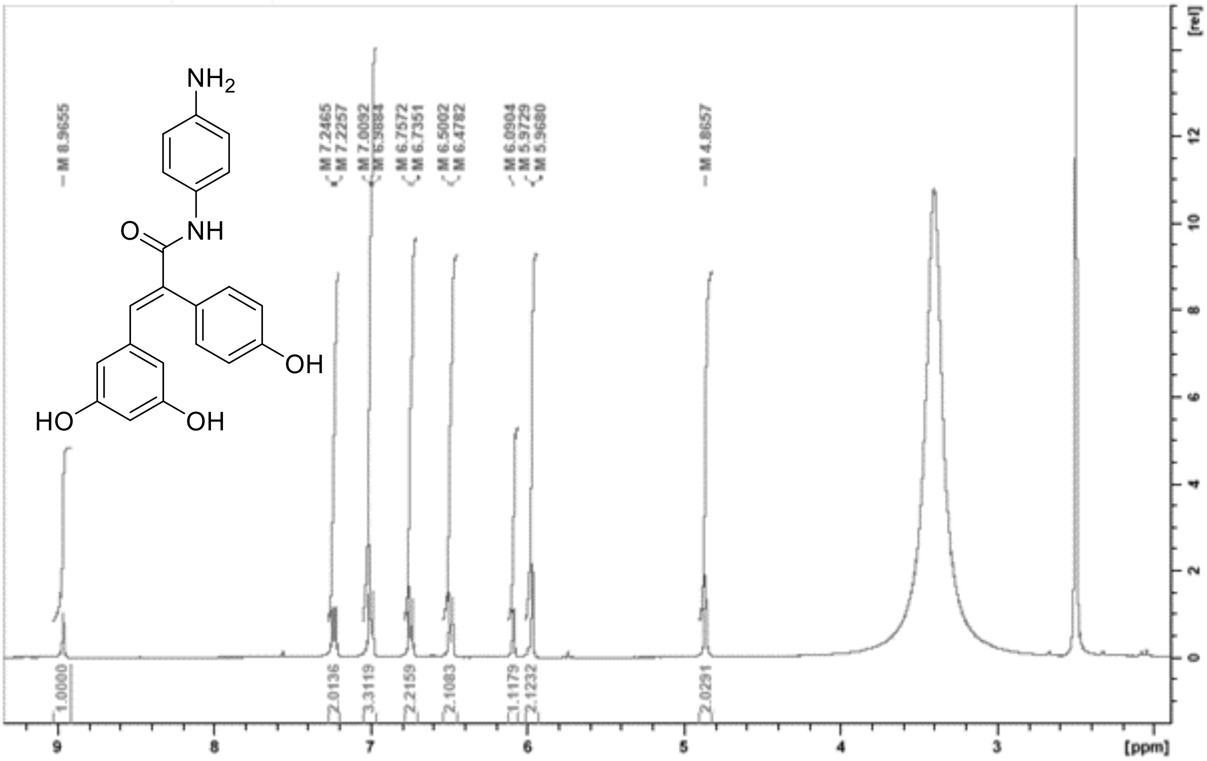

^{13}C NMR (100.6 MHz, DMSO-d_6)

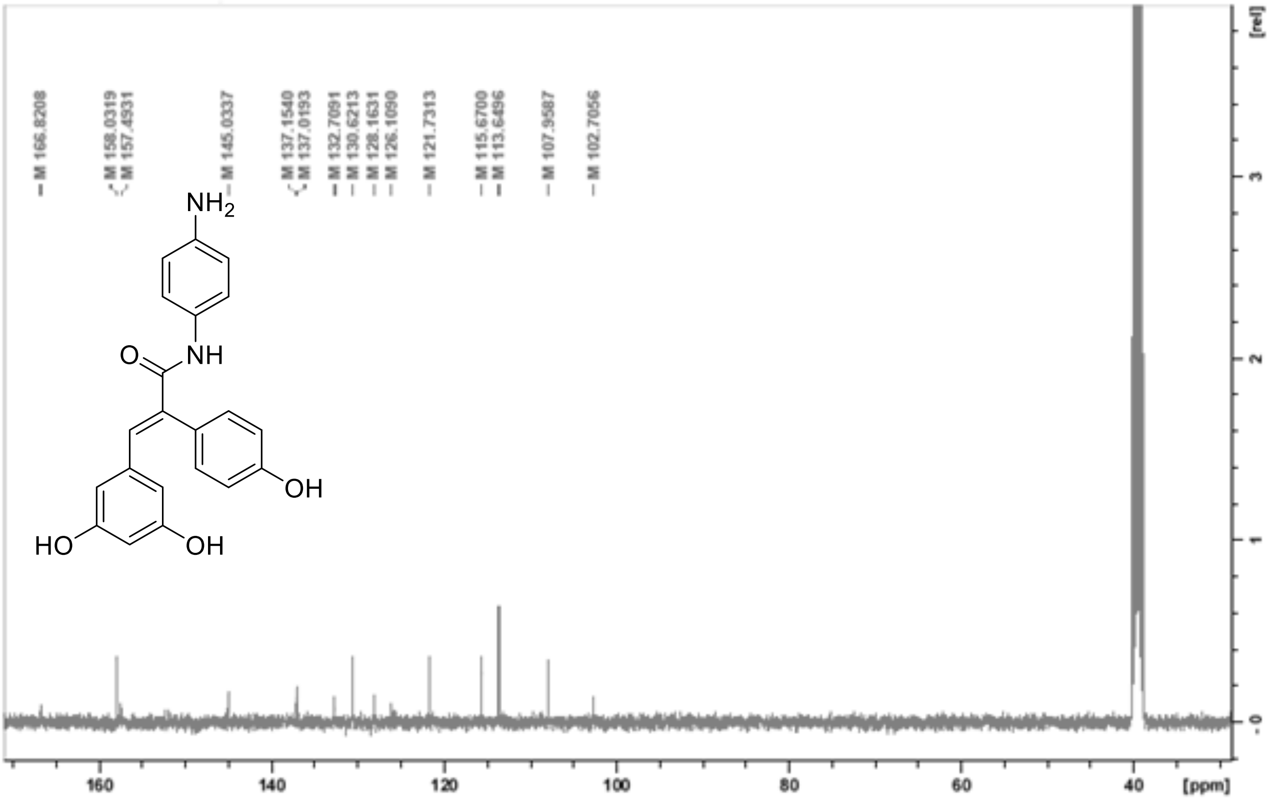

4.3 Spectra of **10**

^{1}H NMR (400 MHz, DMSO-d_6)

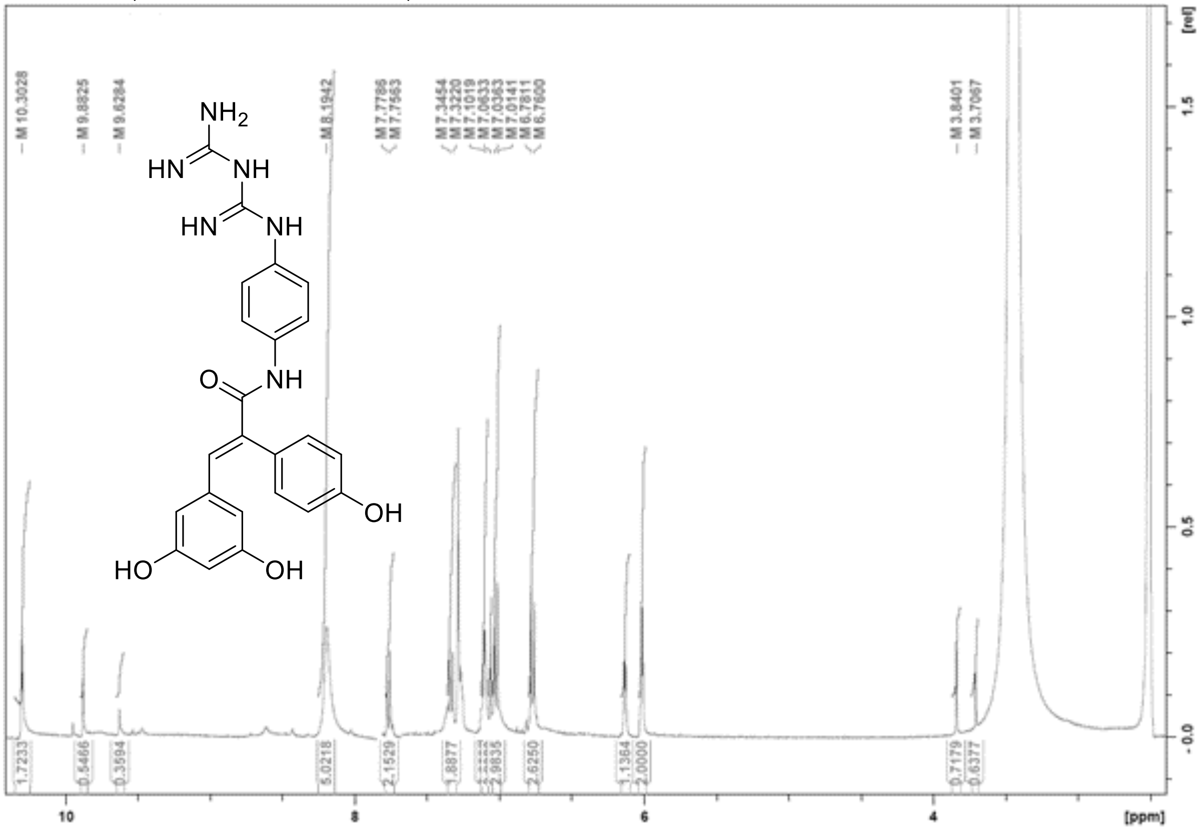

^{13}C NMR (100.6 MHz, DMSO-d_6)

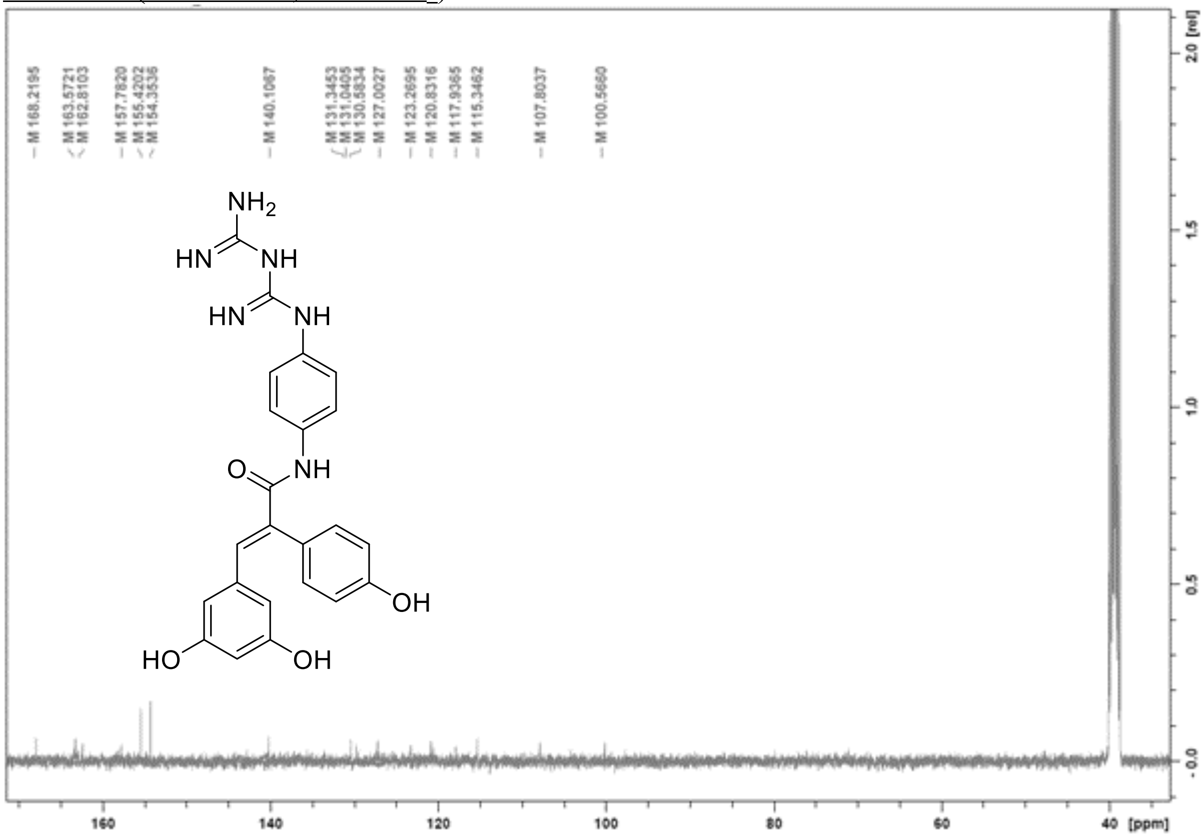

5. Calculated AlogP values for metformin, phenolic acids and hybrid derivatives (**Table S1**)

Compound	AlogP[b]
5a	1.64
5b	1.66
5c	1.66
5d	1.32
5e	1.34
5f	1.77
5g	1.90
5h	2.07
10	3.12
metformin	-1.41
caffeic acid	1.67
ferulic acid	1.58
sinapic acid	1.63
salicylic acid	1.96
gallic acid	1.17

Table S1. Predicted lipophilicity (AlogP) values of metformin, phenolic acids, and hybrid compounds (**5a-5h, 10**) were determined using the Virtual Computational Chemistry Laboratory software at ALOGPS 2.1 homepage available online at www.vcclab.org/lab/alogps/ (accessed on November 3rd 2025).

6. Impact of a 4 h treatment with metformin, **5a** or **5h** on serine threonine kinase (STK) and tyrosine kinase (PTK) activity as compared to control (DMSO 0.01%) using kinomic Pamgene assay.

6.1 List of kinases significantly inhibited or activated by metformin in GBM9 and U87 cells (**Table S2**)

Table S2 summarizes the kinases identified as significantly modulated following treatment with metformin, based on PamGene kinase activity profiling. Kinase activity (setStat) and specificity (pSpecificityScore) values were derived from the BioNavigator analysis pipeline. Kinases with pSpecificityScore < 0.05 were considered significantly altered. Positive setStat values indicate kinase activation, while negative values correspond to inhibition. Data are presented separately for U87 differentiated cells and GBM9 stem-like cells, highlighting distinct signaling reprogramming patterns induced by metformin.

Kinases inhibited by Metformin in U87											
**kinase Name**	Normalized.Kinase.Stat	specificityScore	sensitivityScore	setStat	Peptides.in.Set	pSpecificityScore	pSensitivityScore	finalScore	Cell	type	gene
**ANPa (NPR1)**	-0.0649710165101066	0.918	NA	-0.58473914859096	9	0.0371573187987575	NA	0.0371573187987575	U87	STK	ANPNPR1
**CDK2**	-0.0329823467483218	0.97	NA	-0.527717547973149	16	0.0132282657337552	NA	0.0132282657337552	U87	STK	CDK2
**ERK1**	-0.026023938	1	NA	-0.546502715	21	8.69458712628891e-05	NA	0	U87	ST	ERK1

	8564106			984622						K	
**ERK2 (MAPK1)**	-0.0204537454465023	0.996	NA	-0.409074908930047	20	0.00174066157630127	NA	0.00174066157630127	U87	STK	ERK2MAPK1
**ERK5 (MAPK7)**	-0.0409361574093059	0.978	NA	-0.6959146759582	17	0.00966114521239857	NA	0.00966114521239857	U87	STK	ERK5MAPK7
**IKKb (IKBKINASE)**	-0.00336248535242434	0.996	NA	-0.00672497070484868	2	0.00174066157630127	NA	0.00174066157630127	U87	STK	IKKIKBKINASE
**MAK**	-0.0101577830140023	0.988	NA	-0.060946698084014	6	0.00524305541237188	NA	0.00524305541237188	U87	STK	MAK
**ZC4**	-0.00434827358572437	0.99	NA	-0.00869654717144874	2	0.00436480540245009	NA	0.00436480540245009	U87	STK	ZC4
Kinases activated by Metformin in U87											
**kinase Name**	Normalized.Kinase.Stat	specificityScore	sensitivityScore	setStat	Peptides.in.Set	pSpecificityScore	pSensitivityScore	finalScore	Cell	type	gene
**ALK**	5.62331101596251	0.892	NA	61.8564211755876	11	0.0496351456238769	NA	0.0496351456238769	U87	PTK	ALK
**FLT1**	3.20579297846675	0.916	NA	6.41158595693351	2	0.0381045263321496	NA	0.0381045263321496	U87	PTK	FLT1
**LTK**	4.84585748201164	0.926	NA	33.9210023740815	7	0.0333890133180656	NA	0.0333890133180656	U87	PTK	LTK
**MER (MERTK)**	5.42576124943797	0.906	NA	59.6833737438177	11	0.0428718023231869	NA	0.0428718023231869	U87	PTK	MERMERTK
**PYK2 (PTK2B)**	5.53780077377327	0.898	NA	55.3780077377327	10	0.0467236633326956	NA	0.0467236633326956	U87	PTK	PYK2PTK2B
**TRKA (NTRK1)**	3.15809239900877	0.944	NA	9.4742771970263	3	0.025028005701931	NA	0.025028005701931	U87	PTK	TRKANTRK1
**TRKB (NTRK2)**	3.02420911990388	0.998	NA	12.0968364796155	4	0.000869458712628891	NA	0.000869458712628891	U87	PTK	TRKBNTRK2
**TRKC (NTRK3)**	3.34312975103838	0.97	NA	13.3725190041535	4	0.0132282657337552	NA	0.0132282657337552	U87	PTK	TRKCNTRK3
**CDK6**	0.0515209048325717	0.906	NA	0.154562714497715	3	0.0428718023231869	NA	0.0428718023231869	U87	STK	CDK6
**ERK7 (BI916334)**	0.0390653710368892	0.934	NA	0.156261484147557	4	0.0296531237699066	NA	0.0296531237699066	U87	STK	ERK7BI916334
**ICK**	0.0176503310857877	0.972	NA	0.0706013243431508	4	0.0123337350737254	NA	0.0123337350737254	U87	STK	ICK
**JNK1 (MAPK8)**	0.126956311266079	0.926	NA	2.4121699140555	19	0.0333890133180656	NA	0.0333890133180656	U87	STK	JNK1MAPK8
**JNK2 (MAPK9)**	0.0215011518752431	0.992	NA	0.322517278128647	15	0.00348832784582135	NA	0.00348832784582135	U87	STK	JNK2MAPK9
**JNK3**	0.126956311	0.926	NA	2.412169914	19	0.033389013	NA	0.033389013	U8	S	JNK3MA

**(MAPK 10)**	266079			0555		3180656		3180656	7	TK	PK10
**p38a MAPK (MAPK 14)**	0.113847721961469	0.894	NA	1.82156355138351	16	0.0486624812040823	NA	0.0486624812040823	U87	STK	38MAPK MAPK14
**p38b MAPK (MAPK 11)**	0.0129705425251843	0.998	NA	0.181587595352581	14	0.000869458712628891	NA	0.000869458712628891	U87	STK	38MAPK MAPK11
**p38d MAPK (MAPK 13)**	0.124958438282519	0.928	NA	2.49916876565039	20	0.0324520237811379	NA	0.0324520237811379	U87	STK	38MAPK MAPK13
Kinases inhibited by Metformin in GBM											
**kinase Name**	Normalized.Kinase.Stat	specificityScore	sensitivityScore	setStat	Peptides.in.Set	pSpecificityScore	pSensitivityScore	finalScore	Cell	type	gene
**CHED (CDC2L5)**	-0.0213321333003678	0.94	NA	-0.0426642666007356	2	0.0268721464003014	NA	0.0268721464003014	GBM	STK	CHEDCDC2L5
**ERK7 (BI916334)**	-0.0213321333003678	0.94	NA	-0.0426642666007356	2	0.0268721464003014	NA	0.0268721464003014	GBM	STK	ERK7BI916334
Kinases activated by Metformin in GBM											
**kinase Name**	Normalized.Kinase.Stat	specificityScore	sensitivityScore	setStat	Peptides.in.Set	pSpecificityScore	pSensitivityScore	finalScore	Cell	type	gene
**CTK (MATK)**	0.130896244730268	0.928	NA	1.83254742622375	14	0.0324520237811379	NA	0.0324520237811379	GBM	PTK	CTKMATK
**FGR**	0.10288342833519	0.944	NA	0.823067426681519	8	0.025028005701931	NA	0.025028005701931	GBM	PTK	FGR
**FRK**	0.138513072331746	0.962	NA	2.77026144663493	20	0.0168249279621871	NA	0.0168249279621871	GBM	PTK	FRK
**HCK**	0.106472253799438	0.994	NA	1.59708380699158	15	0.00261361560268669	NA	0.00261361560268669	GBM	PTK	HCK
**LYN**	0.0998614773605809	0.976	NA	1.09847625096639	11	0.0105501823333082	NA	0.0105501823333082	GBM	PTK	LYN
**SRC**	0.117420679046994	0.964	NA	1.64388950665792	14	0.0159229660971692	NA	0.0159229660971692	GBM	PTK	SRC
**YES1**	0.124935969328269	0.93	NA	1.6241676012675	13	0.0315170514460649	NA	0.0315170514460649	GBM	PTK	YES1
**ANPa (NPR1)**	0.0410050215780064	0.916	NA	0.287035151046045	7	0.0381045263321496	NA	0.0381045263321496	GBM	STK	ANPNPR1
**ATR**	0.00300404656843248	0.996	NA	0.00901213970529744	3	0.00174066157630127	NA	0.00174066157630127	GBM	STK	ATR
**RAF1**	0.0168537013839605	0.964	NA	0.033707402767921	2	0.0159229660971692	NA	0.0159229660971692	GBM	STK	RAF1

6.2 List of kinases significantly inhibited or activated by **5a** in GBM9 and U87 cells (**Table S3**)

Table S3 summarizes the kinases identified as significantly modulated following treatment with compound **5a**, based on PamGene kinase activity profiling. Kinase activity (setStat) and specificity (pSpecificityScore) values were derived from the BioNavigator analysis pipeline. Kinases with pSpecificityScore < 0.05 were considered significantly altered. Positive setStat values indicate kinase activation, while negative values correspond to inhibition. Data are presented separately for U87 differentiated cells and GBM9 stem-like cells, highlighting distinct signaling reprogramming patterns induced by **5a**.

Kinases inhibited by 5a in U87											
**kinaseName**	Normalized.Kinase.Stat	specificityScore	sensitivityScore	setStat	Peptides.in.Set	pSpecificityScore	pSensitivityScore	finalScore	Cell	type	gene
**FLT1**	-3.2705279291147	0.934	NA	-6.54105585822939	2	0.0296531237699066	NA	0.0296531237699066	U87	PTK	FLT1
**LTK**	-4.46528951342754	0.922	NA	-31.2570265939928	7	0.0352690789463706	NA	0.0352690789463706	U87	PTK	LTK
**RON**	-2.50336034248613	0.964	NA	-2.50336034248613	1	0.0159229660971692	NA	0.0159229660971692	U87	PTK	RON
**TRKA (NTRK1)**	-3.34168451185104	0.97	NA	-10.0250535355531	3	0.0132282657337552	NA	0.0132282657337552	U87	PTK	TRKANTRK1
**TRKB (NTRK2)**	-3.30687842854596	0.992	NA	-13.2275137141838	4	0.00348832784582135	NA	0.00348832784582135	U87	PTK	TRKBNTRK2
**TRKC (NTRK3)**	-3.59853668569017	0.978	NA	-14.3941467427607	4	0.00966114521239857	NA	0.00966114521239857	U87	PTK	TRKCNTRK3
**ANPb (NPR2)**	-0.0326199060755653	0.986	NA	-0.0326199060755653	1	0.0061230850587888	NA	0.0061230850587888	U87	STK	ANPNPR2
**CDK2**	-0.0972268471369751	0.976	NA	-1.26394901278068	13	0.0105501823333082	NA	0.0105501823333082	U87	STK	CDK2
**CDK7**	-0.0430591381252201	0.97	NA	-0.258354828751321	6	0.0132282657337552	NA	0.0132282657337552	U87	STK	CDK7
**CDKL4 (AA626859)**	-0.0865356248286718	0.942	NA	-0.778820623458046	9	0.0259490972071227	NA	0.0259490972071227	U87	STK	CDKL4AA626859
**CDKL5 (STK9)**	-0.0844812136170159	0.96	NA	-0.675849708936128	8	0.0177287669604316	NA	0.0177287669604316	U87	STK	CDKL5STK9
**DAPK2**	-0.0326199060755653	0.986	NA	-0.0326199060755653	1	0.0061230850587888	NA	0.0061230850587888	U87	STK	DAPK2
**ERK1**	-0.0821866207755805	0.99	NA	-1.31498593240929	16	0.00436480540245009	NA	0.00436480540245009	U87	STK	ERK1
**ERK2 (MAPK1)**	-0.0854910684222482	0.992	NA	-1.28236602633372	15	0.00348832784582135	NA	0.00348832784582135	U87	STK	ERK2MAPK1
**IKKE**	-	0.986	NA	-	1	0.006123085	NA	0.006123085	U8	S	IKKE

	0.0326199060755653			0.0326199060755653		0587888		0587888	7	TK	
**JNK1 (MAPK8)**	-0.135260562305085	0.98	NA	-2.16416899688136	16	0.00877392430750515	NA	0.00877392430750515	U87	STK	JNK1MAPK8
**JNK2 (MAPK9)**	-0.0621000801547809	0.99	NA	-0.74520096185737	12	0.00436480540245009	NA	0.00436480540245009	U87	STK	JNK2MAPK9
**JNK3 (MAPK10)**	-0.135260562305085	0.98	NA	-2.16416899688136	16	0.00877392430750515	NA	0.00877392430750515	U87	STK	JNK3MAPK10
**PIM2**	-0.191245027974222	0.97	NA	-3.05992044758756	16	0.0132282657337552	NA	0.0132282657337552	U87	STK	PIM2
**RAF1**	-0.0523354558900755	0.936	NA	-0.104670911780151	2	0.0287241512618947	NA	0.0287241512618947	U87	STK	RAF1
**TBK1**	-0.0326199060755653	0.986	NA	-0.0326199060755653	1	0.0061230850587888	NA	0.0061230850587888	U87	STK	TBK1
**p38a MAPK (MAPK14)**	-0.0525274365859052	0.988	NA	-0.682856675616768	13	0.00524305541237188	NA	0.00524305541237188	U87	STK	38MAPKMAPK14
**p38d MAPK (MAPK13)**	-0.114955641012914	0.984	NA	-1.83929025620662	16	0.00700490156865849	NA	0.00700490156865849	U87	STK	38MAPKMAPK13
**p38g MAPK (MAPK12)**	-0.0798100602679163	0.982	NA	-0.957720723214995	12	0.00788851221305034	NA	0.00788851221305034	U87	STK	38MAPKMAPK12
Kinases activated by 5a in U87											
**kinaseName**	Normalized.Kinase.Stat	specificityScore	sensitivityScore	setStat	Peptides.in.Set	pSpecificityScore	pSensitivityScore	finalScore	Cell	type	gene
**ADCK3 (LOC56997)**	0.0851088423220755	0.928	NA	0.425544211610377	5	0.0324520237811379	NA	0.0324520237811379	U87	STK	ADCK3LOC56997
**ANPa (NPR1)**	0.0435768701132563	0.982	NA	0.435768701132563	10	0.00788851221305034	NA	0.00788851221305034	U87	STK	ANPNPR1
**CDK1 (CDC2)**	0.116499595394615	0.948	NA	1.16499595394615	10	0.0231916626619338	NA	0.0231916626619338	U87	STK	CDK1CDC2
**CDK3**	0.144395744781907	0.892	NA	0.866374468691441	6	0.0496351456238769	NA	0.0496351456238769	U87	STK	CDK3
**ERK5 (MAPK7)**	0.0203003862590553	0.996	NA	0.284205407626774	14	0.00174066157630127	NA	0.00174066157630127	U87	STK	ERK5MAPK7
**ERK7 (BI916334)**	0.0278347611096422	0.966	NA	0.111339044438569	4	0.0150228735845067	NA	0.0150228735845067	U87	STK	ERK7BI916334
**ICK**	0.0849962704041364	0.92	NA	0.254988811212409	3	0.0362121726544447	NA	0.0362121726544447	U87	STK	ICK
**MAK**	0.0279223775141202	0.976	NA	0.111689510056481	4	0.0105501823333082	NA	0.0105501823333082	U87	STK	MAK
**SCYL2**	0.037427941	0.964	NA	0.037427941	1	0.015922966	NA	0.015922966	U8	S	SCYL2FL

**(FLJ10074)**	8190364			8190364		0971692		0971692	7	TK	J10074
**p38b MAPK (MAPK11)**	0.0837028041956148	0.978	NA	0.920730846151763	11	0.00966114521239857	NA	0.00966114521239857	U87	STK	38MAPK MAPK11
Kinases inhibited by 5a in GBM											
**kinaseName**	Normalized.Kinase.Stat	specificityScore	sensitivityScore	setStat	Peptides.in.Set	pSpecificityScore	pSensitivityScore	finalScore	Cell	type	gene
**FLT3**	-0.496351040065407	0.9	NA	-2.48175520032703	5	0.0457574905606751	NA	0.0457574905606751	GBM	PTK	FLT3
**JAK1_d2**	-0.39652242472279	0.978	NA	-0.39652242472279	1	0.00966114521239857	NA	0.00966114521239857	GBM	PTK	JAK12
**JAK2**	-0.459989070729029	0.932	NA	-0.919978141458058	2	0.0305840876460186	NA	0.0305840876460186	GBM	PTK	JAK2
**ROR1**	-0.39652242472279	0.978	NA	-0.39652242472279	1	0.00966114521239857	NA	0.00966114521239857	GBM	PTK	ROR1
**ROR2**	-0.39652242472279	0.978	NA	-0.39652242472279	1	0.00966114521239857	NA	0.00966114521239857	GBM	PTK	ROR2
**SRM (SRMS)**	-0.509678457647769	0.952	NA	-6.11614149177323	12	0.0213630516155257	NA	0.0213630516155257	GBM	PTK	SRMSRMS
**MAPKAPK3**	-0.0875714401259882	0.92	NA	-1.4887144821418	17	0.0362121726544447	NA	0.0362121726544447	GBM	STK	MAPKAPK3
**PKACa (PRKACA)**	-0.102269334973334	0.976	NA	-2.76127204428003	27	0.0105501823333082	NA	0.0105501823333082	GBM	STK	PKACPRKACA
**PKACb PRKACB**	-0.111905025150604	0.91	NA	-2.79762562876509	25	0.0409586076789064	NA	0.0409586076789064	GBM	STK	PKACPRKACB
**RSK4 (RPS6KA6)**	-0.0926795668276001	0.902	NA	-1.4828730692416	16	0.0447934624580583	NA	0.0447934624580583	GBM	STK	RSK4RPS6KA6
**mTOR/FRAP**	-0.0229807534028989	0.936	NA	-0.0689422602086966	3	0.0287241512618947	NA	0.0287241512618947	GBM	STK	TORFRAP
Kinases activated by 5a in GBM											
**kinaseName**	Normalized.Kinase.Stat	specificityScore	sensitivityScore	setStat	Peptides.in.Set	pSpecificityScore	pSensitivityScore	finalScore	Cell	type	gene
**CK1a1 (CSNK1A1)**	0.00490767120435498	1	NA	0.00490767120435498	1	0.000174066157630127	NA	0	GBM	STK	CK11CSNK1A1
**CK2a1 (CSNK2A1)**	0.00490767120435498	1	NA	0.00490767120435498	1	0.000174066157630127	NA	0	GBM	STK	CK21CSNK2A1
**CK2a2 (CSNK2A2)**	0.00490767120435498	1	NA	0.00490767120435498	1	0.000174066157630127	NA	0	GBM	STK	CK22CSNK2A2
**CLK3**	0.00490767120435498	1	NA	0.00490767120435498	1	0.000174066157630127	NA	0	GBM	STK	CLK3
**GPRK4**	0.004907671	1	NA	0.004907671	1	0.000174066	NA	0	G	S	GPRK4GP

**(GPRK2L)**	20435498			20435498		157630127			BM	TK	RK2L
**HSER (GUCY2C)**	0.004907671 20435498	1	NA	0.004907671 20435498	1	0.000174066 157630127	NA	0	GBM	STK	HSERGUCY2C
**IKKa (CHUK)**	0.004907671 20435498	1	NA	0.004907671 20435498	1	0.000174066 157630127	NA	0	GBM	STK	IKKCHUK
**NEK10**	0.004907671 20435498	1	NA	0.004907671 20435498	1	0.000174066 157630127	NA	0	GBM	STK	NEK10
**Nuak2 (DKFZP434J037)**	0.004907671 20435498	1	NA	0.004907671 20435498	1	0.000174066 157630127	NA	0	GBM	STK	N2DKFZP434J037
**PCTAIRE1 (PCTK1)**	0.004907671 20435498	1	NA	0.004907671 20435498	1	0.000174066 157630127	NA	0	GBM	STK	PCTAIRE1 PCTK1
**PCTAIRE2 (PCTK2)**	0.004907671 20435498	1	NA	0.004907671 20435498	1	0.000174066 157630127	NA	0	GBM	STK	PCTAIRE2 PCTK2
**PCTAIRE3**	0.004907671 20435498	1	NA	0.004907671 20435498	1	0.000174066 157630127	NA	0	GBM	STK	PCTAIRE3
**PFTAIRE1 (PFTK1)**	0.004907671 20435498	1	NA	0.004907671 20435498	1	0.000174066 157630127	NA	0	GBM	STK	PFTAIRE1 PFTK1
**PINK1**	0.004907671 20435498	1	NA	0.004907671 20435498	1	0.000174066 157630127	NA	0	GBM	STK	PINK1
**SPEG (KIAA1297)**	0.004907671 20435498	1	NA	0.004907671 20435498	1	0.000174066 157630127	NA	0	GBM	STK	SPEGKIAA1297

6.3 List of kinases significantly inhibited or activated by **5h** in GBM9 and U87 cells (**Table S4**)

Table S4 summarizes the kinases identified as significantly modulated following treatment with compound **5h**, based on PamGene kinase activity profiling. Kinase activity (setStat) and specificity (pSpecificityScore) values were derived from the BioNavigator analysis pipeline. Kinases with pSpecificityScore < 0.05 were considered significantly altered. Positive setStat values indicate kinase activation, while negative values correspond to inhibition. Data are presented separately for U87 differentiated cells and GBM9 stem-like cells, highlighting distinct signaling reprogramming patterns induced by **5h**.

Kinases inhibited by 5h in U87											
**kinaseName**	Normalized.Kinase.Stat	specificityScore	sensitivityScore	setStat	Peptides.in.Set	pSpecificityScore	pSensitivityScore	finalScore	Cell	type	gene
**ERBB2**	-3.9156835270675	0.942	NA	-19.5784176353375	5	0.0259490972071227	NA	0.0259490972071227	U87	PTK	ERBB2
**FER**	-3.22246122761871	0.9	NA	-6.44492245523742	2	0.0457574905606751	NA	0.0457574905606751	U87	PTK	FER
**MER (MERTK)**	-4.15106174783928	0.984	NA	-41.5106174783928	10	0.00700490156865849	NA	0.00700490156865849	U87	PTK	MERMERTK
**RON**	-1.91123936581053	1	NA	-1.91123936581053	1	0.000174066157630127	NA	0	U87	PTK	RON
**TRKA (NTRK1)**	-3.19788565596013	0.96	NA	-9.59365696788039	3	0.0177287669604316	NA	0.0177287669604316	U87	PTK	TRKANTRK1
**TRKB (NTRK2)**	-3.2010453223583	0.98	NA	-12.8041812894332	4	0.00877392430750515	NA	0.00877392430750515	U87	PTK	TRKBNTRK2
**TRKC (NTRK3)**	-3.24699419075924	0.972	NA	-12.987976763037	4	0.0123337350737254	NA	0.0123337350737254	U87	PTK	TRKCNTRK3
**ADCK3 (LOC56997)**	-0.197714045621856	0.988	NA	-1.58171236497485	8	0.00524305541237188	NA	0.00524305541237188	U87	STK	ADCK3LOC56997
**CDK1 (CDC2)**	-0.363546633471559	0.98	NA	-4.72610623513027	13	0.00877392430750515	NA	0.00877392430750515	U87	STK	CDK1CDC2
**CDK2**	-0.505467359777815	0.958	NA	-8.59294511622286	17	0.0186344909214556	NA	0.0186344909214556	U87	STK	CDK2
**CDK7**	-0.367807438011474	0.92	NA	-2.57465206608032	7	0.0362121726544447	NA	0.0362121726544447	U87	STK	CDK7
**CDKL5 (STK9)**	-0.392686161233142	0.922	NA	-3.53417545109828	9	0.0352690789463706	NA	0.0352690789463706	U87	STK	CDKL5STK9
**DYRK1A**	-0.147494285528632	0.904	NA	-0.294988571057264	2	0.0438315695246367	NA	0.0438315695246367	U87	STK	DYRK1A
**ERK5 (MAPK7)**	-0.511897836811069	0.954	NA	-9.21416106259925	18	0.0204516252959049	NA	0.0204516252959049	U87	STK	ERK5MAPK7
**ERK7**	-	0.916	NA	-	5	0.038104526	NA	0.03810452	U8	S	ERK7BI91

**(BI916334)**	0.32418763281176			1.6209381640588		3321496		63321496	7	TK	6334
**ICK**	-0.215752930890892	0.926	NA	-0.863011723563567	4	0.0333890133180656	NA	0.0333890133180656	U87	STK	ICK
**IKKb (IKBKINASE)**	-0.0389367981234278	0.966	NA	-0.0778735962468556	2	0.0150228735845067	NA	0.0150228735845067	U87	STK	IKKIKBKINASE
**PDK1 (PDPK1)**	-0.0129499125567217	0.99	NA	-0.0258998251134434	2	0.00436480540245009	NA	0.00436480540245009	U87	STK	PDK1PDPK1
**PRKY**	-0.241255912169447	0.948	NA	-1.68879138518613	7	0.0231916626619338	NA	0.0231916626619338	U87	STK	PRKY
**mTOR/FRAP**	-0.474120309381999	0.902	NA	-4.74120309381999	10	0.0447934624580583	NA	0.0447934624580583	U87	STK	TORFRAP
Kinases activated by 5h in U87											
**kinaseName**	Normalized.Kinase.Stat	specificityScore	sensitivityScore	setStat	Peptides.in.Set	pSpecificityScore	pSensitivityScore	finalScore	Cell	type	gene
**ANPa (NPR1)**	0.179772047854754	0.996	NA	1.61794843069279	9	0.00174066157630127	NA	0.00174066157630127	U87	STK	ANPNPR1
**RSKL2**	0.0243858324842931	0.99	NA	0.0731574974528794	3	0.00436480540245009	NA	0.00436480540245009	U87	STK	RSKL2
**TAO1**	0.152720118727865	0.948	NA	0.152720118727865	1	0.0231916626619338	NA	0.0231916626619338	U87	STK	TAO1
**TAO2 (PSK)**	0.152720118727865	0.948	NA	0.152720118727865	1	0.0231916626619338	NA	0.0231916626619338	U87	STK	TAO2PSK
**TAO3 (JIK)**	0.152720118727865	0.948	NA	0.152720118727865	1	0.0231916626619338	NA	0.0231916626619338	U87	STK	TAO3JIK
Kinases inhibited by 5h in GBM											
**kinaseName**	Normalized.Kinase.Stat	specificityScore	sensitivityScore	setStat	Peptides.in.Set	pSpecificityScore	pSensitivityScore	finalScore	Cell	type	gene
**BTK**	-0.14301137611484	0.894	NA	-0.7150568805742	5	0.0486624812040823	NA	0.0486624812040823	GBM	PTK	BTK
**FES**	-0.141004900043953	0.914	NA	-0.705024500219764	5	0.0390538042661686	NA	0.0390538042661686	GBM	PTK	FES
**FGFR2**	-0.130906529744456	0.898	NA	-0.523626118977824	4	0.0467236633326956	NA	0.0467236633326956	GBM	PTK	FGFR2
**FGFR3**	-0.130906529744456	0.898	NA	-0.523626118977824	4	0.0467236633326956	NA	0.0467236633326956	GBM	PTK	FGFR3
**FGFR4**	-0.148618391866685	0.97	NA	-1.48618391866685	10	0.0132282657337552	NA	0.0132282657337552	GBM	PTK	FGFR4
**FRK**	-0.22459801850503	0.922	NA	-4.49196037010059	20	0.0352690789463706	NA	0.0352690789463706	GBM	PTK	FRK
**LTK**	-0.184464441450476	0.916	NA	-1.84464441450476	10	0.0381045263321496	NA	0.0381045263321496	GBM	PTK	LTK

**SRM (SRMS)**	-0.199412154791525	0.92	NA	-2.3929458574983	12	0.0362121726544447	NA	0.0362121726544447	GBM	PTK	SRMSRMS
**TEC**	-0.195201618765967	0.954	NA	-3.12322590025547	16	0.0204516252959049	NA	0.0204516252959049	GBM	PTK	TEC
**TYRO3**	-0.213601725982024	0.894	NA	-3.20402588973036	15	0.0486624812040823	NA	0.0486624812040823	GBM	PTK	TYRO3
Kinases activated by 5h in GBM											
**kinaseName**	Normalized.Kinase.Stat	specificityScore	sensitivityScore	setStat	Peptides.in.Set	pSpecificityScore	pSensitivityScore	finalScore	Cell	type	gene
**EPHA4**	0.0241269960765853	0.94	NA	0.0241269960765853	1	0.0268721464003014	NA	0.0268721464003014	GBM	PTK	EPHA4
**EPHB4**	0.0241269960765853	0.94	NA	0.0241269960765853	1	0.0268721464003014	NA	0.0268721464003014	GBM	PTK	EPHB4
**ANPa (NPR1)**	0.235421431876305	0.96	NA	1.88337145501044	8	0.0177287669604316	NA	0.0177287669604316	GBM	STK	ANPNPR1
**CDK4**	0.0234240872296013	0.938	NA	0.0234240872296013	1	0.0277971616209356	NA	0.0277971616209356	GBM	STK	CDK4
**DCAMKL2 (BI035543)**	0.285455680314429	0.92	NA	2.56910112282986	9	0.0362121726544447	NA	0.0362121726544447	GBM	STK	DCAMKL2BI035543
**DMPK2 (HSMDPKIN)**	0.0234240872296013	0.938	NA	0.0234240872296013	1	0.0277971616209356	NA	0.0277971616209356	GBM	STK	DMPK2HSMDPKIN
**DNAPK/PRKDC**	0.00694338014585466	0.976	NA	0.00694338014585466	1	0.0105501823333082	NA	0.0105501823333082	GBM	STK	DNAPKPRKDC
**NDR1 (NDR)**	0.0730733302569579	0.994	NA	0.292293321027832	4	0.00261361560268669	NA	0.00261361560268669	GBM	STK	NDR1NDR
**PKACa (PRKACA)**	0.368846323948465	0.93	NA	9.95885074660854	27	0.0315170514460649	NA	0.0315170514460649	GBM	STK	PKACPRKACA
**PKACb PRKACB**	0.354125467053165	0.954	NA	9.20726214338228	26	0.0204516252959049	NA	0.0204516252959049	GBM	STK	PKACPRKACB
**RSK1 (RPS6KA2)**	0.332039937326592	0.936	NA	5.64467893455206	17	0.0287241512618947	NA	0.0287241512618947	GBM	STK	RSK1RPS6KA2
**RSK2 (RPS6KA3)**	0.314722567144657	0.974	NA	6.6091739100378	21	0.0114410431213845	NA	0.0114410431213845	GBM	STK	RSK2RPS6KA3
**RSK3 (RPS6KA1)**	0.34901019558522	0.912	NA	6.9802039117044	20	0.0400051616715838	NA	0.0400051616715838	GBM	STK	RSK3RPS6KA1
**RSK4 (RPS6KA6)**	0.332918986443589	0.934	NA	5.9925417559846	18	0.0296531237699066	NA	0.0296531237699066	GBM	STK	RSK4RPS6KA6

7. Effect of DMSO on Akt, ERK1/2 and AMPKα phosphorylation on U87 cells (**Figure S1**)

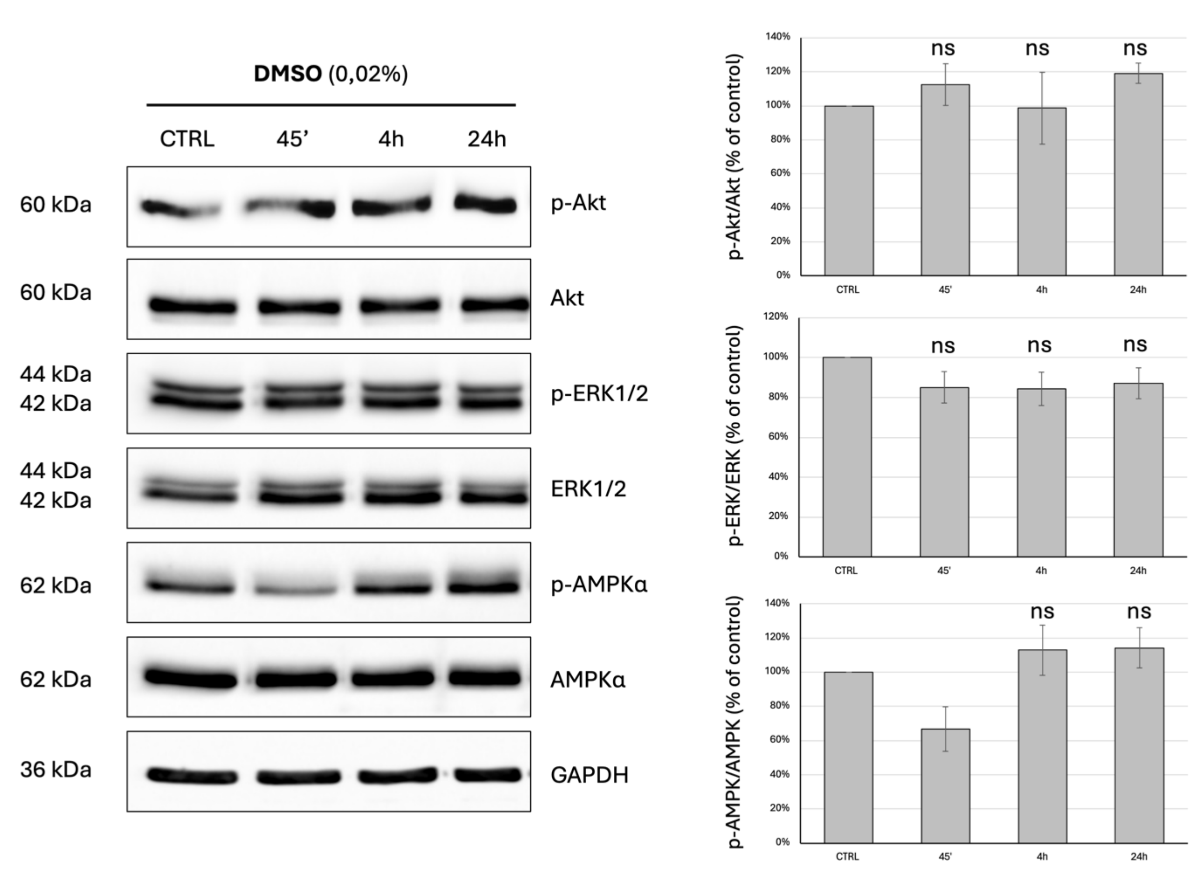

Figure S1. Cells were treated with complemented MEM medium containing 0.02% DMSO, the volume added as a co-solvent in all experiments with the tested hybrids. After incubation for varying times at 37 °C, band intensities (left panel) were quantified using ImageJ and are expressed as the ratio of phosphorylated protein/total protein (right panel). CTRL corresponds to initial time point. Values represent the mean ± SEM of three independent experiments made in triplicate. Statistical analyses of the data were performed using one-way analysis of variance (ANOVA), followed, if significant ($P < 0.05$), by *a posteriori* Dunnett's multiple comparisons test. Differences between groups were found not significant: ns

8. Original, uncropped Western blot images (**Figure S2-10**)

8.1 Western blot source images for Akt (**Figure S2**)

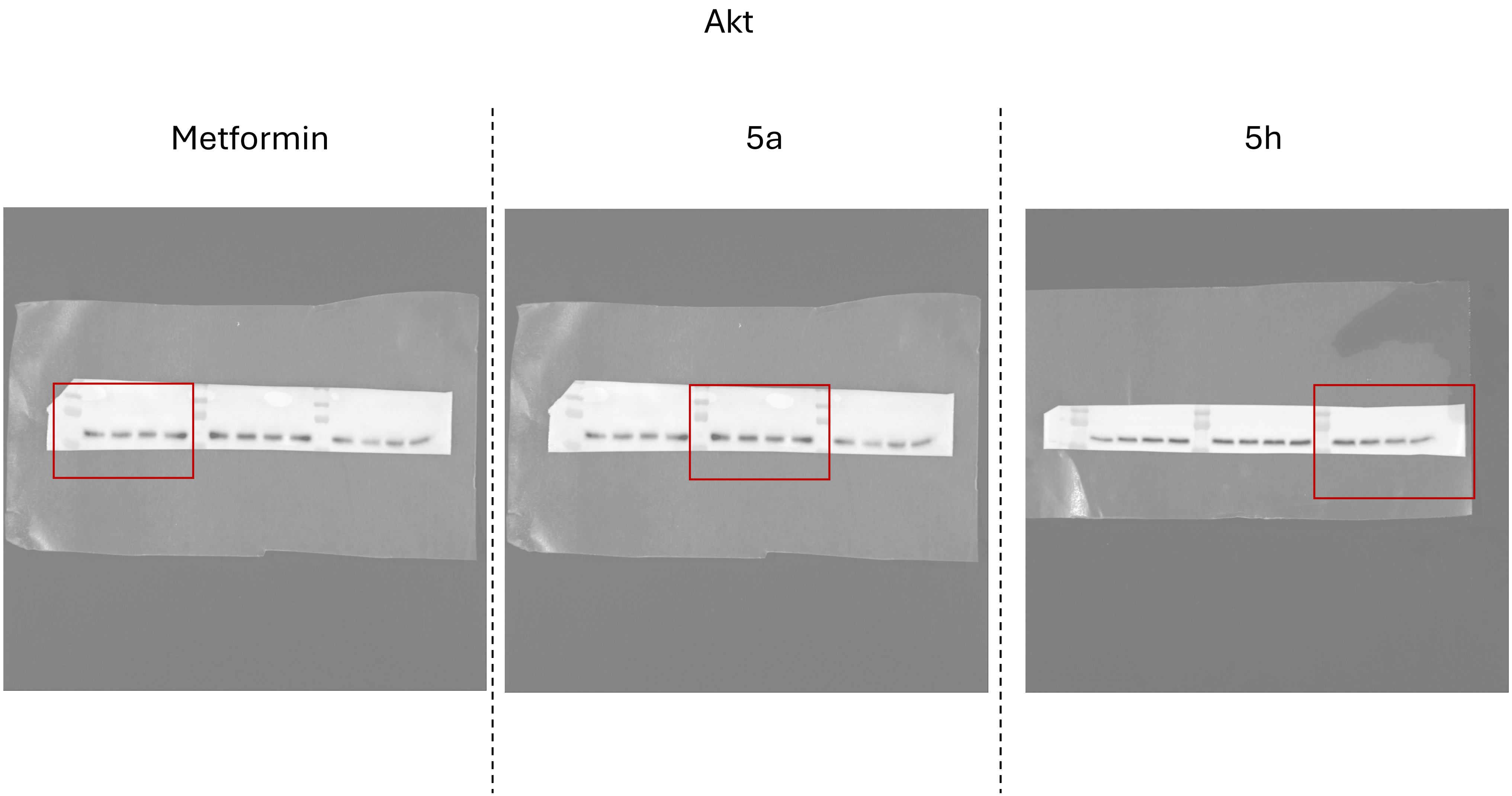

8.2 Western blot source images for P-Akt (**Figure S3**)

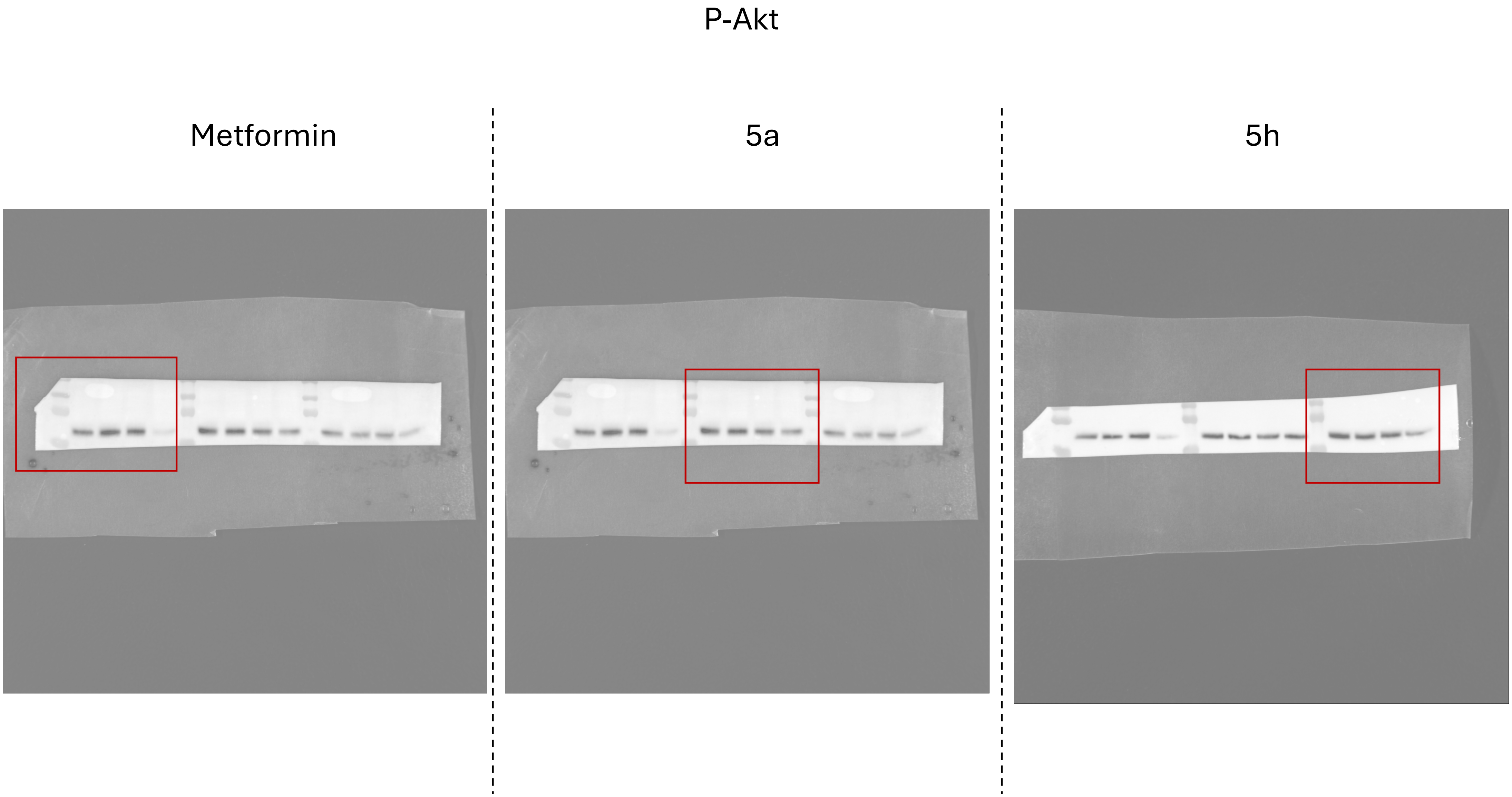

8.3 Western blot source images for GAPDH (loading control for Akt and P-Akt) (**Figure S4**)

GAPDH

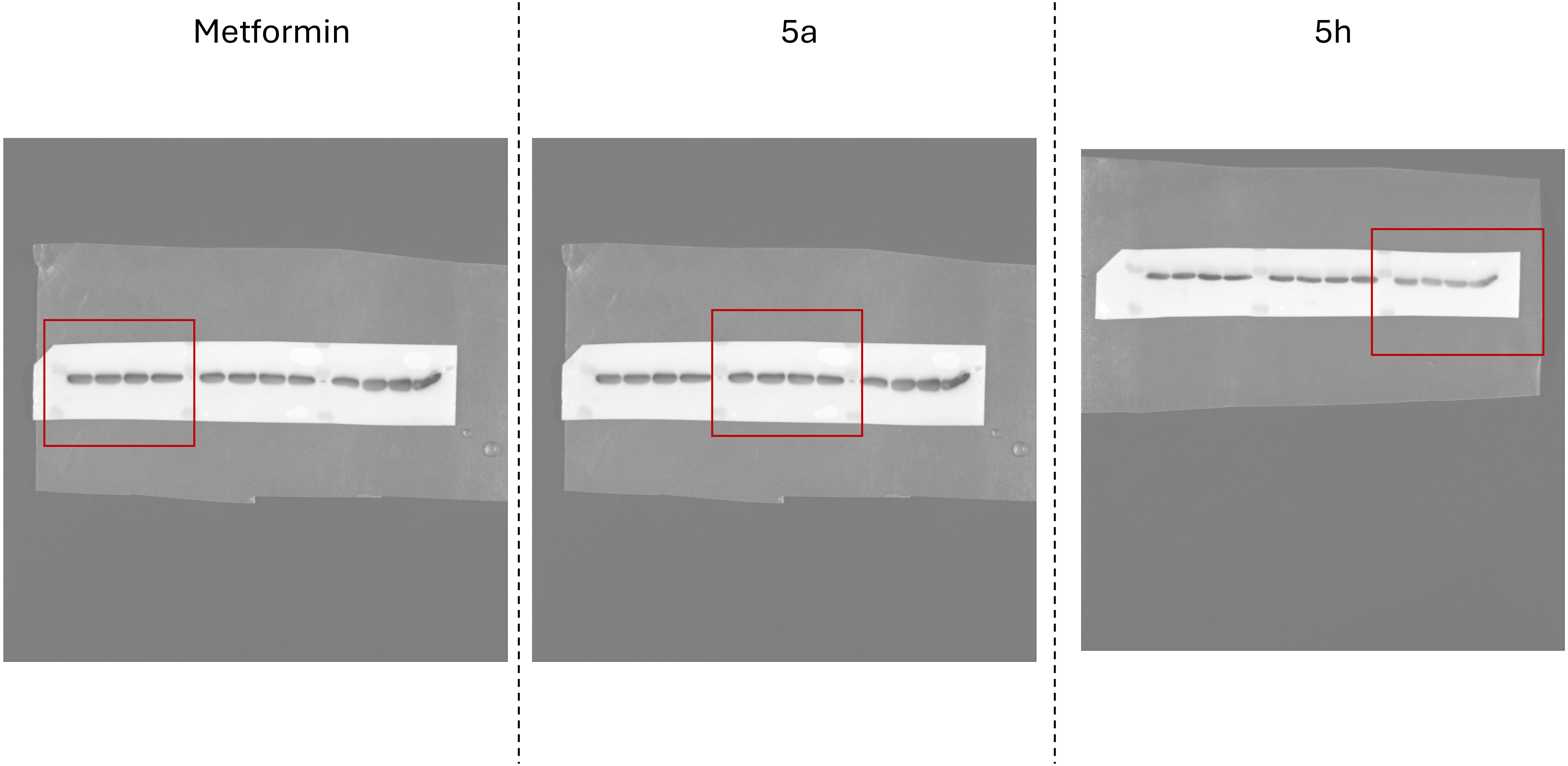

8.4 Western blot source images for AMPK (**Figure S5**)

AMPK

Metformin

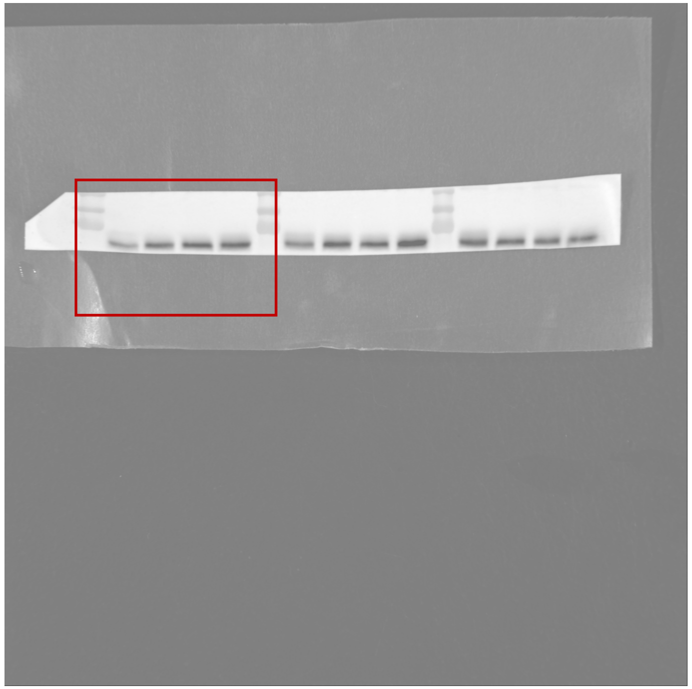

5a

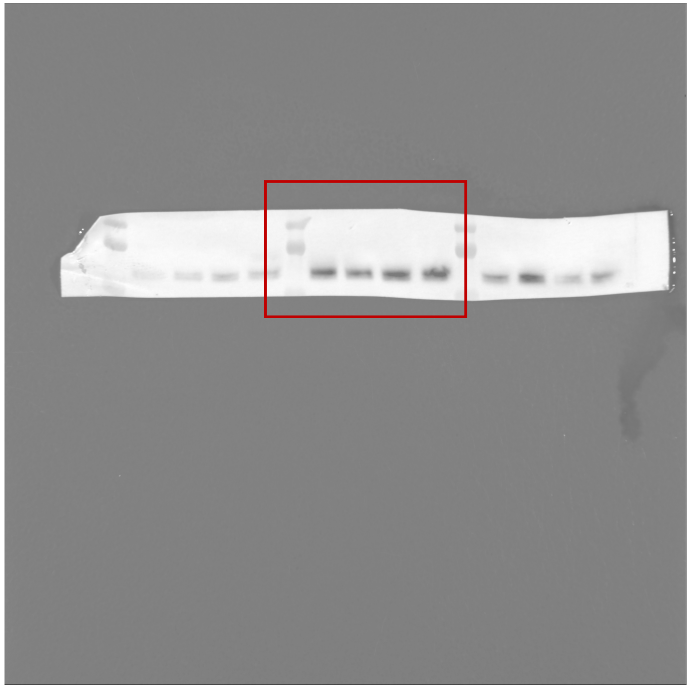

5h

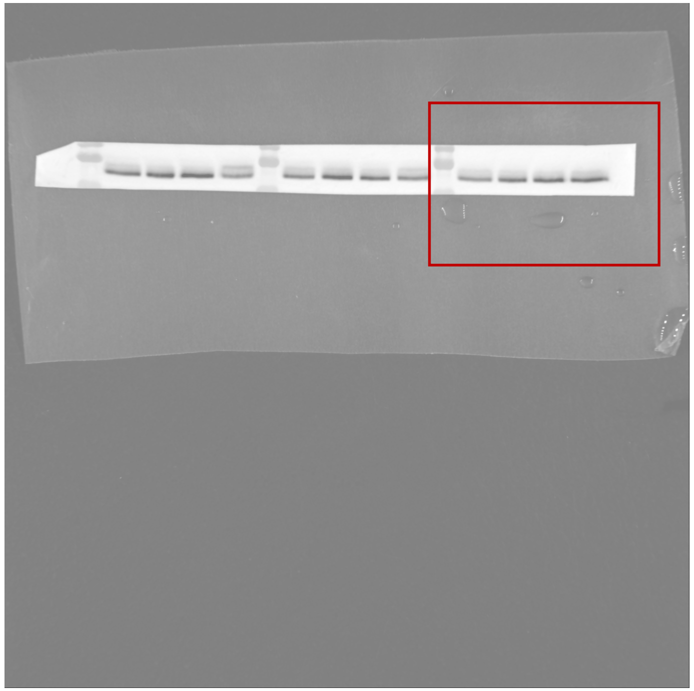

8.5 Western blot source images for P-AMPK (**Figure S6**)

P-AMPK

Metformin

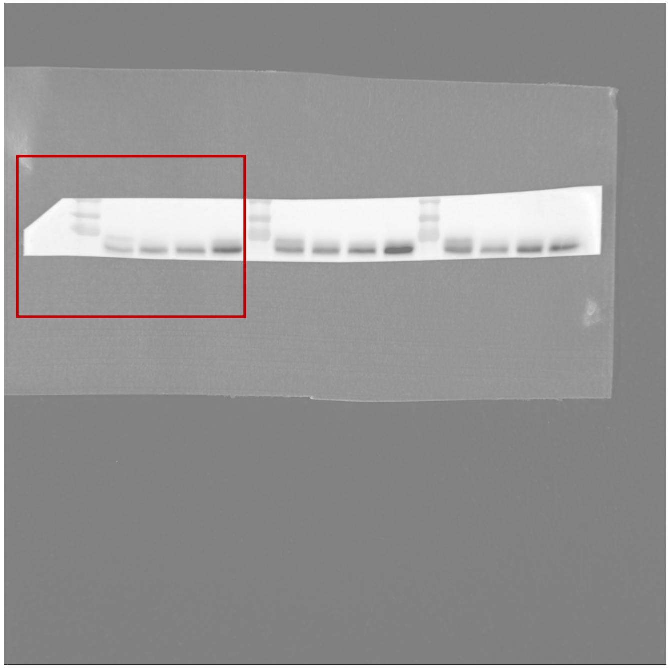

5a

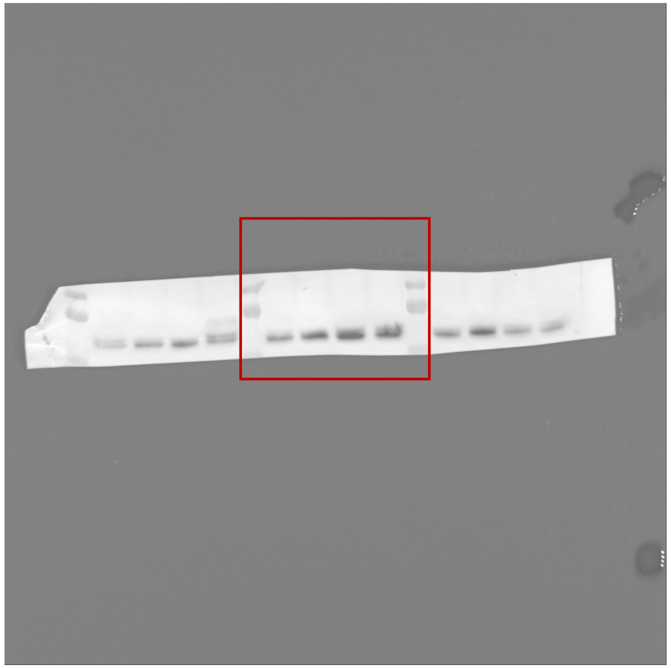

5h

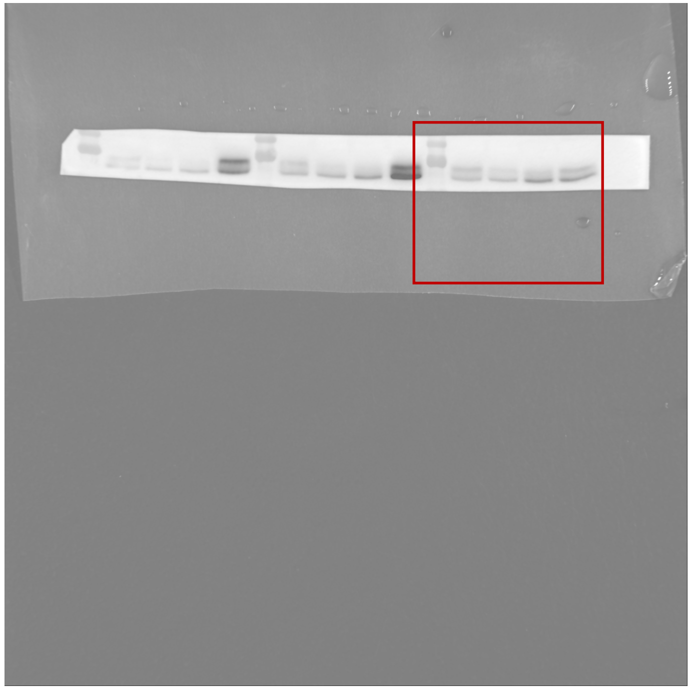

8.6 Western blot source images for GAPDH (loading control for AMPK and P-AMPPK) (**Figure S7**)

GAPDH

Metformin

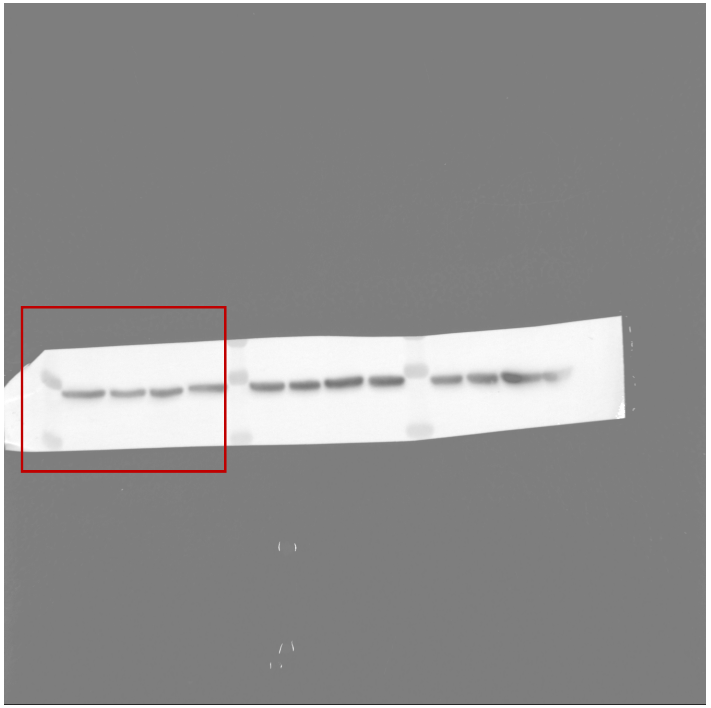

5a

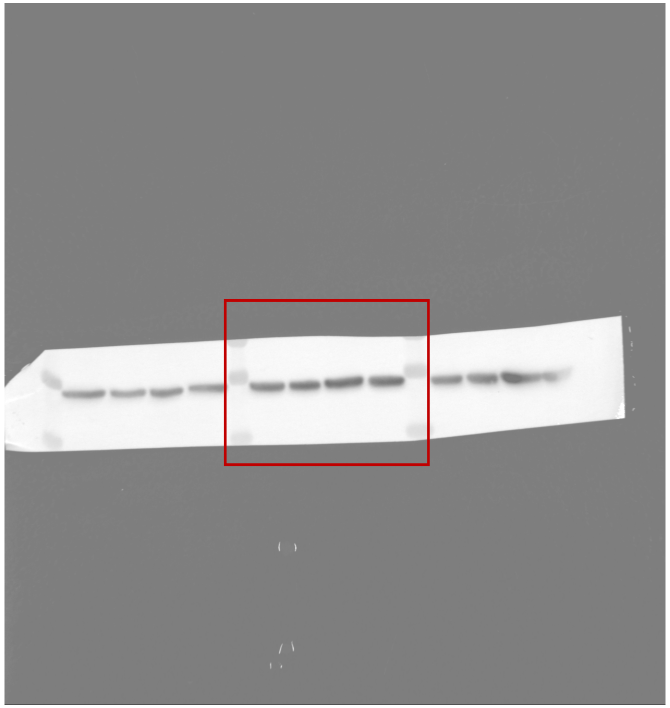

5h

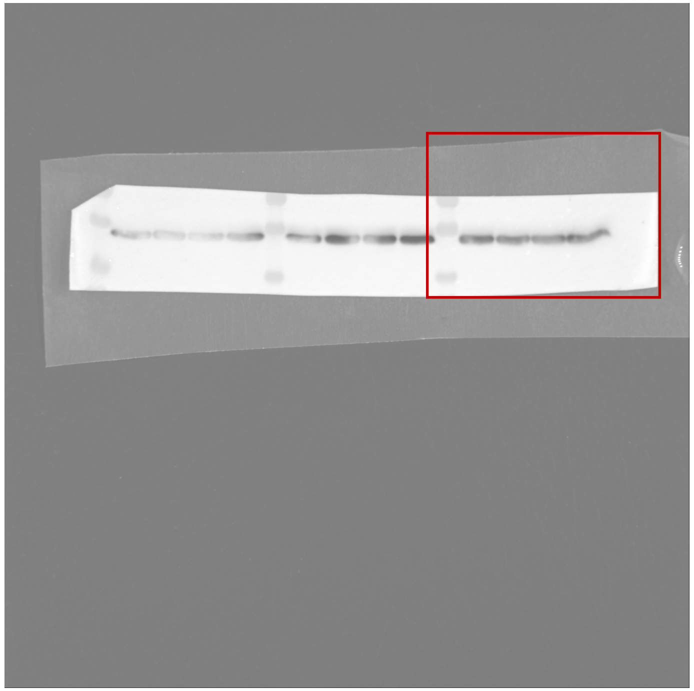

8.7 Western blot source images for ERK1/2 (**Figure S8**)

ERK1/2

Metformin

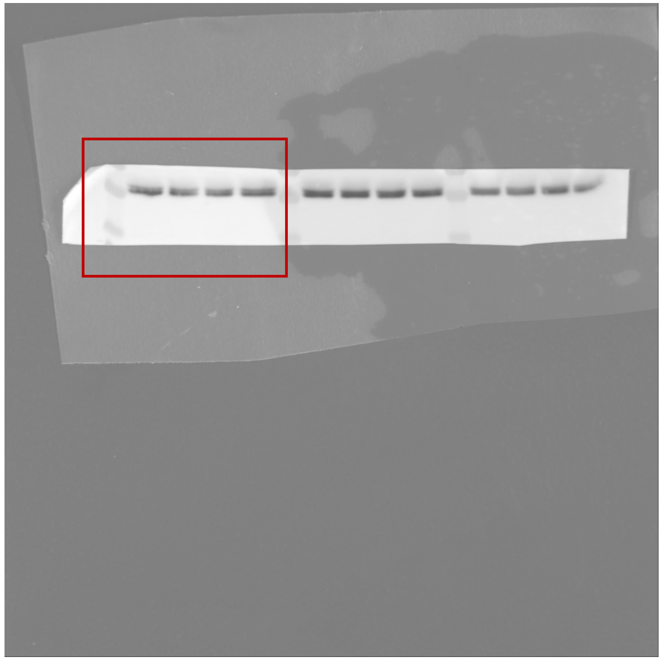

5a

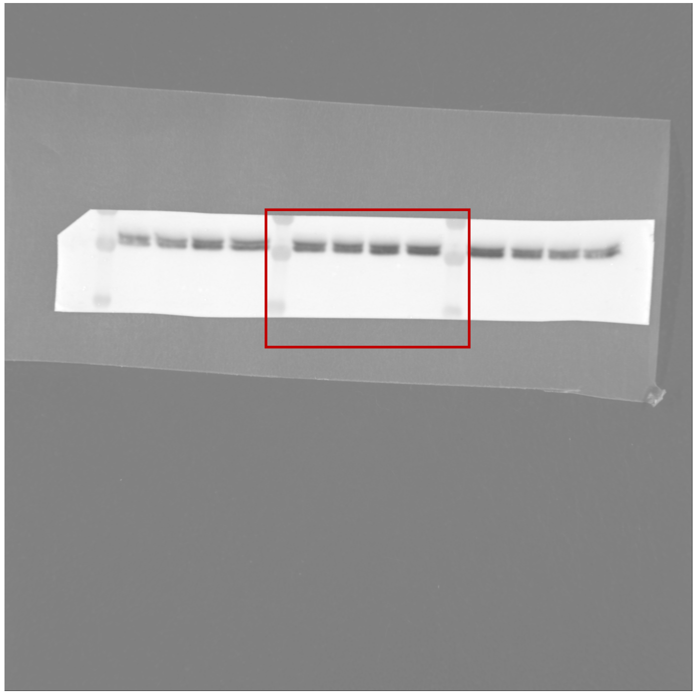

5h

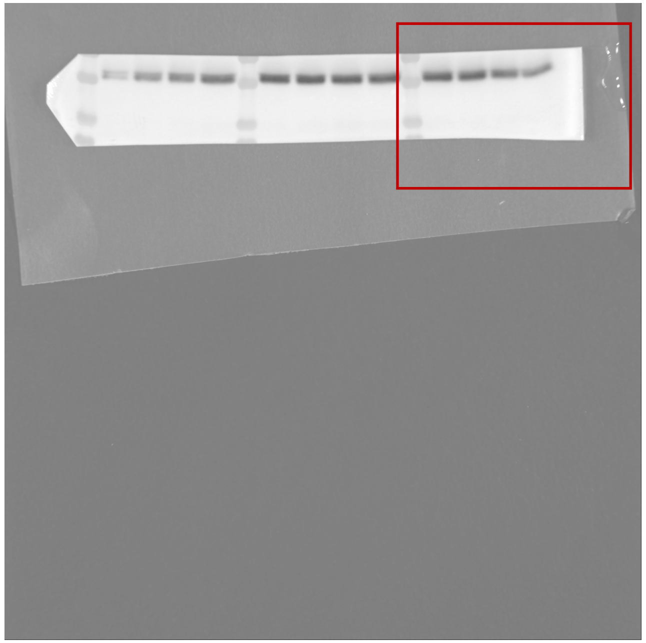

8.8 Western blot source images for P-ERK1/2 (**Figure S9**)

P-ERK1/2

Metformin

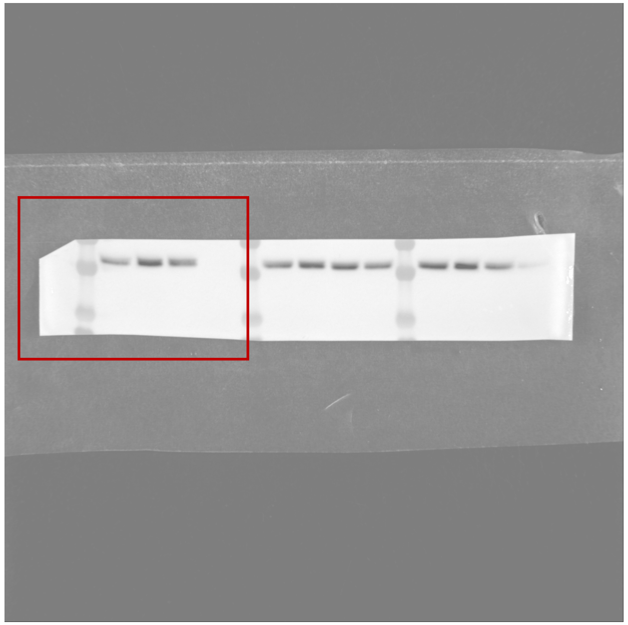

5a

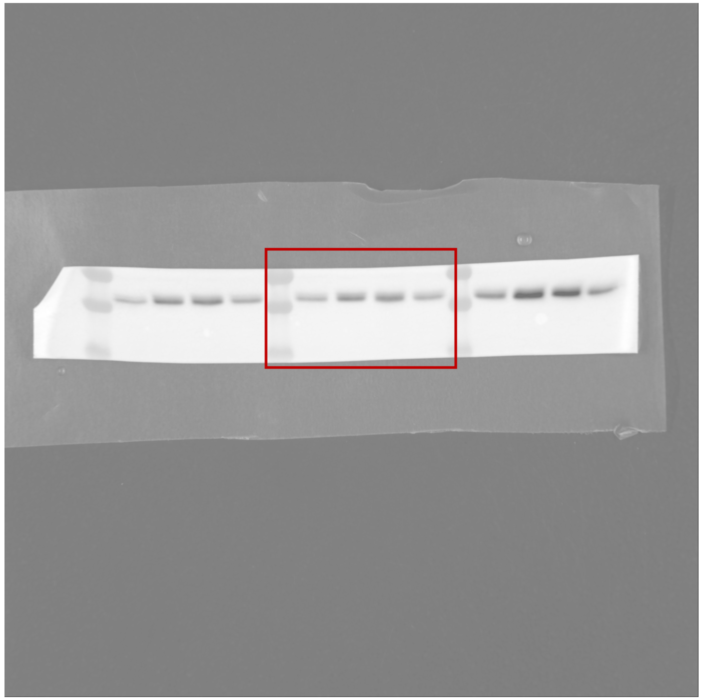

5h

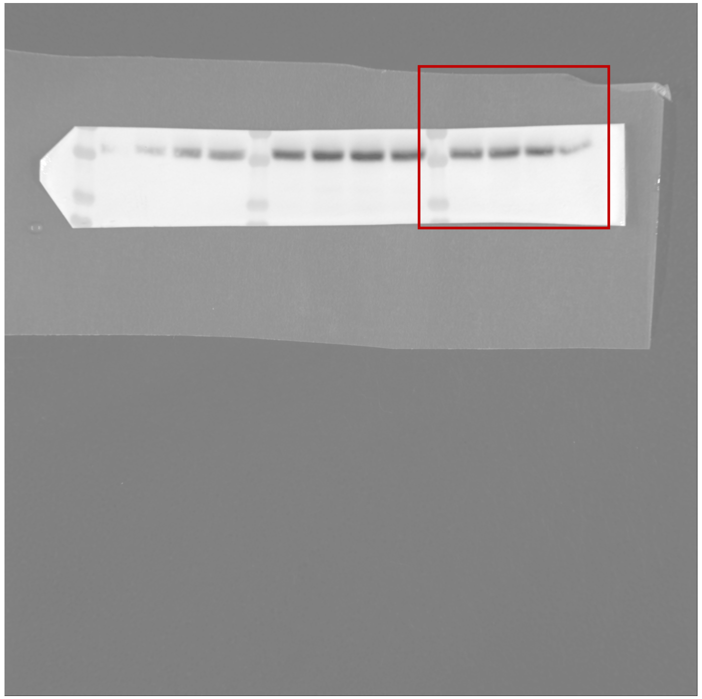

8.9 Western blot source images for GAPDH (loading control for ERK1/2 and P-ERK1/2) (**Figure S10**)

Metformin

GAPDH

5a

5h

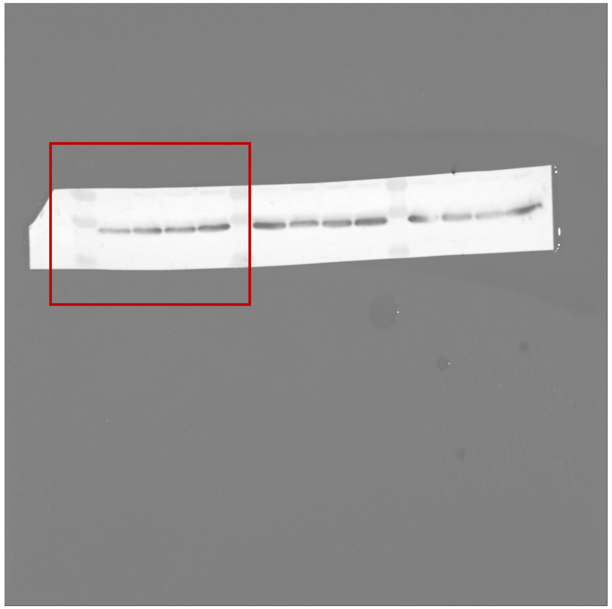

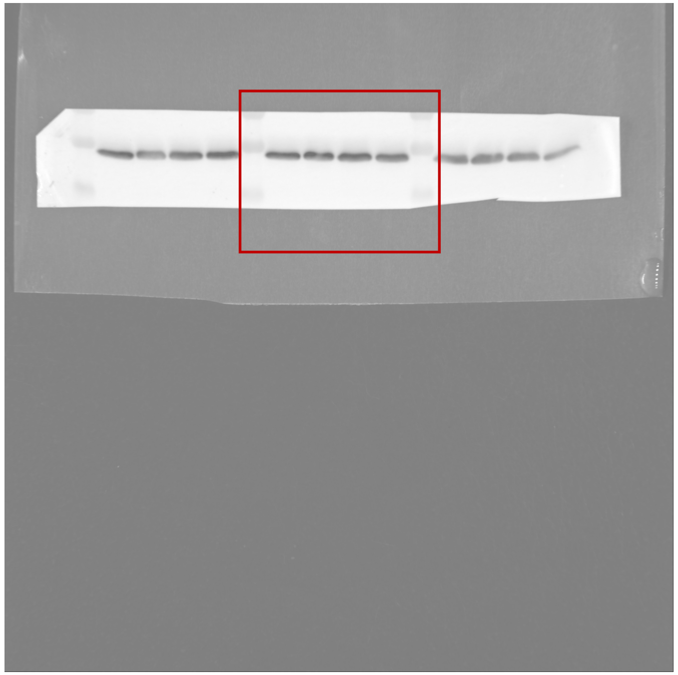

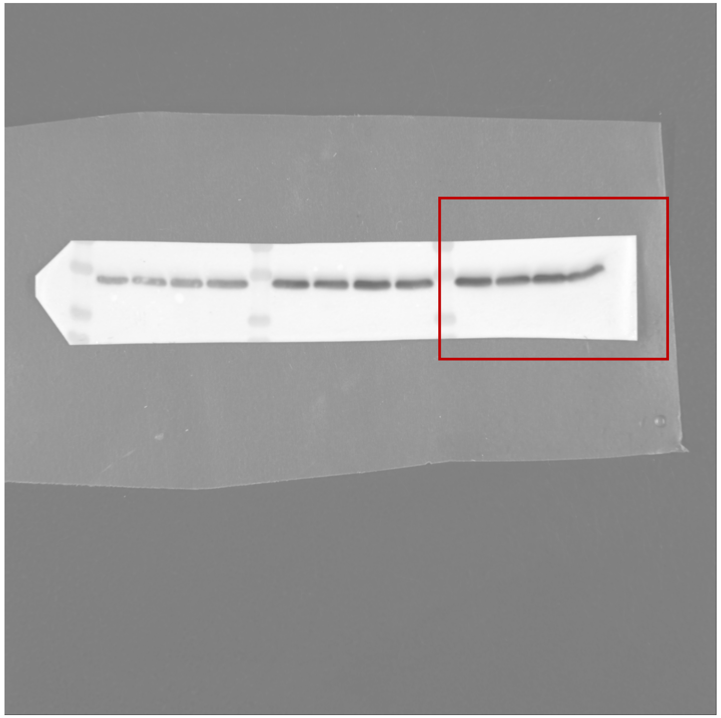

References

[1] Rouen, M.; Chaumont, P.; Barozzino-Consiglio, G.; Maddaluno, J.; Harrison-Marchand, A. Chiral Lithium Amido Zincates for Enantioselective 1,2-Additions: Auto-assembling Reagents Involving a Fully Recyclable Ligand. *Chem. Eur. J.* **2018**, *24*, 9238 – 9242. DOI: 10.1002/chem.201802044

[2] García-Díaz, M.; Sánchez-García, D.; Soriano, J.; Sagristà, L.; Mora, M.; Villanueva, A.; Stockert, J.; Cañete, M.; Nonell, S. Temocene: the porphycene analogue of temoporfin. *Med. Chem. Commun.* **2011**, *2*, 616-619. DOI: 10.1039/C1MD00065A

[3] Amslinger, S.; Lindner, S. Limno-CP: A Natural Product-inspired 5-Aryl-3(2H)-furanone as Scaffold for a Library of α-α-α-Modified Enones. *Synthesis.* **2011**, *16*, 2671–2683. DOI: 10.1055/s-0030-1260115

[4] Fei, Z.; McDonald, F. Stereo- and Regioselective Glycosylations to the Bis-*C*-arylglycoside of Kidamycin. *Org. Lett.* **2007**, *9, 18,* 3547–3550. DOI: 10.1021/ol7014219

[5] Hasse, K.; Willis, A.C.; Banwell, M.G. Modular Total Syntheses of Lamellarin G Trimethyl Ether and Lamellarin S. *Eur. J. Org. Chem.* **2011**, 88–99. DOI: 10.1002/ejoc.201001133

[6] Geske, L.; Baier, J.; Boulos, J.C.; Efferth, T.; Opatz, T. Xylochemical Synthesis and Biological Evaluation of the Orchidaceous Natural Products Isoarundinin I, Bleochrin F, Blestanol K and Pleionol. *J. Nat. Prod.* **2023**, *86, 1,* 131–137. DOI: 10.1021/acs.jnatprod.2c00830

[7] Davidson, S.J.; Barker, D. Total Synthesis of Ovafolinins A and B: Unique Poly-cyclic Benzoxepin Lignans through a Cascade Cyclization. *Angew. Chem. Int. Ed.* **2017**, *56*, 9483 –9486. DOI: 10.1002/anie.201705575

[8] Lebedev, A.V.; Lebedeva, A.B.; Sheludyakov, V.D.; Kovaleva, E.A.; Ustinova, O.L.; Kozhevnikov, I.B. Competitive formation of β-amino acids, propenoic, and ylidenemalonic acids by the Rodionov reaction from malonic acid, aldehydes, and ammonium acetate in alcoholic medium. *Russ. J. Gen. Chem*. **2005**, *75*, 1113–1124. DOI: 10.1007/s11176-005-0377-9:

[9] Hiroshi, H.; Masayuki, I.; Hiroshi, K.; Yasuo, Y.; Yoshikatsu, N.; Teruji, K.; Yukihiko, F. JP 10259129. **1998**, Inhibitors of blood vessel development.

[10] Blicke, F.F.; Godt Jr, H.C. Local anesthetics. IV. The synthesis of local anesthetic 3,4-dihydroisoquinolines. *J. Am. Chem. Soc*. **1954**, *76*, 3656–3662. DOI: 10.1021/ja01643a017

[11] Rao, V.R.; Muthenna, P.; Shankaraiah, G.; Akileshwari, C.; Babu, K.H.; Suresh, G.; Babu, K.S.; Chandra Kumar, R.S.; Prasad, K.R.; Yadav, P.A.; Petrash, J.M.; Reddy, G.B.; Rao, J.M. Synthesis and biological evaluation of new piplartine analogues as potent aldose reductase inhibitors (ARIs). *Eur. J. Med. Chem.* **2012**, *57*, 344-61. DOI: 10.1016/j.ejmech.2012.09.014.

[12] Pasturel-Jacopé, Y.; Solladié, G.; Maignan, J. WO 03/024911 A1, **2003**, Method for preparing α-(phenyl)cinnamic acids and/or derivatives thereof and their use in cosmetic composition.

[13] Solladié, G.; Pasturel-Jacopé, Y.; Maignan, J. A re-investigation of resveratrol synthesis by Perkins reaction. Application to the synthesis of aryl cinnamic acids. *Tetrahedron* **2003**, *59*, 3315–3321. DOI: 10.1016/S0040-4020(03)00405-8
